# On-complex three-component cascade reactions involving phosphorescent cyclometalated Ir(iii) chloro-isocyanide complexes, nitriles, and propylamine

**DOI:** 10.1039/d6sc00822d

**Published:** 2026-03-25

**Authors:** Son N. T. Phan, Vinh Q. Dang, Thomas S. Teets

**Affiliations:** a Department of Chemistry, University of Houston 3585 Cullen Blvd, Room 112 Houston TX 77204-5003 USA tteets@uh.edu

## Abstract

An “on-complex” three-component cascade reaction involving cyclometalated iridium chloro-isocyanide complexes, nitriles, and propylamine is reported. This ligand-based functionalization approach gives access to phosphorescent bis-cyclometalated iridium compounds containing a new chelating imino-acyclic diamino carbene (imino-ADC) ligand, not accessible by traditional organometallic synthesis. The reaction is compatible with alkyl nitriles and iridium complexes bearing different cyclometalating ligands and aromatic isocyanides. The imino-ADC ligand in the resulting cationic products can be deprotonated in a separate step to give the corresponding neutral compounds. The products of the cascade reactions were obtained in low to moderate yields, depending on the isocyanide and nitrile substituents used, with their structures characterized by multinuclear NMR and single-crystal X-ray diffraction. Photophysical measurements show that the complexes described here exhibit blue phosphorescence, with the spectral profile dependent on the cyclometalating ligand, which is either 2,4-difluorophenylpyridine (F_2_ppy) or 1-phenyl-1*H*-pyrazole (ppz). The three-component reaction developed here expands the limited library of organometallic multicomponent reactions and gives access to a new structure type for phosphorescent bis-cyclometalated iridium complexes.

## Abstract 2

Three-component cascade reactions involving bis-cyclometalated iridium isocyanide precursors, propylamine, and an alkyl nitrile furnish imino-acyclic diamino carbene products, further modifiable by deprotonation.

## Graphical abstract

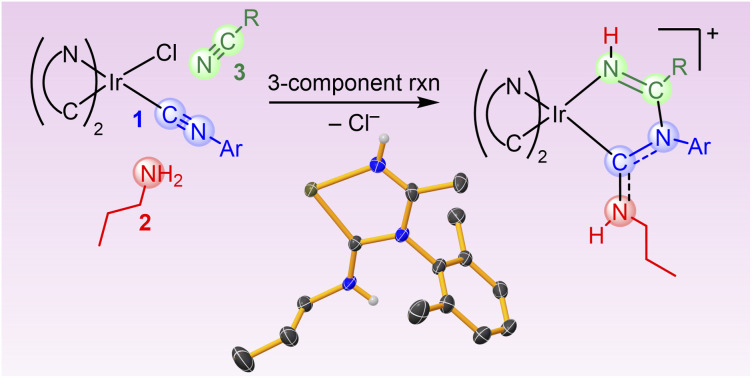

## Introduction

Luminescent organometallic iridium(iii) complexes that phosphoresce from triplet excited states have found numerous applications, such as photocatalysis,^1–3^ bioimaging,^4–6^ sensing,^7–9^ and particularly optoelectronics,^10–12^ where the strong spin–orbit coupling (SOC) induced by the iridium center makes these complexes standout performers.^13,14^ Due to synthetic simplicity, for optically active organoiridium complexes the most common structure type is comprised of heteroleptic bis-cyclometalated Ir(iii) compounds, with two bidentate cyclometalating ligands that dictate the triplet excited state-energy and consequently the emission profile.^15^ The two remaining coordination sites are occupied by one bidentate ancillary ligand or two monodentate ancillary ligands, which are critical determinants of the complex's physical and chemical properties, including excited-state dynamics that determine the phosphorescence metrics.^16^

**Scheme 1:**
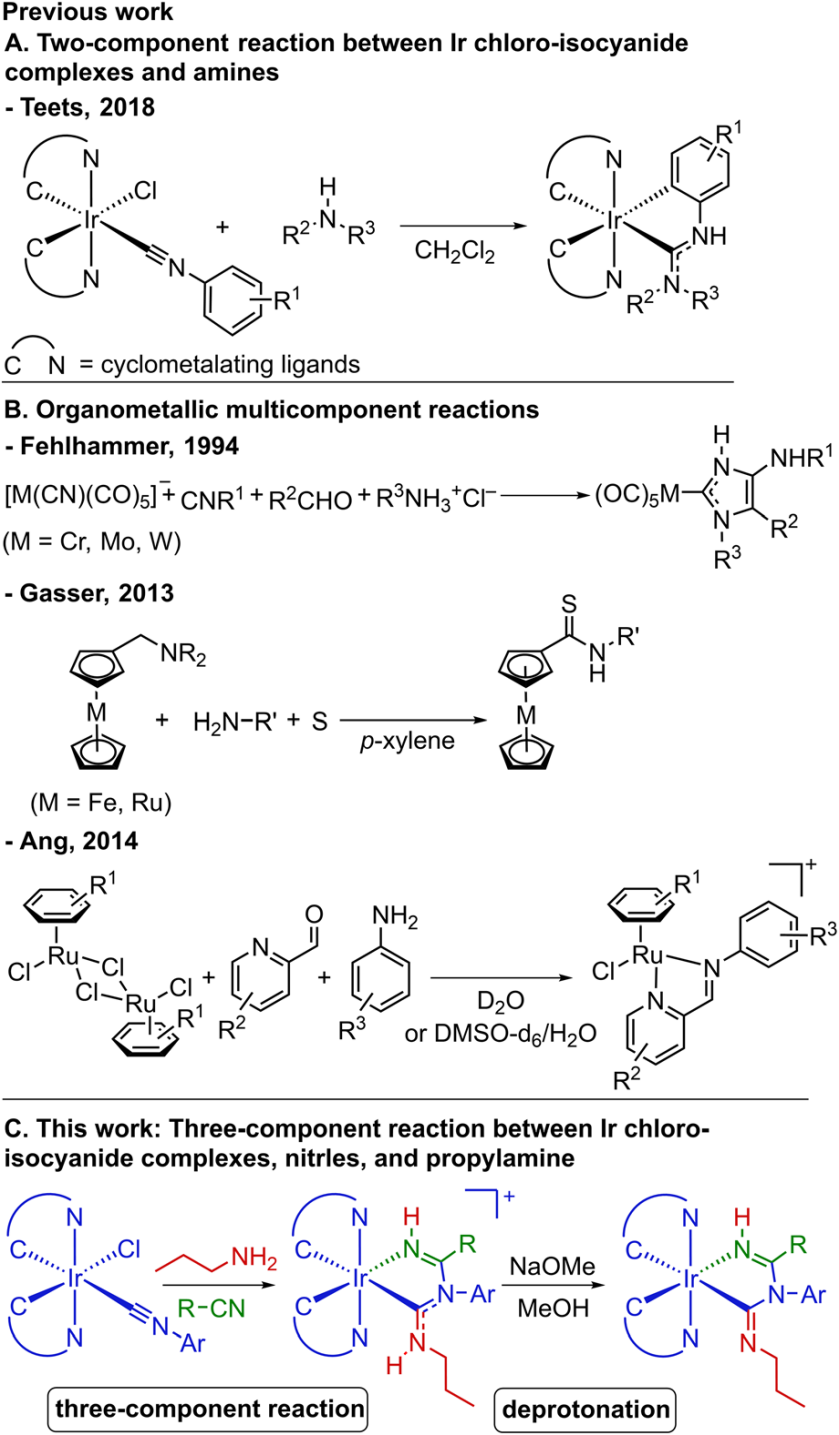
(A) Two-component reaction between Ir chloro-isocyanide complexes and amines; (B) representative reported organometallic multicomponent reactions; (C) three-component reaction and deprotonation of the products developed in this study.

Most often, the ancillary ligand(s) are installed *via* a ligand-substitution reaction on bis-cyclometalated iridium(iii) precursors. Tuning the photophysical properties of Ir(iii) emitters by modifying the ligands through “on-complex” ligand-based functionalization strategies have also been developed, which include cross-coupling reactions to modify cyclometalating ligands,^17,18^ coordination of boranes to cyano ligands,^19,20^ construction of bidentate ligands by nucleophilic addition to alkynyl ligands,^21–23^ and acid–base chemistry.^24,25^ Another ligand-based strategy, which has become prominent in the field of phosphorescent organometallics, is the installation of acyclic diamino carbenes (ADCs) by nucleophilic addition of amines to isocyanide ligands, developed by our group in the context of blue phosphorescence^26–28^ and by several other groups for other applications.^29–34^ An example of such a transformation is shown in Scheme 1A.^27^ ADCs, being strong σ donors, are effective at suppressing the thermal population of detrimental triplet metal-centered (^3^MC) excited states, producing blue-emitting Ir complexes with high photoluminescence quantum yields (*Φ*_PL_).^35^

Relevant to the subject of this work, multicomponent reactions (MCRs) represent a broad class of transformations found mainly in the context of organic synthesis. These reactions involve at least three reactants in a one-pot reaction, with most of the atoms incorporated into the final product,^36^ and those that involve at least two sequential bond-forming events can also be classified as “cascade” or “domino” reactions. There are many MCRs prominent in organic synthesis such as Strecker,^37^ Hantzsch,^38^ Biginelli,^39^ Mannich,^40^ and Ugi^41^ reactions and their variations. However, while MCRs catalyzed or mediated by transition-metal organometallics have been well studied,^42–58^ those occurring directly on the organometallic complexes to yield products containing newly formed ligands (*i.e.*, “on-complex” reactions) remain rare.^59–67^ Some of the earliest work in this category was reported by Fehlhammer and colleagues.^59–61^ As shown in Scheme 1B, one example is a four-component reaction involving chromium, molybdenum, or tungsten cyano complexes, isocyanides, aldehydes, and amine hydrochlorides, affording 4-aminoimidazolin-2-ylidene complexes.^60^ Also displayed in Scheme 1B is a more recent work by Gasser and co-workers, in which they developed a three-component reaction comprising (aminomethyl)ferrocene or ruthenocenyl analogues, amines, and elemental sulfur to give ferrocenyl or ruthenocenyl thioamides.^62^ Synthesis of ruthenium–arene Schiff base complexes *via* a water-promoted three-component reaction between ruthenium–arene complexes, picolinaldehydes, and aniline derivatives has been realized by Ang and colleagues (Scheme 1B).^64^ These reactions have good step-economy and in some cases enable the synthesis of complexes with unusual ligand structures that would not be attainable using more traditional routes, but they have not been applied to the design of next-generation phosphorescent organometallic compounds.

The subject of this work is a novel three-component cascade reaction that occurs on bis-cyclometalated iridium complexes, discovered serendipitously while exploring the synthesis of new blue-phosphorescent Ir compounds supported by ADCs. This reaction involves bis-cyclometalated Ir(iii) chloro-isocyanide complexes, nitriles, and propylamine, and is summarized generally in Scheme 1C. In this reaction, a two-step cascade nucleophilic addition involving propylamine, the isocyanide ligand, and the nitrile, accompanied by substitution of the chloro ligand, affords the product bearing a new chelating imino-ADC ligand. This paper describes the optimization of these reactions and the substrate scope, varying the isocyanide substituent and the nitrile. The products contain acidic protons on the imino-ADC ancillary ligand, enabling further derivatization *via* deprotonation. A total of eight complexes is described, six cationic products prepared *via* the three-component cascade reaction, and two of the deprotonated neutral analogues. The reported compounds all exhibit blue phosphorescence, and this work also includes an account of their photophysical properties, with the ancillary ligand having a significant effect on the excited-state dynamics. This work advances the field of organometallic MCRs and also represents a significant synthetic development in the design of phosphorescent cyclometalated Ir(iii) complexes.

## Results and discussion

### Synthesis

**Scheme 2:**
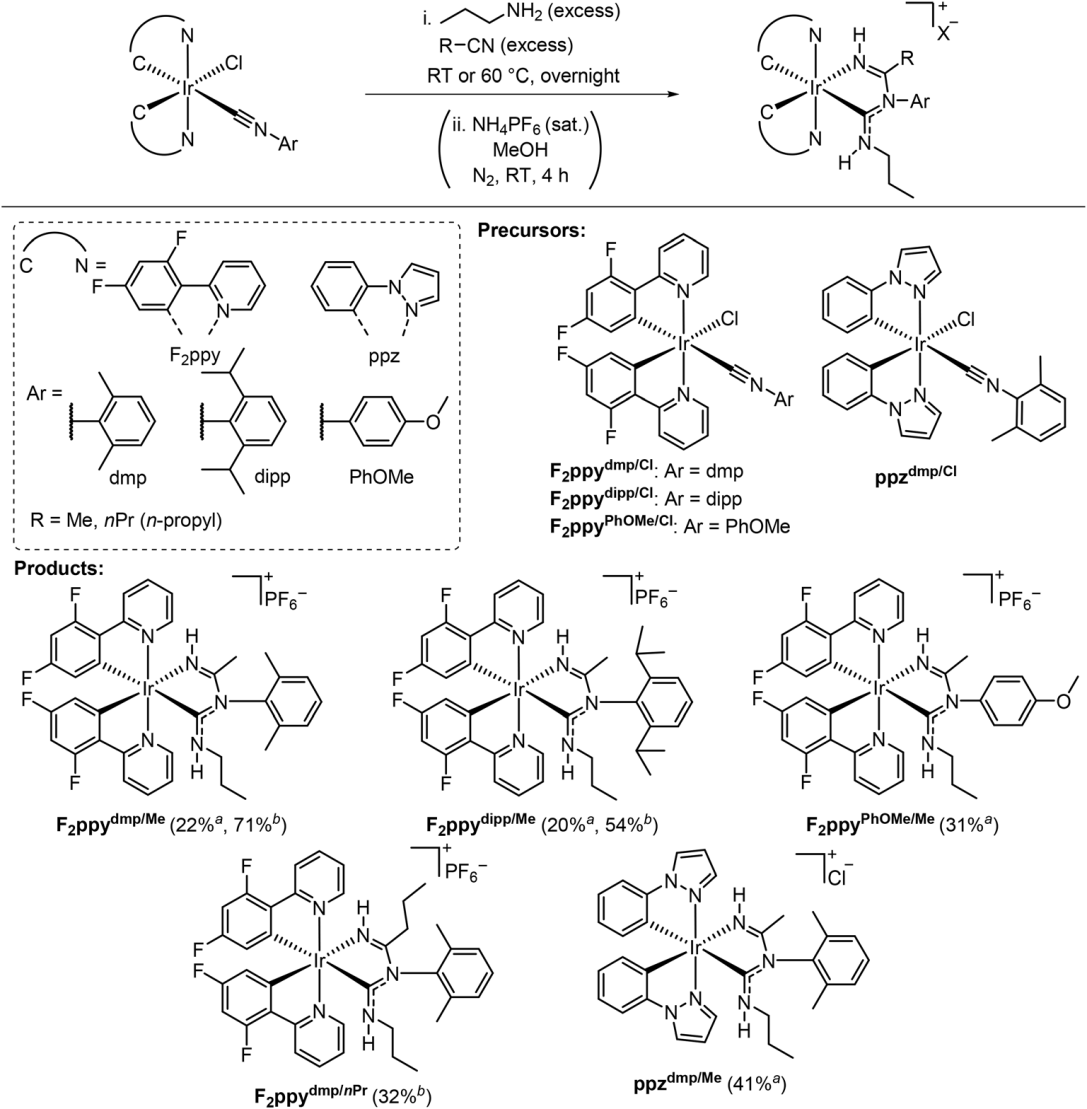
Synthesis of Ir complexes containing chelating imino-ADC ancillary ligands *via* a three-component reaction involving propylamine and nitriles. ^*a*^Reaction conditions: Ir chloro-isocyanide compound (0.18 g), propylamine (1.0 mL), nitrile (5.0 mL), room temperature. ^*b*^Reaction conditions: Ir chloro-isocyanide compound (0.10 g), propylamine (0.5 mL), nitrile (3.0 mL), 60 °C.

The three-component reaction and its scope are summarized in Scheme 2. The precursor Ir chloro-isocyanide complexes of the type Ir(C^N)_2_(Cl)(CNAr) (C^N = cyclometalating ligand) react with excess amounts of nitrile and propylamine (^*n*^PrNH_2_). The products contain a new chelating ancillary ligand composed of an ADC and an imine donor. The six examples in Scheme 2 are abbreviated as **C^N**^**Ar/R**^, based on the C^N ligand of the iridium complex (F_2_ppy = 2-(2,4-difluorophenyl)pyridine or ppz = 1-phenylpyrazole), the aryl (Ar) group from the isocyanide (dmp = 2,6-dimethylphenyl, dipp = 2,6-diisopropylphenyl, and PhOMe = 4-methoxyphenyl), and the R group from the nitrile (Me = methyl and *n*Pr = *n*-propyl). The nitrile and propylamine effectively serve as cosolvents, and the bis-cyclometalated iridium starting material remained intact when only a stoichiometric amount of the amine was used. The reactions readily occur at room temperature or 60 °C. A counterion exchange (chloride to hexafluorophosphate) was necessary for the products with F_2_ppy as the cyclometalating ligand to improve their solubility in common solvents used for photophysical measurements and facilitate purification by column chromatography. Five complexes with C^N = F_2_ppy and one with C^N = ppz were prepared, which were isolated and purified by column chromatography, recrystallization, or a combination of both methods. ^1^H NMR spectra with ^13^C{^1^H} (C^N = ppz) or ^19^F (C^N = F_2_ppy) NMR spectra, and high-resolution mass spectrometry data of all reported complexes are also given in the SI, Fig. S2–S29 and S38–S49, respectively.

Regarding the substrate scope, the reaction conditions tolerate both cyclometalating ligands investigated (F_2_ppy and ppz) and aryl isocyanides with sterically hindered (Ar = dmp or dipp) or electron-rich (Ar = PhOMe) aryl groups. Acetonitrile (MeCN) and butyronitrile (^*n*^PrCN) both react well, whereas benzonitrile (PhCN) promotes conversion of the chloro-isocyanide starting material but results in a mixture of several products. Despite our best efforts, only a very low yield of the desired product (F_2_ppy^dmp/Ph^, 2.0% isolated yield) was obtained after purification. Its molecular structure, determined by single-crystal X-ray diffraction, is shown in Fig. S1, SI. Experimental details are also provided in the SI. The outcomes of the reactions with different substrate combinations are dependent on the reaction temperature. The reactions of F_2_ppy^dmp/Cl^ or F_2_ppy^dipp/Cl^ with MeCN and ^*n*^PrNH_2_ at room temperature gave F_2_ppy^dmp/Me^ or F_2_ppy^dipp/Me^ in *ca.* 20% yield, respectively. At 60 °C, much higher yields (71% and 54%, respectively) were obtained for these two reactions. Similarly, the product F_2_ppy^dmp/^*^n^*^Pr^ was not observed at all in the room-temperature reaction, but was isolated in 32% yield when the temperature was increased to 60 °C. However, in the cases of F_2_ppy^PhOMe/Me^ and F_2_ppy^dmp/Ph^, the products were only obtained when the reaction was executed at room temperature. We observed intractable product mixtures when the same reactions were conducted at 60 °C and were unable to cleanly isolate any products for characterization.

**Scheme 3:**
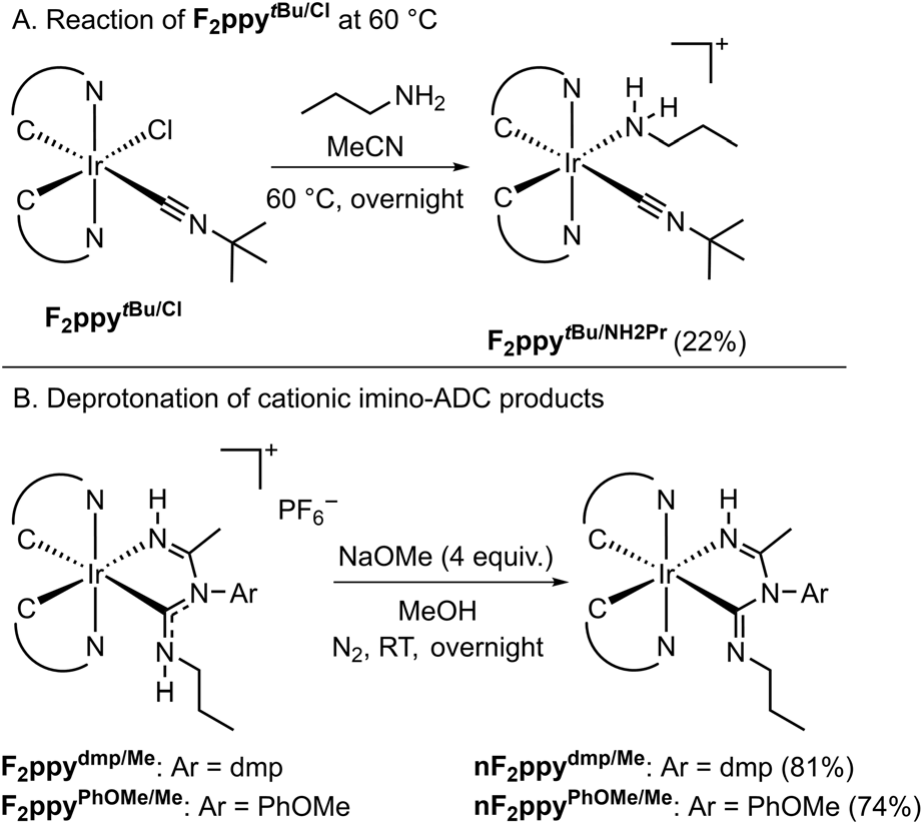
(A) Reaction of F_2_ppy*^t^*^Bu/Cl^ at 60 °C. (B) Deprotonation of F_2_ppy^dmp/Me^ and F_2_ppy^PhOMe/Me^ to give the neutral products with deprotonated chelating ligands.

Some substrate combinations failed to produce the desired imino-ADC product, in reactions carried out both at room temperature and 60 °C (Table S1). *Tert*-butylamine (^*t*^BuNH_2_) and diethylamine (Et_2_NH) as the nucleophile resulted in no reaction and recovery of the Ir precursor F_2_ppy^dmp/Cl^. Reactions between the sterically encumbered nitrile pivalonitrile (^*t*^BuCN), F_2_ppy^dmp/Cl^, and acetonitrile gave intractable crude mixtures, with no desired product observed. Similarly, the Ir precursor F_2_ppy^PhCF3/Cl^, which contains the electron-poor 4-(trifluoromethyl)phenyl isocyanide, was consumed during reactions with acetonitrile and propylamine, but a complex mixture of products resulted. Ir precursors bearing alkyl isocyanides, F_2_ppy^Bn/Cl^ with benzyl isocyanide and F_2_ppy*^t^*^Bu/Cl^ with *tert*-butyl isocyanide, are also not competent for the three-component reaction. At room temperature the Ir(iii) starting materials are recovered, whereas at 60 °C F_2_ppy*^t^*^Bu/Cl^ undergoes a ligand substitution reaction in which the chloride is replaced by ^*n*^PrNH_2_, generating the cationic product F_2_ppy*^t^*^Bu/NH2Pr^ (Scheme 3A). The molecular structure of F_2_ppy*^t^*^Bu/NH2Pr^, determined by single-crystal X-ray diffraction, is displayed in the SI, Fig. S1.

Based on the results above and previously reported work,^26–28,68,69^ a plausible mechanism is shown in the SI, Scheme S1. It is proposed that the reaction initiates with a nucleophilic attack of propylamine to the isocyanide ligand. The coordination of the isocyanide carbon to the Ir center increases its electrophilicity and facilitates this attack. After a proton-transfer step mediated by propylamine, nucleophilic attack of the formed ligand at the nitrile then occurs, which after another proton-transfer step forms a species containing an ADC with an imine arm. Finally, substitution of the chloro ligand by the imine affords the final cationic product with a bidentate imino-ADC ligand. The chloro ligand is not likely to be substituted by the nitrile at the outset, as suggested by the experiments where the chloro-isocyanide starting material is recovered (Table S1), and by the preferential formation of F_2_ppy*^t^*^Bu/NH2Pr^ rather than the nitrile complex (Scheme 3A).

Deprotonation reactions of two of the cationic products, F_2_ppy^dmp/Me^ and F_2_ppy^PhOMe/Me^, are described in Scheme 3B. NaOMe is used as a base to give the corresponding charge-neutral products nF_2_ppy^dmp/Me^ and nF_2_ppy^PhOMe/Me^ in good yields. Deprotonation occurs preferentially at the ADC instead of the imine; electron density corresponding to the imine hydrogen atom was located in the different map when solving the X-ray crystal structure of nF_2_ppy^PhOMe/Me^ (see below).

### Crystal structures

**Fig. 1:**
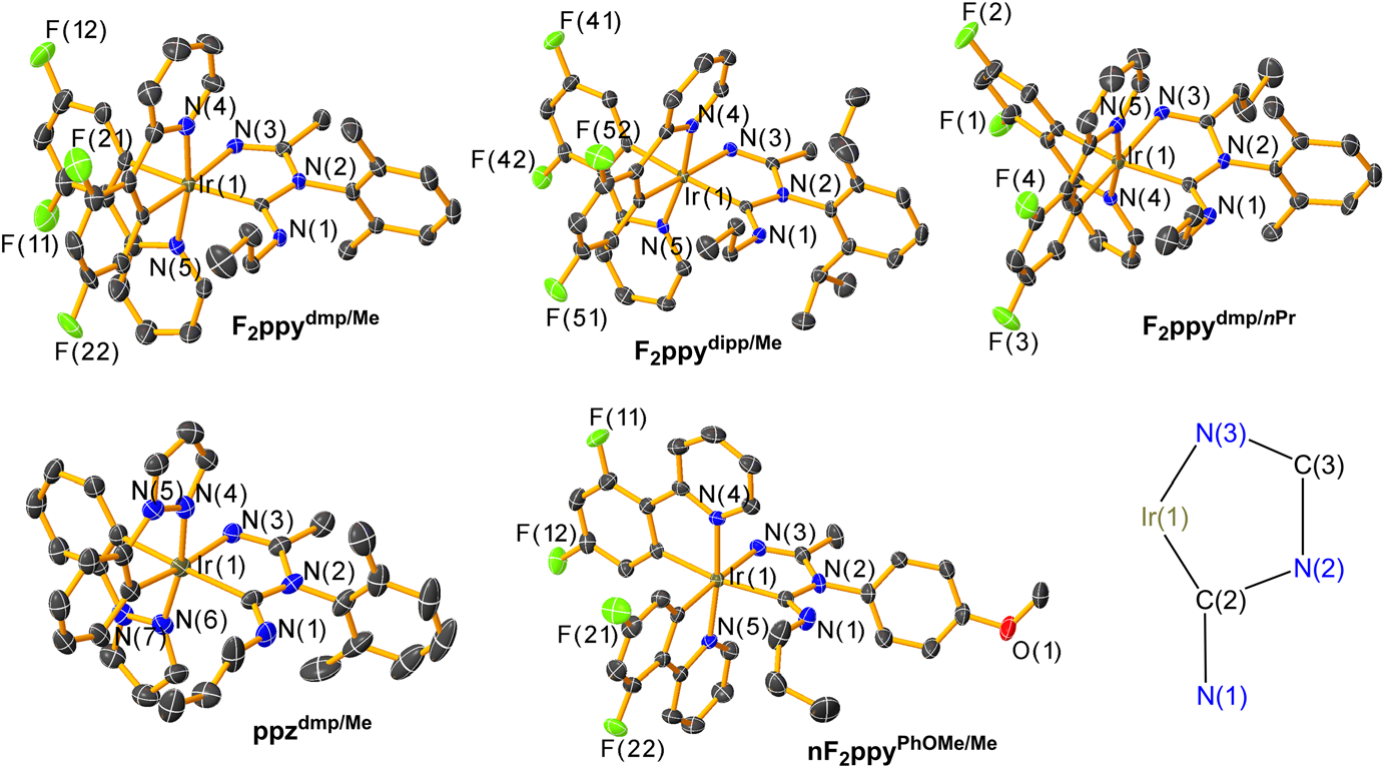
Molecular structures of complexes F_2_ppy^dmp/Me^, F_2_ppy^dipp/Me^, F_2_ppy^dmp/^*^n^*^Pr^, ppz^dmp/Me^, and nF_2_ppy^PhOMe/Me^, determined by single-crystal X-ray diffraction. Thermal ellipsoids are shown at 50% probability level with hydrogen atoms, solvent molecules, and counterions omitted for clarity. The structure diagram displays the numbering scheme for the five-membered ring formed by the Ir center and the imino-ADC chelating ligand.

The molecular structures of F_2_ppy^dmp/Me^, F_2_ppy^dipp/Me^, F_2_ppy^dmp/^*^n^*^Pr^, ppz^dmp/Me^, and nF_2_ppy^PhOMe/Me^ were determined by single-crystal X-ray diffraction and are displayed in Fig. 1. Selected bond lengths and angles of the imino-ADC ligand are given in Table 1, and the detailed crystallographic data is reported in Tables S2–S4. Attempts to obtain single crystals for F_2_ppy^PhOMe/Me^ and nF_2_ppy^dmp/Me^ were unsuccessful. All complexes exhibit a distorted octahedral geometry about the Ir center, with the typical *trans* arrangement of the nitrogen donors of the two C^N ligands.^70^ The structures of the chelating imino-ADC ligands and the deprotonated analogue were validated. They form five-membered chelate rings with C(2)–Ir–N(3) bite angles near 76°. The central aromatic rings of the chelating ligands, originating from the isocyanide, are orthogonal to the chelate plane to avoid steric clash. In general, bond lengths and bond angles around the Ir center do not differ significantly as a result of different substituent patterns. The Ir–C(2) ADC bond lengths of all complexes (2.080(4)–2.095(7) Å) are similar to those of some previously reported Ir complexes with chelating ADC ligands (2.039(6)–2.126(4) Å). The N(1)–C(2)–N(2) angles in the ADC donor (112.7(2)°–113.8(6)°) are also close to the corresponding values in the previously reported analogues (113.9(3)°–116.5(4)°).^27^ The loss of the N(1) ADC proton in the deprotonated compound nF_2_ppy^PhOMe/Me^ was confirmed by the single-crystal structure. A slightly longer Ir–C(2) ADC bond distance and smaller N(1)–C(2)–N(2) angle are also observed in that complex, reflecting the change of the carbon donor from L-type to X-type.

**Table 1:** Selected bond lengths and angles from X-ray crystal structures

	Bond length/Å		Bond angles/°	
	Ir–C(2)	Ir–N(3)	C(2)–Ir–N(3)	N(1)–C(2)–N(2)
F_2_ppy^dmp/Me^	2.080(4)	2.102(3)	75.78(13)	112.9(3)
F_2_ppy^dipp/Me^	2.085(3)	2.092(2)	76.29(10)	112.7(2)
F_2_ppy^dmp/^*^n^*^Pr^	2.091(3)	2.096(3)	75.99(12)	112.9(3)
ppz^dmp/Me^	2.095(7)	2.092(6)	76.7(2)	113.8(6)
nF_2_ppy^PhOMe/Me^	2.110(3)	2.093(3)	77.46(11)	110.7(3)

### Cyclic voltammetry

**Fig. 2:**
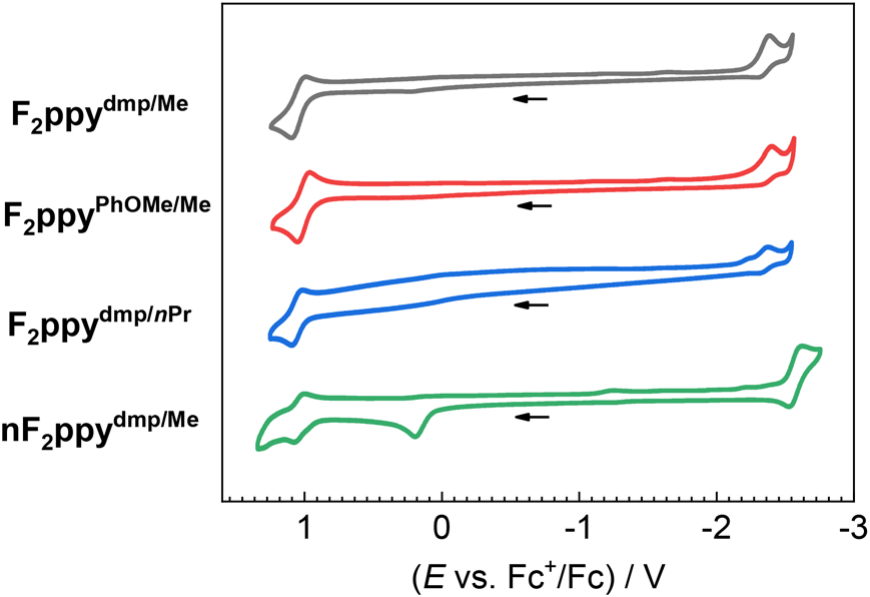
Cyclic voltammograms of complexes F_2_ppy^dmp/Me^, F_2_ppy^PhOMe/Me^, F_2_ppy^dmp/^*^n^*^Pr^, and nF_2_ppy^dmp/Me^ recorded at 0.1 V s^−1^ in acetonitrile with 0.1 M TBAPF_6_ as a supporting electrolyte. Potentials are referenced to an internal standard of ferrocene and currents are normalized.

Cyclic voltammetry was conducted on four representative complexes F_2_ppy^dmp/Me^, F_2_ppy^PhOMe/Me^, F_2_ppy^dmp/^*^n^*^Pr^, and nF_2_ppy^dmp/Me^ to investigate the effect of ancillary ligand substituents and deprotonation on the redox potentials. Fig. 2 shows their voltammograms while Table 2 summarizes their redox potentials. The potentials associated with electrochemical oxidation and reduction are abbreviated as *E*^ox^ and *E*^red^, respectively. Among the cationic complexes, varying the substituent patterns on the ADC aryl ring and the imino alkyl group results in marginal changes in both *E*^ox^ and *E*^red^, suggesting that the imino-ADC ligands are minimally involved in the frontier orbitals. Compared to F_2_ppy^dmp/Me^, its corresponding neutral complex nF_2_ppy^dmp/Me^ gives an additional oxidation peak at 0.19 V, which is consistent with the ancillary ligand becoming more electron rich upon deprotonation. A *ca.* 0.20 V cathodic shift of the reduction potential is also observed in nF_2_ppy^dmp/Me^, indicating destabilization of the LUMO. As such, a smaller electrochemical HOMO–LUMO gap is expected for the neutral complexes, which correlates with a red-shift of the UV-vis absorption bands (*vide infra*).

**Table 2:** Summary of cyclic voltammetry data

Complex	Oxidation *E*^ox^/V	Reduction *E*^red^/V
F_2_ppy^dmp/Me^	1.05	−2.38^b^
F_2_ppy^PhOMe/Me^	1.01	−2.40^b^
F_2_ppy^dmp/*n*Pr^	1.06	−2.37^b^
nF_2_ppy^dmp/Me^	1.04, 0.19^a^	−2.58

a Peak anodic potential (*E*_p,a_) is reported.

b Peak cathodic potentials (*E*_p,c_) are reported.

### Photophysical properties

**Fig. 3:**
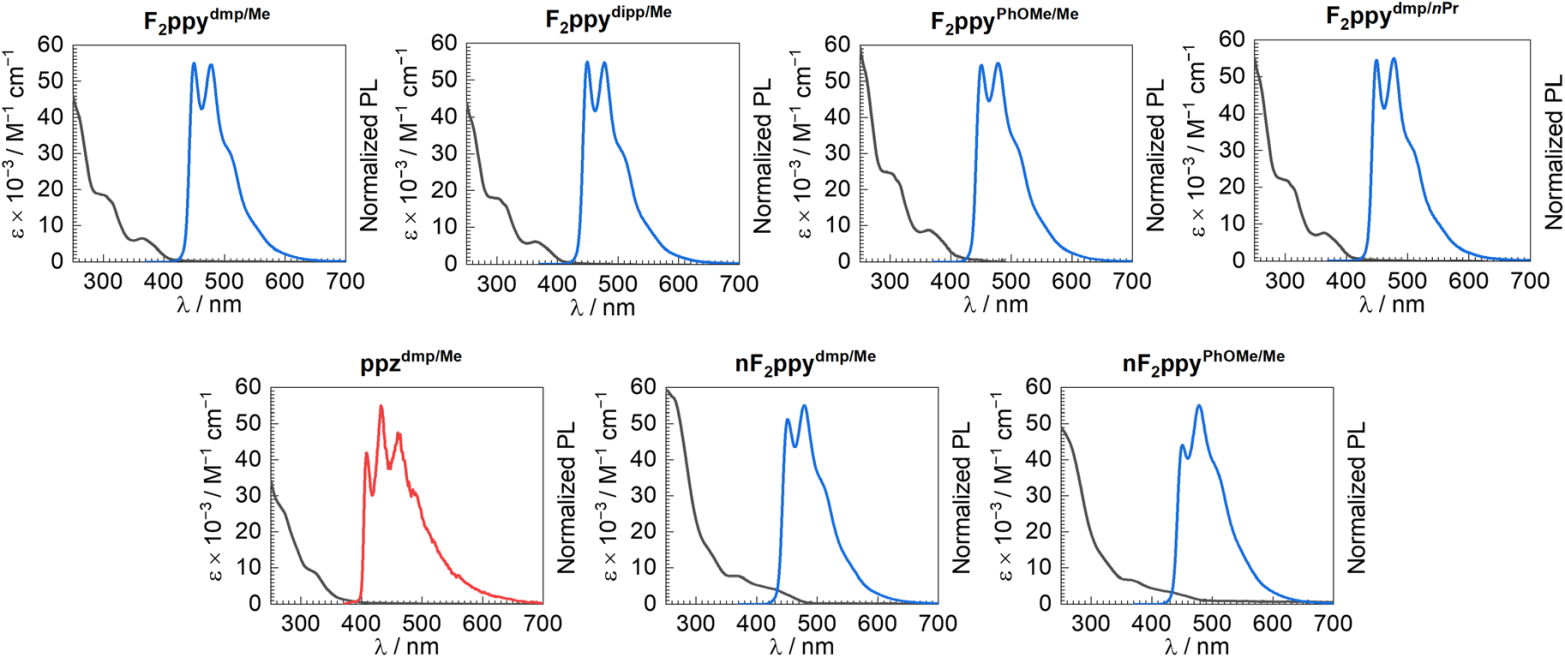
Overlaid UV-vis absorption and normalized photoluminescence (PL) spectra of selected complexes. UV-vis absorption spectra (black lines) were recorded in CH_2_Cl_2_ at room temperature. PL spectra recorded in PMMA films at 2 wt% at room temperature are shown as blue lines. The PL spectrum of ppz^dmp/Me^ was measured in CH_2_Cl_2_ at 77 K and is shown as a red line.

Overlaid UV-vis absorption spectra (recorded in CH_2_Cl_2_ at room temperature) and normalized photoluminescence (PL) spectra (obtained in PMMA film at 2 wt% for the F_2_ppy compounds or in CH_2_Cl_2_ at 77 K for ppz^dmp/Me^) of seven of the complexes are shown in Fig. 3. Photophysical analysis on F_2_ppy^dmp/Ph^ was not conducted, owing to the low material availability and difficulty obtaining a sufficiently pure sample. A summary of the photophysical data is given in Table 3. The UV-vis absorption spectra of F_2_ppy^dmp/Me^, F_2_ppy^dipp/Me^, F_2_ppy^PhOMe/Me^, and F_2_ppy^dmp/^*^n^*^Pr^ show three clear absorption bands with maxima in the UV region. As is typically the case for bis-cyclometalated iridium complexes, the two shorter-wavelength bands likely originate from localized π → π* transitions involving the conjugated ligands, with the longer-wavelength band near 360 nm originating from a metal-to-ligand charge transfer (MLCT) transition.^15,70^ The central band near 320 nm is less pronounced in the absorption spectra of nF_2_ppy^dmp/Me^ and nF_2_ppy^PhOMe/Me^, and there is a clear red-shift of the MLCT band to longer wavelengths, appearing as a shoulder near 410 nm and tailing off near 480 nm. Similar MLCT bands were also observed in previously reported neutral Ir(iii) F_2_ppy complexes containing cyclometalating ADC ligands^27^ or analogous cationic Ir complexes with chelating di(ADC) ligands.^26^ The absorption profile of ppz^dmp/Me^ is generally less well resolved than F_2_ppy^dmp/Me^, with a longer-wavelength shoulder, tailing off before 400 nm, on the side of a more intense UV band.

**Table 3:** Summary of photophysical properties. UV-vis absorption data was measured in CH_2_Cl_2_ solution at room temperature. Photoluminescence data was recorded in PMMA films at 2 wt% unless otherwise noted

Complex	UV-vis absorption	Emission				
	*λ*/nm (*ε* × 10^−3^/M^−1^ cm^−1^)	*λ*/nm	*Φ*_PL_	*τ*/µs	(*k*_r_ × 10^−5^/s^−1^)	(*k*_nr_ × 10^−5^/s^−1^)
F_2_ppy^dmp/Me^	260(40), 306(18), 364(6.4)	450, 479	0.40	2.1	1.9	2.9
F_2_ppy^dipp/Me^	260(38), 305(18), 364(6.0)	449, 477	0.42	1.1	3.8	5.3
F_2_ppy^PhOMe/Me^	260(52), 303(24), 365(8.8)	451, 478	0.30	0.92	3.3	7.6
F_2_ppy^dmp/*n*Pr^	260(48), 304(22), 364(7.5)	449, 478	0.33	2.2	1.5	3.0
ppz^dmp/Me^	270(26), 325(8.4)	408, 432, 460^a^	—	—	—	—
nF_2_ppy^dmp/Me^	265(57), 370(7.8)	451, 479	0.51	1.1	4.6	4.5
nF_2_ppy^PhOMe/Me^	265(45), 370(6.6)	450, 478	0.52	0.76	6.8	6.3

a Recorded in CH_2_Cl_2_ at 77 K.

All seven compounds are very weakly luminescent in CH_2_Cl_2_ solution at room temperature. The six F_2_ppy complexes investigated do phosphoresce in PMMA thin film at room temperature, whereas the ppz compound ppz^dmp/Me^ is nonemissive in PMMA film but is in CH_2_Cl_2_ at 77 K. The PL excitation spectra overlay well with the UV-vis absorption and PL excitation spectra (SI, Fig. S32–S37), confirming that minor impurities do not interfere in the PL emission spectra. PL spectra of the F_2_ppy complexes fall in the sky-blue region, while ppz^dmp/Me^ exhibits a deeper-blue emission (Fig. 3). The PL profiles of these compounds (peak wavelengths and vibronic structure) are in line with those typically observed for previously reported complexes with F_2_ppy and ppz as the cyclometalating ligands, suggesting that the luminescent excited state exclusively involves the C^N ligands, with the new chelating imino-ADC ligand serving as an ancillary ligand.^71–73^ In one previous example with picolinate-supported bis-cyclometalated iridium complexes with F_2_ppy cyclometalating ligands, computed triplet spin density localizes almost exclusively on one of the F_2_ppy cyclometalating ligands, so we presume an analogous localized excited-state is operative in the imino-ADC complexes reported here.^73^ This is also confirmed by PL spectra measured in CH_2_Cl_2_ at 77 K, which are shown for a subset of the imino-ADC complexes overlaid with their respective precursors (SI, Fig. S30 and S31). The PL spectra of the cationic and neutral imino-ADC complexes are nearly identical to those of the chloro-isocyanide precursors, further supporting that the imino-ADC chelating ligand and its deprotonated analogue are not involved in the triplet emissive excited states. The PL spectral features observed here, with well-resolved vibronic structure and little dependence on the ancillary ligand, are somewhat different than our previously reported bis-cyclometalated Ir(iii) complexes bearing F_2_ppy cyclometalating ligands with cyclometalated ADC ancillary ligands. In those compounds, the PL spectra have poorly resolved vibronic structure with maxima that are red-shifted relative to the isocyanide precursors, suggesting that the ADCs impart significant charge-transfer character into the excited state.^27^ Meanwhile, previously reported cationic Ir(iii) F_2_ppy complexes with chelating di(ADC) ligands exhibit similar PL spectra and vibronic structure as those described herein.^26^

The F_2_ppy-supported imino-ADC complexes have moderate photoluminescence quantum yields in PMMA films, with PL lifetimes in the low-microsecond range. The complex F_2_ppy^dmp/Me^ has *Φ*_PL_ = 0.40 and *τ* = 2.1 µs. Increasing the steric bulk of the aryl substituent in F_2_ppy^dipp/Me^ leads to an increase in both the radiative rate constant (*k*_r_) and the nonradiative rate constant (*k*_nr_), which shortens the lifetime (*τ* = 1.1 µs) and results in a nearly identical quantum yield (*Φ*_PL_ = 0.42). Replacing the nitrile-derived methyl group with an *n*-propyl group in F_2_ppy^dmp/^*^n^*^Pr^ has small effects on the decay kinetics and quantum yield (*Φ*_PL_ = 0.33). The larger *k*_nr_ value in F_2_ppy^PhOMe/Me^ results in it having a submicrosecond lifetime (*τ* = 0.92 µs) but with also a slightly lower quantum yield (*Φ*_PL_ = 0.30). Deprotonation of the imino-ADC ancillary ligand has a pronounced effect on the decay rates, leading to a significantly higher *k*_r_ that is responsible for a substantial increase in quantum yield. *Φ*_PL_ increases from 0.40 to 0.51 upon deprotonation of F_2_ppy^dmp/Me^ and from 0.30 to 0.52 when F_2_ppy^PhOMe/Me^ is converted to its neutral form. This increase in *k*_r_ upon deprotonation parallels other works which have shown that electron-rich ancillary ligands can augment radiative rates.^74^ Nevertheless, *Φ*_PL_ values of both the cationic and neutral complexes in this work are lower than those of previously reported cyclometalated Ir(iii) complexes supported by F_2_ppy cyclometalating ligands with cyclometalated ADC ancillary ligands (maximum *Φ*_PL_ = 0.79). Complex ppz^dmp/Me^ only luminesces at 77 K, suggesting that the new imino-ADC chelating ligands are not effective at supporting deep-blue phosphorescence.

## Conclusions

In summary, we have reported the novel “on-complex” organometallic three-component cascade reaction between cyclometalated iridium chloro-isocyanide complexes, nitriles, and propylamine. This reaction gives access to new cyclometalated Ir complexes bearing an imino-ADC chelating ligand that would otherwise be challenging to introduce by traditional means. The ancillary ligand can be deprotonated by sodium methoxide to give corresponding charge-neutral complexes. Photophysical studies show that the products with C^N = F_2_ppy exhibit sky-blue phosphorescence with moderate quantum yields, whereas the C^N = ppz analogue exhibits deeper blue phosphorescence but only at 77 K. Deprotonation of the ancillary ligand gives an additional layer of control over the photophysical properties, increasing the radiative rate and quantum yield while minimally impacting the PL spectral profile. This work represents a fundamental advance in organometallic synthesis, unveiling a new type of multicomponent organometallic cascade reaction that gives access to an unusual imino-ADC chelating ligand and can be further developed in other contexts.

## Author contributions

Son N. T. Phan: investigation, formal analysis, validation, visualization, writing – original draft, writing – review & editing. Vinh Q. Dang: investigation, formal analysis, writing – review & editing. Thomas S. Teets: conceptualization, formal analysis, funding acquisition, project administration, supervision, visualization, writing – review & editing.

## Conflicts of interest

There are no conflicts to declare.

## Supplementary Material

SC-OLF-D6SC00822D-s001

SC-OLF-D6SC00822D-s002

## Data Availability

The datasets supporting this article have been uploaded as part of the supplementary information (SI). Supplementary information: experimental details, X-ray crystallography summary tables, NMR spectra, and additional photophysical data. See DOI: https://doi.org/10.1039/d6sc00822d. CCDC 2518473 (F_2_ppy^dmp/Me^), 2518474 (F_2_ppy^dipp/Me^), 2518475 (F_2_ppy^dmp/Ph^), 2518476 (ppz^dmp/Me^), 2518477 (nF_2_ppy^PhOMe/Me^), 2518478 (F_2_ppy^dmp/^*^n^*^Pr^), and 2518662 (F_2_ppy*^t^*^Bu/NH2Pr^) contain the supplementary crystallographic data for this paper. ^75*a*–*g*^
