# Supplementary material for: On-complex three-component cascade reactions involving phosphorescent cyclometalated Ir(iii) chloro-isocyanide complexes, nitriles, and propylamine
Source: Chem Sci. 2026 Mar 25;17(19):9591–9. doi: 10.1039/d6sc00822d (PMC13015965; doi:10.1039/d6sc00822d)
Supplement: SC-017-D6SC00822D-s001 [file SC-017-D6SC00822D-s001.pdf]

Supplementary Information (SI) for Chemical Science.
This journal is © The Royal Society of Chemistry 2026

Supplementary Information

On-complex three-component cascade reactions involving phosphorescent cyclometalated Ir(III) chloro-isocyanide complexes, nitriles, and propylamine

Son N. T. Phan, Vinh Q. Dang, and Thomas S. Teets*

University of Houston, Department of Chemistry 3585 Cullen Blvd., Room 112, Houston, Texas, 77204-5003, United States.

*Corresponding author: tteets@uh.edu

Index	*Page*
General considerations and experimental details	S2–S10
Summary of unsuccessful substrates	S11
Proposed mechanism for the three-component reaction	S12
X-ray crystallographic summary tables	S13–S15
Molecular structures of **$F_2ppy^{dmp/Ph}$** and **$F_2ppy^{tBu/NH2Pr}$**	S16
NMR spectra of complexes	S17–S30
Overlaid photoluminescence spectra	S31
Overlaid UV–vis absorption and excitation spectra	S32–S34
ESI-MS accurate mass reports of complexes	S35–S40
Supplementary Information References	S41

General considerations

Materials

Commercially available reagents were used without purification unless otherwise noted. Solvents for optical measurements were dried and deoxygenated using a Grubbs solvent purification pressurized with argon. Chloro-bridged cyclometalated iridium dimers were prepared according to previously reported method,[1,2] by refluxing $IrCl_3 \cdot H_2O$ with 2 equiv. of 2-(2,4-difluorophenyl)pyridine (F_2ppy) or 1-phenyl-1H-pyrazole (ppz) in a 1:3 (v/v) mixture of 2-ethoxyethanol and DI water.

Physical methods

^{1}H, ^{13}C{^{1}H}, and ^{19}F NMR spectra were recorded at room temperature using JOEL ECA-400 and ECA-500 spectrometers. ^{1}H and ^{13}C chemical shifts were referenced to the residual solvent resonance: 7.26 ppm for ^{1}H and 77.16 ppm for ^{13}C when using $CDCl_3$, and 2.05 ppm for ^{1}H when using $(CD_3)_2CO$.[3] ^{19}F chemical shifts were referenced to the internal ^{2}H lock signal of the solvent. The ESI-MS experiments were carried out on an Agilent Technologies 6546 accurate-mass Q-TOF LC/MS instrument. UV–vis absorption spectra were measured in CH_2Cl_2 in screw-capped 1 cm quartz cuvettes using an Agilent Cary 8454 UV–vis spectrophotometer. Photoluminescence (PL) spectra were collected using a Horiba FluoroMax-4 spectrofluorometer with a 370 nm long-pass filter to exclude the stray excitation light from detection. Samples for PL spectra were prepared in a nitrogen-filled glovebox using solvents obtained from the Grubbs solvent purification system. For PL measurements at 77 K, the sample was contained in a custom quartz EPR tube with a high-vacuum valve and cooled in liquid nitrogen using a quartz dewar sample holder specifically designed for the fluorimeter's sample chamber. Thin-film poly(methylmethacrylate) (PMMA) samples were prepared inside the nitrogen-filled glovebox at room temperature by drop-coating a quartz slide with a solution of PMMA (98 mg) and respective iridium complex (2.0 mg) dissolved in 1.0 mL of CH_2Cl_2. The absolute quantum yields of complexes doped into PMMA films were measured by using a Spectralon-coated integrating sphere integrated with a Horiba FluoroMax-4 spectrofluorometer. Cyclic voltammetry measurements were conducted with a CH Instrument 602E potentiostat using a three-electrode system, interfaced with a nitrogen glovebox via wire feedthroughs. Measurements were carried out in acetonitrile solution with 0.1 M TBAPF6 as a supporting electrolyte, by using a 3 mm diameter glassy carbon working electrode, Pt wire counter electrode, and silver wire pseudoreference electrode.

X-ray crystallography details

Single crystals were mounted on a Bruker Apex II three-circle diffractometer using Mo Kα radiation (λ = 0.71073 Å). The data was collected at 150 K in most cases, except for **nF$_2$ppy$^{PhOMe/Me}$**, which was measured at 200 K. The data was then processed and refined within the APEXII software. Structures were solved by intrinsic phasing in SHELXT and refined by standard difference Fourier techniques in the program SHELXL.[4] Hydrogen atoms were placed in calculated positions using the standard riding model and refined isotropically; all non-hydrogen atoms anisotropically. The structure of **nF$_2$ppy$^{PhOMe/Me}$** included heavily disordered solvent electron density that could not be satisfactorily refined, requiring the use of the SQUEEZE function in PLATON.[5] Crystallographic details are summarized in Tables S2–S4.

Synthesis of Ir(III) chloro-isocyanide complexes. The chloro-isocyanide precursors were prepared following a modified reported method.[6] General procedure: Inside the glove box, a 100-mL round-bottom flask equipped with a magnetic stir bar was charged with the respective cyclometalated dichloro-bridged iridium dimer, isocyanide, and dichloromethane (CH_2Cl_2). This mixture was stirred at room temperature overnight. Upon completion, CH_2Cl_2 was removed under vacuum, and the mixture was subjected to column chromatography (silica gel, CH_2Cl_2 then ethyl acetate/CH_2Cl_2 1:10 v/v). The desired product was washed with hexane and dried under vacuum.

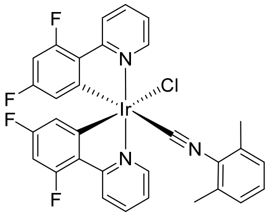

F_2ppy$^{dmp/Cl}$

F_2ppy$^{dmp/Cl}$: Prepared following the general procedure using [Ir(F_2ppy)$_2$(μ-Cl)]$_2$ (0.50 mmol, 0.61 g), 2,6-dimethylphenyl isocyanide (1.5 mmol, 0.20 g), and CH_2Cl_2 (50 mL). The product was obtained as a yellow solid. Yield: 0.33 g, 45%. ^{1}H NMR (500 MHz, $CDCl_3$) δ 9.99 (d, *J* = 5.7 Hz, 1H, Ar*H*), 9.22 (d, *J* = 5.8 Hz, 1H, Ar*H*), 8.35 (d, *J* = 8.3 Hz, 1H, Ar*H*), 8.29 (d, *J* = 8.4 Hz, 1H, Ar*H*), 7.90 (dt, *J* = 12.8, 7.8 Hz, 2H, Ar*H*), 7.33 (ddd, *J* = 7.5, 6.1, 1.5 Hz, 1H, Ar*H*), 7.20 (ddd, *J* = 7.3, 6.0, 1.5 Hz, 1H, Ar*H*), 7.15 (t, *J* = 7.7 Hz, 1H, Ar*H*), 7.04 (d, *J* = 7.7 Hz, 2H, Ar*H*), 6.47–6.35 (m, 2H, Ar*H*), 5.86 (dd, *J* = 8.5, 2.4 Hz, 1H, Ar*H*), 5.59 (dd, *J* = 8.2, 2.3 Hz, 1H, Ar*H*), 2.1 (s, 6H, C*H*$_3$). This compound is known.[6]

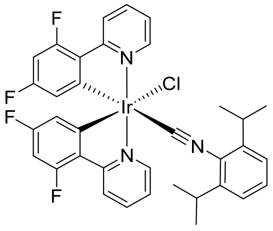

F_2ppy$^{dipp/Cl}$

F_2ppy$^{dipp/Cl}$: Prepared following the general procedure using [Ir(F_2ppy)$_2$(μ-Cl)]$_2$ (0.40 mmol, 0.49 g), 2,6-diisopropylphenyl isocyanide (1.2 mmol, 0.23 g; synthesized following a reported procedure[7]), and CH_2Cl_2 (40 mL). The product was obtained as a yellow solid. Yield: 0.22 g, 35%. ^{1}H NMR (500 MHz, $CDCl_3$) δ 10.01 (d, *J* = 5.8 Hz, 1H, Ar*H*), 9.23 (d, *J* = 5.8 Hz, 1H, Ar*H*), 8.35 (d, *J* = 8.8 Hz, 1H, Ar*H*), 8.29 (d, *J* = 8.4 Hz, 1H, Ar*H*), 7.90 (dt, *J* = 14.6, 8.0 Hz, 2H, Ar*H*), 7.35–7.24 (m, 2H, Ar*H*), 7.18 (ddd, *J* = 7.3, 5.8, 1.4 Hz, 1H, Ar*H*), 7.09 (d, *J* = 7.8 Hz, 2H, Ar*H*), 6.47–6.36 (m, 2H, Ar*H*), 5.88 (dd, *J* = 8.5, 2.3 Hz, 1H, Ar*H*), 5.58 (dd, *J* = 8.1, 2.3 Hz, 1H, Ar*H*), 2.94 (sept, *J* = 7.1 Hz, 2H, C*H*(CH_3)$_2$), 1.07 (d, *J* = 7.0 Hz, 6H, CH(C*H*$_3$)$_2$), 1.06 (d, *J* = 7.0 Hz, 6H, CH(C*H*$_3$)$_2$). ^{19}F NMR (470 MHz, $CDCl_3$) δ −107.22 to −107.35 (m, 2F), −109.50 (t, *J* = 11.7 Hz, 1F), −110.03 (t, *J* = 11.4 Hz, 1F). HRMS-ESI: (m/z): $[M-Cl]^+$ calcd for $C_{35}H_{29}ClF_4IrN_3$, 758.1892; found, 758.1885.

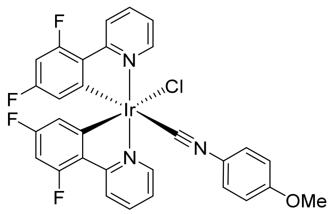

$F_2ppy^{PhOMe/Cl}$

$F_2ppy^{PhOMe/Cl}$: Prepared following the general procedure using $[Ir(F_2ppy)_2(\mu\text{-}Cl)]_2$ (0.50 mmol, 0.61 g), 4-methoxyphenyl isocyanide (1.5 mmol, 0.20 g), and CH_2Cl_2 (50 mL). The product was obtained as a yellow solid. Yield: 0.39 g, 48%. ^{1}H NMR (500 MHz, $CDCl_3$) δ 9.95–9.92 (m, 1H, Ar*H*), 9.17 (ddd, *J* = 5.8, 1.7, 0.7 Hz, 1H, Ar*H*), 8.36–8.31 (m, 1H, Ar*H*), 8.29 (d, *J* = 8.6 Hz, 1H, Ar*H*), 7.9 (dddd, *J* = 15.2, 8.7, 7.6, 1.6 Hz, 2H, Ar*H*), 7.32 (ddd, *J* = 7.3, 5.8, 1.4 Hz, 1H, Ar*H*), 7.25–7.18 (m, 3H, Ar*H*), 6.97–6.76 (m, 2H, Ar*H*), 6.47–6.34 (m, 2H, Ar*H*), 5.84 (dd, *J* = 8.5, 2.4 Hz, 1H, Ar*H*), 5.51 (dd, *J* = 8.2, 2.4 Hz, 1H, Ar*H*), 3.80 (s, 3H, OCH_3). This compound is known.[8]

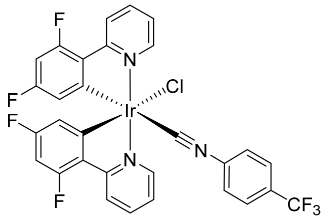

$F_2ppy^{PhCF3/Cl}$

$F_2ppy^{PhCF3/Cl}$: Prepared following the general procedure using $[Ir(F_2ppy)_2(\mu\text{-}Cl)]_2$ (0.40 mmol, 0.49 g), 4-(trifluoromethyl)phenyl isocyanide (1.2 mmol, 0.21 g; synthesized following a reported procedure[9]), and CH_2Cl_2 (40 mL). The product was obtained as a yellow solid. Yield: 0.21 g, 34%. ^{1}H NMR (500 MHz, $CDCl_3$) δ 9.93 (d, *J* = 5.8 Hz, 1H, Ar*H*), 9.14 (d, *J* = 5.8 Hz, 1H, Ar*H*), 8.40–8.31 (m, 1H, Ar*H*), 8.32 (d, *J* = 8.4 Hz, 1H, Ar*H*), 7.92 (dt, *J* = 16.2, 7.9 Hz, 2H, Ar*H*), 7.65 (d, *J* = 8.3 Hz, 2H, Ar*H*), 7.44 (d, *J* = 8.3 Hz, 2H, Ar*H*), 7.3 (ddd, *J* = 7.3, 5.9, 1.4 Hz, 1H, Ar*H*), 7.21 (ddd, *J* = 7.5, 5.8, 1.4 Hz, 1H, Ar*H*), 6.46 (ddd, *J* = 11.7, 9.1, 2.3 Hz, 1H, Ar*H*), 6.40 (ddd, *J* = 12.0, 9.1, 2.4 Hz, 1H, Ar*H*), 5.85 (dd, *J* = 8.5, 2.3 Hz, 1H, Ar*H*), 5.49 (dd, *J* = 8.1, 2.3 Hz, 1H, Ar*H*). This compound is known.[1]

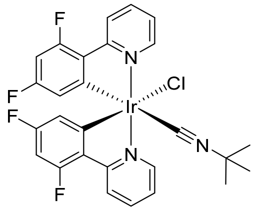

$F_2ppy^{tBu/Cl}$

$F_2ppy^{tBu/Cl}$: Prepared following the general procedure using $[Ir(F_2ppy)_2(\mu\text{-}Cl)]_2$ (0.40 mmol, 0.49 g), *tert*-butyl isocyanide (1.2 mmol, 0.10 g), and CH_2Cl_2 (40 mL). The product was obtained as an orange solid. Yield: 0.23 g, 42%. ^{1}H NMR (500 MHz, $CDCl_3$) δ 9.89 (dd, *J* = 6.0, 1.6 Hz, 1H, Ar*H*), 9.03 (dd, *J* = 5.8, 1.6 Hz, 1H, Ar*H*), 8.34–8.29 (m, 1H, Ar*H*), 8.27 (d, *J* = 8.4 Hz, 1H), 7.87 (dt, *J* = 16.2, 8.3 Hz, 2H, Ar*H*), 7.30 (ddd, *J* = 7.3, 5.9, 1.4 Hz, 1H, Ar*H*), 7.18 (ddd, *J* = 7.4, 5.8, 1.4 Hz, 1H, Ar*H*), 6.40 (ddd, *J* = 12.0, 9.2, 2.3 Hz, 1H), 6.35 (ddd, *J* = 12.2, 9.2, 2.4 Hz, 1H, Ar*H*), 5.77 (dd, *J* = 8.6, 2.4 Hz, 1H, Ar*H*), 5.52 (dd, *J* = 8.2, 2.3 Hz, 1H, Ar*H*), 1.38 (s, 9H, C(CH_3)$_3$). This compound is known.[6]

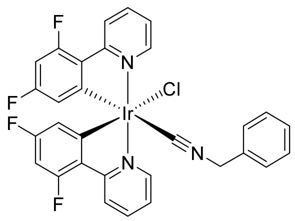

$F_2ppy^{Bn/Cl}$

$F_2ppy^{Bn/Cl}$: Prepared following the general procedure using $[Ir(F_2ppy)_2(\mu\text{-}Cl)]_2$ (0.40 mmol, 0.49 g), benzyl isocyanide (1.2 mmol, 0.14 g), and CH_2Cl_2 (40 mL). The product was obtained as a yellow solid. Yield: 0.27 g, 47%. ^1H NMR (500 MHz, $CDCl_3$) δ 9.91 (dd, *J* = 5.9, 1.6 Hz, 1H, Ar*H*), 8.95 (d, *J* = 5.7 Hz, 1H, Ar*H*), 8.33–8.28 (m, 1H, Ar*H*), 8.27 (d, *J* = 8.3 Hz, 1H, Ar*H*), 7.89 (t, *J* = 7.6 Hz, 1H, Ar*H*), 7.83 (t, *J* = 7.9 Hz, 1H, Ar*H*), 7.38–7.28 (m, 4H, Ar*H*), 7.10 (dd, *J* = 7.2, 2.1 Hz, 2H, Ar*H*), 7.05 (ddd, *J* = 7.4, 5.8, 1.4 Hz, 1H, Ar*H*), 6.43 (ddd, *J* = 12.1, 9.2, 2.3 Hz, 1H, Ar*H*), 6.36 (ddd, *J* = 12.2, 9.2, 2.4 Hz, 1H, Ar*H*), 5.78 (dd, *J* = 8.5, 2.4 Hz, 1H, Ar*H*), 5.51 (dd, *J* = 8.2, 2.3 Hz, 1H, Ar*H*), 4.82 (d, *J* = 2.7 Hz, 2H, NC*H*$_2$). ^{19}F NMR (470 MHz, $CDCl_3$) δ −107.37 to −107.53 (m, 2F), −109.68 (ddd, *J* = 13.1, 10.4, 3.0 Hz, 1F), −109.98 (t, *J* = 11.2 Hz, 1F). HRMS-ESI: (m/z): $[M-Cl]^+$ calcd for $C_{30}H_{19}ClF_4IrN_3$, 688.1107; found, 688.1101.

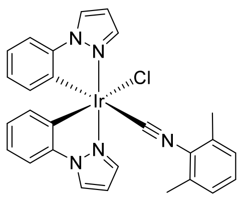

$ppz^{dmp/Cl}$

$ppz^{dmp/Cl}$: Prepared following the general procedure using $[Ir(ppz)_2(\mu\text{-}Cl)]_2$ (0.25 mmol, 0.31 g), 2,6-dimethylphenyl isocyanide (0.75 mmol, 0.10 g), and CH_2Cl_2 (30 mL). The product was obtained as a gray solid. Yield: 0.20 g, 61%. ^1H NMR (500 MHz, $CDCl_3$) δ 8.46 (s, 1H, Ar*H*), 8.13 (d, *J* = 2.9 Hz, 1H, Ar*H*), 8.05 (dd, *J* = 10.7, 2.6 Hz, 2H, Ar*H*), 7.19 (d, *J* = 7.9 Hz, 2H, Ar*H*), 7.12 (t, *J* = 7.6 Hz, 1H, Ar*H*), 7.02 (d, *J* = 7.5 Hz, 2H, Ar*H*), 6.94–6.85 (m, 2H, Ar*H*), 6.80 (t, *J* = 7.3 Hz, 1H, Ar*H*), 6.75 (t, *J* = 2.4 Hz, 1H, Ar*H*), 6.69 (t, *J* = 7.6 Hz, 1H, Ar*H*), 6.63 (t, *J* = 2.6 Hz, 1H, Ar*H*), 6.37 (d, *J* = 7.6 Hz, 1H, Ar*H*), 6.26 (d, *J* = 7.4 Hz, 1H, Ar*H*), 2.19 (s, 6H, C*H*$_3$). $^{13}C\{^1H\}$ NMR (126 MHz, $CDCl_3$) δ 147.3, 142.8, 142.1, 140.5, 139.3, 135.3, 133.2, 132.7, 128.7, 127.9, 126.7, 126.2, 126.1, 125.9, 125.6, 123.3, 122.4, 111.3, 110.9, 108.3, 107.7, 18.7. HRMS-ESI: (m/z): $[M-Cl]^+$ calcd for $C_{27}H_{23}ClIrN_5$, 608.1547; found, 608.1540.

Synthesis of cationic Ir(III) complexes via 3-component reaction. General procedure: A 20-mL vial equipped with a magnetic stir bar was charged with the respective chloro-isocyanide precursor, propylamine, and nitrile. This mixture was stirred at room temperature or at 60 °C overnight. Upon completion, the solvent was removed under vacuum. The reaction vial was then transferred to a glove box, to which was added a saturated solution of NH_4PF_6 in MeOH, prepared by dissolving 100 mg of NH_4PF_6 in 3 mL of MeOH. The mixture was stirred for another 4 hours. After that, the solvent was removed under vacuum. The mixture was redissolved in CH_2Cl_2 and filtered to remove excess NH_4PF_6. The product was isolated by column chromatography, recrystallization, or a combination of both methods.

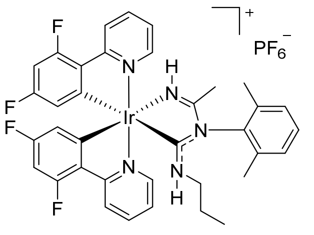

F$_2$ppy$^{dmp/Me}$

F$_2$ppy$^{dmp/Me}$: Prepared following the general procedure. When the reaction was conducted at room temperature, **F$_2$ppy$^{dmp/Cl}$** (0.24 mmol, 0.18 g), propylamine (excess, 1.0 mL), and CH_3CN (excess, 5.0 mL) were used. The product was isolated by column chromatography (silica gel, CH_2Cl_2 then ethyl acetate/CH_2Cl_2 1:10 v/v), followed by recrystallization from CH_2Cl_2/pentane to give a light yellow solid. Yield: 50 mg, 22%. When the reaction was conducted at 60 °C, **F$_2$ppy$^{dmp/Cl}$** (0.13 mmol, 0.10 g), propylamine (excess, 0.5 mL), and CH_3CN (excess, 3.0 mL) were used. The product was isolated by recrystallization from CH_2Cl_2/pentane. Yield: 91 mg, 71%. ^{1}H NMR (500 MHz, $CDCl_3$) δ 8.57 (dd, *J* = 5.9, 1.6 Hz, 1H, Ar*H*), 8.48 (dd, *J* = 5.8, 1.6 Hz, 1H, Ar*H*), 8.38–8.30 (m, 2H, Ar*H*), 8.28 (s, 1H, N*H*), 7.88 (dtd, *J* = 13.2, 7.7, 1.5 Hz, 2H, Ar*H*), 7.43–7.34 (m, 2H, Ar*H*), 7.32 (d, *J* = 7.5 Hz, 1H, Ar*H*), 7.25–7.19 (m, 2H, Ar*H*), 6.92 (t, *J* = 6.4 Hz, 1H, N*H*), 6.41 (ddd, *J* = 12.5, 8.9, 2.3 Hz, 2H, Ar*H*), 5.74 (dd, *J* = 8.7, 2.4 Hz, 1H, Ar*H*), 5.49 (dd, *J* = 8.0, 2.3 Hz, 1H, Ar*H*), 3.16–2.85 (m, 2H, NCH_2), 2.17 (s, 3H, CH_3), 2.16 (s, 3H, CH_3), 1.80 (s, 3H, CH_3), 1.10–0.95 (m, 1H, CH_2CH$_3$), 0.5–0.60 (m, 1H, CH_2CH$_3$), 0.31 (t, *J* = 7.4 Hz, 3H, CH$_2$CH_3). ^{19}F NMR (470 MHz, $CDCl_3$) δ −72.45 (d, $J_{F–P}$ = 713 Hz, 6F, PF_6), −105.40 to −105.67 (m, 1F), −106.52 (dt, *J* = 10.8, 8.8 Hz, 1F), −108.78 to −109.18 (m, 2F). HRMS-ESI: (m/z): $[M-PF_6]^+$ calcd for $C_{36}H_{33}F_{10}IrN_5P$, 802.2263; found, 802.2257.

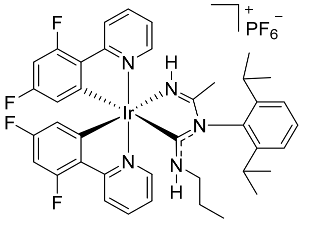

F$_2$ppy$^{dipp/Me}$

F$_2$ppy$^{dipp/Me}$: Prepared following the general procedure. When the reaction was conducted at room temperature, **F$_2$ppy$^{dipp/Cl}$** (0.23 mmol, 0.18 g), propylamine (excess, 1.0 mL), and CH_3CN (excess, 5.0 mL) were used. The product was isolated by column chromatography (silica gel, CH_2Cl_2 then ethyl acetate/CH_2Cl_2 1:10 v/v), followed by recrystallization from CH_2Cl_2/pentane to give a light yellow solid. Yield: 48 mg, 20%. When the reaction was conducted at 60 °C, **F$_2$ppy$^{dipp/Cl}$** (0.13 mmol, 0.10 g), propylamine (excess, 0.5 mL), and CH_3CN (excess, 3.0 mL) were used. The product was isolated by column chromatography (silica gel, CH_2Cl_2 then ethyl acetate/CH_2Cl_2 1:10 v/v). Yield: 68 mg, 54%. ^{1}H NMR (500 MHz, $(CD_3)_2CO$) δ 10.00 (s, 1H, N*H*), 8.88 (d, *J* = 5.8 Hz, 1H, Ar*H*), 8.73 (d, *J* = 5.8 Hz, 1H, Ar*H*), 8.50–8.43 (m, 3H, Ar*H*), 8.23 (td, *J* = 7.9, 3.7 Hz, 2H, Ar*H*), 7.64 (q, *J* = 7.6 Hz, 2H, Ar*H*), 7.59–7.53 (m, 2H, Ar*H*), 7.47 (d, *J* = 7.7 Hz, 1H, Ar*H*), 6.64 (ddt, *J* = 12.7, 9.2, 3.1 Hz, 2H, Ar*H*), 5.90 (dd, *J* = 9.0, 2.3 Hz, 1H, Ar*H*), 5.62 (dd, *J* = 8.0, 2.4 Hz, 1H, Ar*H*), 3.39–3.30 (m, 1H, NCH_2), 3.16–3.07 (m, 1H, NCH_2), 2.82–2.78 (m, 2H, CH_2CH$_3$), 2.50 (sept, *J* = 6.9 Hz, 1H, C*H*(CH$_3$)$_2$), 2.29 (s, 3H, CH_3), 1.45 (d, *J* = 6.8 Hz, 3H, CH(CH_3)$_2$), 1.28 (d, *J* = 7.2 Hz, 3H, CH(CH_3)$_2$), 1.27 (d, *J* = 6.6 Hz, 3H, CH(CH_3)$_2$), 0.79 (d, *J* = 6.8 Hz, 3H, CH(CH_3)$_2$), 0.75–0.62 (m, 1H, C*H*(CH$_3$)$_2$), 0.26 (t, *J* = 7.3 Hz, 3H, CH$_2$CH_3). One of the N*H* peaks overlaps with an aromatic peak. ^{19}F NMR (470 MHz, $(CD_3)_2CO$) δ −72.50 (d, $J_{F–P}$ = 708 Hz, 6F, PF_6),

−108.65 (q, J = 9.6 Hz, 1F), −108.86 (q, J = 9.2 Hz, 1F), −110.32 (t, J = 11.7 Hz, 1F), −110.66 (ddd, J = 12.6, 9.9, 2.6 Hz, 1F). HRMS-ESI: (m/z): $[M-PF_6]^+$ calcd for $C_{40}H_{41}F_{10}IrN_5P$, 858.2891; found, 858.2886.

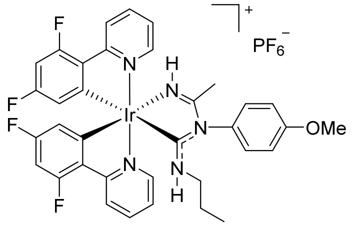

F₂ppy$^{PhOMe/Me}$

F_2ppy$^{PhOMe/Me}$: Prepared following the general procedure. The reaction was conducted at room temperature using **F_2ppy$^{PhOMe/Cl}$** (0.24 mmol, 0.18 g), propylamine (excess, 1.0 mL), and CH_3CN (excess, 5.0 mL). The product was isolated by column chromatography (silica gel, CH_2Cl_2 then ethyl acetate/CH_2Cl_2 1:10 v/v), followed by recrystallization from CH_2Cl_2/pentane to give a light-yellow solid. Yield: 71 mg, 31%. ^{1}H NMR (400 MHz, $(CD_3)_2CO$) δ 9.80 (s, 1H, NH), 8.83–8.76 (m, 2H, ArH), 8.44 (dt, J = 7.9, 1.7 Hz, 1H, ArH), 8.39 (dt, J = 8.8, 1.9 Hz, 1H, ArH), 8.34 (s, 1H, NH), 8.20–8.11 (m, 2H, ArH), 7.59 (dd, J = 8.7, 2.7 Hz, 1H, ArH), 7.51 (dddd, J = 7.4, 5.8, 3.5, 1.4 Hz, 2H, ArH), 7.28 (dd, J = 8.7, 2.7 Hz, 1H, ArH), 7.19 (dd, J = 8.7, 2.9 Hz, 1H, ArH), 7.14 (dd, J = 8.7, 2.9 Hz, 1H, ArH), 6.57 (ddt, J = 12.8, 9.3, 2.3 Hz, 2H, ArH), 5.85 (dd, J = 8.9, 2.4 Hz, 1H, ArH), 5.56 (dd, J = 8.1, 2.4 Hz, 1H, ArH), 3.88 (s, 3H, OCH_3), 3.21–3.10 (m, 1H, NCH_2), 3.08–2.98 (m, 1H, NCH_2), 2.32 (s, 3H, CH_3), 1.25–1.10 (m, 1H, CH_2CH$_3$), 0.83–0.70 (m, 1H, CH_2CH$_3$), 0.28 (t, J = 7.4 Hz, 3H, CH$_2$CH_3). ^{19}F NMR (470 MHz, $(CD_3)_2CO$) δ −72.38 (d, J_{F-P} = 708 Hz, 6F, PF_6), −109.02 (q, J = 9.5 Hz, 1F), −109.18 (td, J = 9.5, 8.0 Hz, 1F), −110.65 (ddd, J = 12.7, 10.2, 2.3 Hz, 1F), −110.90 (ddd, J = 12.7, 10.0, 2.6 Hz, 1F). HRMS-ESI: (m/z): $[M-PF_6]^+$ calcd for $C_{35}H_{31}F_{10}IrN_5OP$, 804.2055; found, 804.2050.

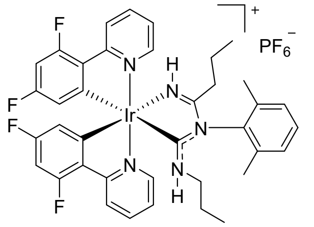

F_2ppy$^{dmp/nPr}$

F_2ppy$^{dmp/nPr}$: Prepared following the general procedure. The reaction was conducted at 60 °C using **F_2ppy$^{dmp/Cl}$** (0.13 mmol, 0.10 g), propylamine (excess, 0.5 mL), and butyronitrile (excess, 3.0 mL). The product was isolated by column chromatography (silica gel, CH_2Cl_2 then ethyl acetate/CH_2Cl_2 1:10 v/v) to give a light-yellow solid. Yield: 68 mg, 54%. ^{1}H NMR (500 MHz, $CDCl_3$) δ 8.50 (dd, J = 5.8, 1.6 Hz, 1H, ArH), 8.46 (dd, J = 5.9, 1.5 Hz, 1H, ArH), 8.37–8.32 (m, 2H, ArH), 8.06 (s, 1H, NH), 7.93–7.86 (m, 2H, ArH), 7.42 (ddd, J = 7.4, 5.8, 1.4 Hz, 1H, ArH), 7.38 (d, J = 7.5 Hz, 1H, ArH), 7.35 (d, J = 7.6 Hz, 1H, ArH), 7.30–7.26 (m, 1H, ArH), 7.23 (d, J = 7.3 Hz, 1H, ArH), 6.98 (t, J = 6.4 Hz, 1H, NH), 6.49–6.39 (m, 2H, ArH), 5.74 (dd, J = 8.7, 2.4 Hz, 1H, ArH), 5.49 (dd, J = 7.9, 2.4 Hz, 1H, ArH), 3.08 (q, J = 7.1 Hz, 2H, NCH_2), 2.44 (ddd, J = 17.7, 9.8, 5.2 Hz, 1H, CCH_2), 2.19 (s, 3H, CCH_3), 2.08 (ddd, J = 17.6, 10.2, 6.0 Hz, 1H, CCH_2), 1.82 (s, 3H, CH_3), 1.73–1.61 (m, 1H, CH_2CH$_3$), 1.54–1.45 (m, 1H, CH_2CH$_3$), 1.13–0.95 (m, 1H, CH_2CH$_3$), 0.77 (t, J = 7.3 Hz, 3H, CH$_2$CH_3), 0.72–0.61 (m, 1H, CH_2CH$_3$), 0.30 (t, J = 7.4 Hz, 3H, CH$_2$CH_3). ^{19}F NMR (470 MHz, $CDCl_3$) δ −70.09 (d, J_{F-P} = 713 Hz, 6F, PF_6), −105.76 (dt, J = 10.8, 8.4 Hz, 1F), −106.19 (dt, J = 10.9, 8.8 Hz, 1F), −108.78 to −108.86 (m, 1F), −108.95 to −109.08 (m, 1F). HRMS-ESI: (m/z): $[M-PF_6]^+$ calcd for $C_{38}H_{37}F_{10}IrN_5P$, 830.2584; found, 830.2578.

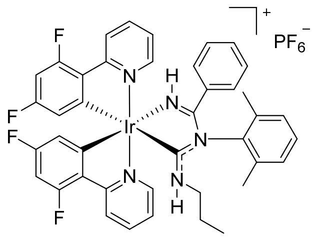

F_2ppy$^{dmp/Ph}$

F_2ppy$^{dmp/Ph}$: Prepared following the general procedure. The reaction was conducted at room temperature using **F_2ppy**$^{dmp/Cl}$ (0.24 mmol, 0.18 g), propylamine (excess, 1.0 mL), and C_6H_5CN (excess, 5.0 mL). The product was isolated by column chromatography (silica gel, CH_2Cl_2 then ethyl acetate/CH_2Cl_2 1:10 v/v), followed by recrystallization from CH_2Cl_2/pentane to give a light-yellow solid. Yield: 4.5 mg, 2.0%. ^{1}H NMR (500 MHz, $CDCl_3$) δ 8.67 (d, *J* = 5.8 Hz, 1H, Ar*H*), 8.50–8.32 (m, 3H, Ar*H*), 8.15 (s, 1H, N*H*), 7.97 (q, *J* = 7.3 Hz, 2H, Ar*H*), 7.48–7.39 (m, 2H, Ar*H*), 7.39–7.31 (m, 2H, Ar*H*), 7.28–7.23 (m, 2H, Ar*H*, overlapped with solvent peak), 7.13 (d, *J* = 7.7 Hz, 2H, Ar*H*), 7.09 (d, *J* = 7.3 Hz, 2H, Ar*H*), 6.47 (ddt, *J* = 12.0, 8.8, 2.6 Hz, 2H, Ar*H*), 5.76 (dd, *J* = 8.7, 2.3 Hz, 1H, Ar*H*), 5.53 (dd, *J* = 7.8, 2.3 Hz, 1H, Ar*H*), 3.28 (q, *J* = 7.4 Hz, 2H, NCH_2), 2.06 (s, 3H, CH_3), 1.90 (s, 3H, CH_3), 1.11 (dq, *J* = 14.5, 7.4 Hz, 1H, CH_2CH_3), 0.67 (dq, *J* = 14.6, 7.4 Hz, 1H, CH_2CH_3), 0.33 (t, *J* = 7.3 Hz, 3H, CH_2CH_3). One of the N*H* peaks overlaps with an aromatic peak. ^{19}F NMR (470 MHz, $CDCl_3$) δ −73.24 (d, $J_{F–P}$ = 713 Hz, 6F, PF_6), −105.17 to −105.27 (m, 1F), −106.19 to −106.32 (m, 1F), −108.69 (t, *J* = 11.7 Hz, 1F), −108.86 (t, *J* = 11.9 Hz, 1F). HRMS-ESI: (m/z): $[M−PF_6]^+$ calcd for $C_{41}H_{35}F_{10}IrN_5P$, 864.2419; found, 864.2413.

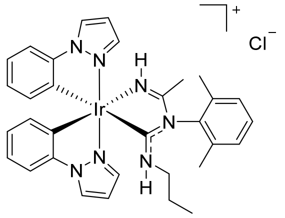

ppz$^{dmp/Me}$

ppz$^{dmp/Me}$: A 20-mL vial equipped with a magnetic stir bar was charged with **ppz**$^{dmp/Cl}$ (0.28 mmol, 0.18 g), propylamine (excess, 1.0 mL), and CH_3CN (excess, 5.0 mL). This mixture was stirred at room temperature overnight. Upon completion, the solvent was removed under vacuum. The product was obtained by recrystallization from CH_2Cl_2/pentane to give a brownish gray solid. Yield: 47 mg, 41%. ^{1}H NMR (400 MHz, $CDCl_3$) δ 9.58 (s, 1H, N*H*), 8.11 (d, *J* = 2.8 Hz, 2H, Ar*H*), 7.99 (d, *J* = 2.2 Hz, 1H, Ar*H*), 7.53 (d, *J* = 2.2 Hz, 1H, Ar*H*), 7.40–7.27 (m, 3H, Ar*H*), 7.25–7.14 (m, 3H, Ar*H*), 6.92 (td, *J* = 7.6, 1.3 Hz, 1H, Ar*H*), 6.84 (td, *J* = 7.6, 1.3 Hz, 1H, Ar*H*), 6.79 (td, *J* = 7.4, 1.0 Hz, 1H, Ar*H*), 6.72–6.65 (m, 2H, Ar*H*), 6.63 (t, *J* = 2.5 Hz, 1H, Ar*H*), 6.24 (dd, *J* = 7.3, 1.3 Hz, 1H, Ar*H*), 6.20 (dd, *J* = 7.5, 1.3 Hz, 1H, Ar*H*), 3.32–3.20 (m, 1H, NCH_2), 3.12–3.01 (m, 1H, NCH_2), 2.29 (s, 3H, CH_3), 2.20 (s, 3H, CH_3), 1.92 (s, 3H, CH_3), 1.11–0.96 (m, 1H, CH_2CH_3), 0.78–0.65 (m, 1H, CH_2CH_3), 0.23 (t, *J* = 7.3 Hz, 3H, CH_2CH_3). One of the N*H* peaks overlaps with an aromatic peak. ^{13}C{^{1}H} NMR (101 MHz, $CDCl_3$) δ 201.4, 172.0, 142.5, 142.4, 140.2, 139.2, 136.5, 136.43, 136.36, 133.5, 133.0, 131.3, 130.6, 130.5, 126.9, 126.6, 126.5, 125.6, 123.3, 121.7, 111.4, 111.3, 108.7, 108.3, 50.4, 24.7, 21.1, 18.2, 18.1, 10.3. HRMS-ESI: (m/z): $[M−Cl]^+$ calcd for $C_{32}H_{35}IrN_7Cl$, 708.2544; found, 708.2538.

Deprotonation of the cationic Ir(III) complexes. General procedure: A 20-mL vial equipped with a magnetic stir bar was charged with the respective cationic Ir(III) complexes, sodium methoxide (NaOMe), and methanol (MeOH). This mixture was stirred at room temperature overnight. Upon completion, the solvent was removed under vacuum. The mixture was redissolved in CH_2Cl_2 and filtered to remove sodium salts. The crude product was dissolved in a minimum amount of CH_2Cl_2. An excess amount of hexane was then added to precipitate the product out of the mixture, which was then filtered and dried under vacuum.

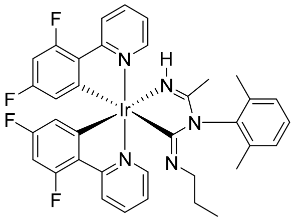

nF$_2$ppy$^{dmp/Me}$

nF$_2$ppy$^{dmp/Me}$: Prepared following the general procedure using **F$_2$ppy$^{dmp/Me}$** (0.016 mmol, 15 mg), NaOMe (4.0 equiv., 0.064 mmol, 3.5 mg), and MeOH (3.0 mL). The product was obtained as a yellow solid. Yield: 10 mg, 81%. ^{1}H NMR (500 MHz, $CDCl_3$) δ 9.04 (s, 1H, Ar*H*), 8.58 (d, *J* = 5.5 Hz, 1H, Ar*H*), 8.32–8.27 (m, 2H, Ar*H*), 7.78 (td, *J* = 7.6, 1.5 Hz, 1H, Ar*H*), 7.75–7.69 (m, 1H, Ar*H*), 7.14–6.98 (m, 5H, Ar*H*), 6.36 (ddd, *J* = 12.7, 9.1, 2.4 Hz, 1H, Ar*H*), 6.30 (ddd, *J* = 12.9, 9.3, 2.4 Hz, 1H, Ar*H*), 5.86 (dd, *J* = 9.3, 2.4 Hz, 1H, Ar*H*), 5.69 (dd, *J* = 7.6, 2.4 Hz, 1H, Ar*H*), 2.86 (td, *J* = 10.3, 5.3 Hz, 1H, NC*H*$_2$), 2.74 (td, *J* = 10.2, 5.1 Hz, 1H, NC*H*$_2$), 2.09 (s, 3H, C*H*$_3$), 1.89 (s, 3H, C*H*$_3$), 1.81 (s, 3H, CC*H*$_3$), 0.93 (dddd, *J* = 12.5, 10.2, 7.4, 5.4 Hz, 1H, C*H*$_2$CH$_3$), 0.38–0.28 (m, 1H, C*H*$_2$CH$_3$), 0.24 (t, *J* = 7.2 Hz, 3H, CH$_2$C*H*$_3$). The N*H* peak was not located in the spectrum. ^{19}F NMR (470 MHz, $CDCl_3$) δ −109.16 (br, s, 1F), −110.23 (br, s, 1F), −110.58 (br, s, 1F), −111.36 (br, s, 1F). HRMS-ESI: (m/z): $[M+H]^+$ calcd for $C_{36}H_{32}F_4IrN_5$, 802.2278; found, 802.2258.

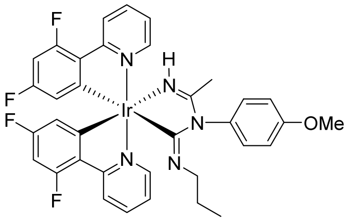

nF$_2$ppy$^{PhOMe/Me}$

nF$_2$ppy$^{PhOMe/Me}$: Prepared following the general procedure using **F$_2$ppy$^{PhOMe/Me}$** (0.016 mmol, 15 mg), NaOMe (4.0 equiv., 0.064 mmol, 3.5 mg), and MeOH (3.0 mL). The product was obtained as a yellow solid. ^{1}H NMR. Yield: 9.4 mg, 74%. ^{1}H NMR (400 MHz, $CDCl_3$) δ 8.93 (d, *J* = 5.9 Hz, 1H, Ar*H*), 8.48 (d, *J* = 5.8 Hz, 1H, Ar*H*), 8.28 (d, *J* = 8.5 Hz, 2H, Ar*H*), 7.78 (t, *J* = 7.8 Hz, 1H, Ar*H*), 7.72 (t, *J* = 7.8 Hz, 1H, Ar*H*), 7.09 (t, *J* = 6.9 Hz, 2H, Ar*H*), 6.68–6.75 (m, 4H, Ar*H*), 6.39–6.22 (m, 3H, Ar*H*), 5.82 (dd, *J* = 9.3, 2.4 Hz, 1H, Ar*H*), 5.68 (dd, *J* = 7.6, 2.4 Hz, 1H, Ar*H*), 3.80 (s, 3H, OC*H*$_3$), 2.81 (td, *J* = 10.4, 5.6 Hz, 1H, NC*H*$_2$), 2.64 (td, *J* = 10.3, 5.6 Hz, 1H, NC*H*$_2$), 2.03 (s, 3H, C*H*$_3$), 0.99–0.85 (m, 1H, C*H*$_2$CH$_3$), 0.59–0.46 (m, 1H, C*H*$_2$CH$_3$), 0.26 (t, *J* = 7.3 Hz, 3H, C*H*$_2$CH$_3$). The N*H* peak overlaps with one of the aromatic peaks. ^{19}F NMR (376 MHz, $CDCl_3$) δ −109.21 (br, s, 1F), −110.35 to −110.62 (br, m, 2F), −111.43 (br, s, 1F). HRMS-ESI: (m/z): $[M+H]^+$ calcd for $C_{35}H_{30}F_4IrN_5O$, 804.2023; found, 804.2054.

Reaction of F_2ppy$^{tBu/Cl}$ with propylamine and MeCN at 60 °C.

When **F_2ppy$^{tBu/Cl}$** was used as the substrate and the reaction was carried out at 60 °C instead of room temperature, a product in which the chloro ligand is substituted by propylamine was isolated. It was characterized by ^{1}H and ^{19}F NMR and single-crystal X-ray diffraction (the molecular structure is shown in Fig. S1).

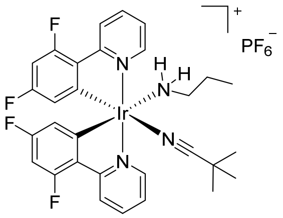

F_2ppy$^{tBu/NH2Pr}$

F_2ppy$^{tBu/NH2Pr}$. A 20-mL vial equipped with a magnetic stir bar was charged with **F_2ppy$^{tBu/Cl}$** (0.14 mmol, 0.10 g), propylamine (excess, 0.5 mL), and MeCN (excess, 3.0 mL). This mixture was stirred at 60 °C overnight. Upon completion, the solvent was removed under vacuum. The reaction vial was then transferred to a glove box and added a saturated solution of NH_4PF_6 in methanol, which was prepared by dissolving 100 mg of NH_4PF_6 in 3.0 mL of MeOH. The mixture was stirred for another 4 hours. After that, the solvent was removed under vacuum. The mixture was redissolved in CH_2Cl_2, filtered to remove excess NH_4PF_6, and subjected to column chromatography (silica gel, CH_2Cl_2 then ethyl acetate/CH_2Cl_2 1:10 v/v). The crude product was dissolved in a minimum amount of CH_2Cl_2. An excess amount of hexane was then added to precipitate the product out of the mixture, which was then filtered and dried under vacuum. The product was obtained as a yellow solid. Yield: 27 mg, 22%.^{1}H NMR (500 MHz, $CDCl_3$) δ 9.03 (ddd, J = 5.8, 1.6, 0.7 Hz, 1H, Ar*H*), 8.68 (ddd, J = 5.9, 1.6, 0.7 Hz, 1H, Ar*H*), 8.39–8.32 (m, 2H, Ar*H*), 8.03–7.95 (m, 2H, Ar*H*), 7.51 (ddd, J = 7.4, 5.8, 1.5 Hz, 1H, Ar*H*), 7.38 (ddd, J = 7.4, 5.8, 1.4 Hz, 1H, Ar*H*), 6.52–6.38 (m, 2H, Ar*H*), 5.55 (dd, J = 8.5, 2.3 Hz, 1H, Ar*H*), 5.52 (dd, J = 7.9, 2.3 Hz, 1H, Ar*H*), 3.62–3.52 (m, 1H, NH_2), 3.16–3.06 (m, 1H, NH_2), 2.10–2.01 (m, 2H, NCH_2), 1.49–1.40 (m, 2H, C$H_2$$CH_3$), 1.38 (s, 9H, C(C$H_3$)$_3$), 0.62 (t, J = 7.4 Hz, 3H, CH_2CH_3). ^{19}F NMR (470 MHz, $CDCl_3$) δ −71.36 (d, $J_{F\text{–}P}$ = 714 Hz, 6F, PF_6), −104.81 to −104.98 (m, 1F), −106.67 (q, J = 9.3 Hz, 1F), −108.05 (t, J = 11.7 Hz, 1F), −109.26 (t, J = 11.8 Hz, 1F). HRMS-ESI: (m/z): $[M-PF_6]^+$ calcd for $C_{30}H_{30}F_{10}IrN_4P$, 713.1999; found, 713.1993.

Table S1. Summary of unsuccessful outcomes when screening substrates for the three-component reaction.

Substrate	Reactions	Outcome
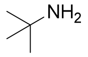 *tert*-butylamine 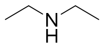 diethylamine	With **$F_2ppy^{dmp/Cl}$** and MeCN at room temperature and 60 °C	No reaction, recovery of **$F_2ppy^{dmp/Cl}$** starting material
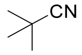 pivalonitrile	With **$F_2ppy^{dmp/Cl}$** and propylamine at room temperature and 60 °C	Intractable mixture of products, no clear evidence for desired imino-ADC product
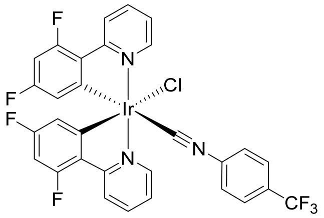 **$F_2ppy^{PhCF3/Cl}$**	With propylamine and MeCN at room temperature and 60 °C	Intractable mixture of products, no clear evidence for desired imino-ADC product
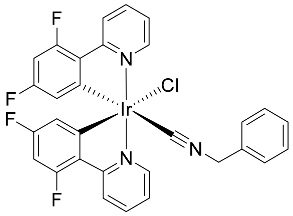 **$F_2ppy^{Bn/Cl}$**	With propylamine and MeCN at room temperature and 60 °C	Room temperature: No reaction, recovery of **$F_2ppy^{Bn/Cl}$** starting material<br>60 °C: Intractable mixture of products, no clear evidence for desired imino-ADC product
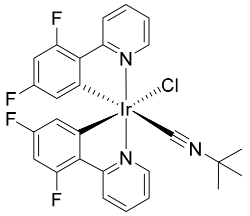 **$F_2ppy^{tBu/Cl}$**	With propylamine and MeCN at room temperature and 60 °C	Room temperature: No reaction, recovery of **$F_2ppy^{tBu/Cl}$** starting material<br><br>60 °C: Formation and isolation of **$F_2ppy^{tBu/NH2Pr}$**

Proposed reaction mechanism

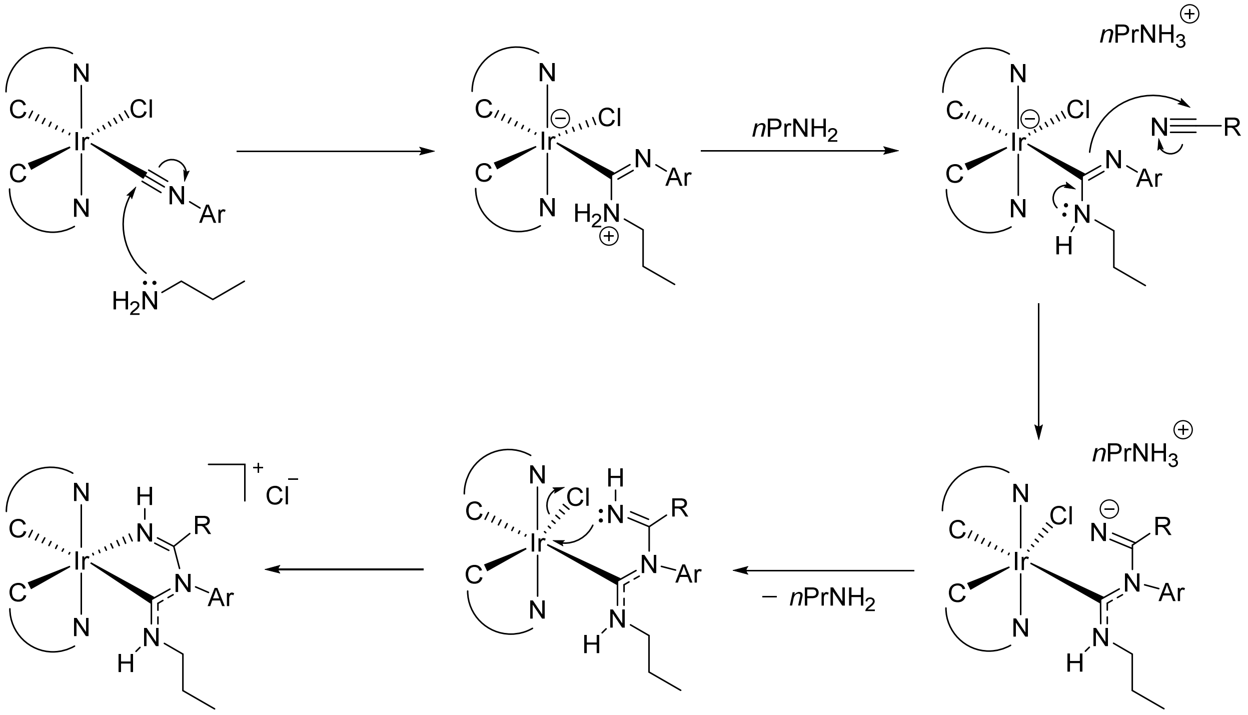

Scheme S1. Proposed mechanism for the three-component reaction.

Table S2. Summary of X-ray crystallographic data for **F$_2$ppy$^{dmp/Me}$**, **F$_2$ppy$^{dipp/Me}$**, and **F$_2$ppy$^{dmp/nPr}$**.

	**F$_2$ppy$^{dmp/Me}$**	**F$_2$ppy$^{dipp/Me}$**	**F$_2$ppy$^{dmp/nPr}$**
CCDC	2518473	2518474	2518478
**Crystal data**			
Chemical formula	$C_{36}H_{33}F_4IrN_5·PF_6$	$C_{40}H_{41}F_4IrN_5·PF_6$	$C_{38}H_{37}F_4IrN_5·PF_6$
M_r	948.84	1004.95	976.89
Crystal system, space group	Monoclinic, $P2_1/n$	Monoclinic, $P2_1/n$	Monoclinic, $P2_1/n$
Temperature (K)	150	150	150
a, *b*, *c* (Å)	10.688(8), 15.418(11), 22.417(18)	10.8929(8), 31.960(2), 12.6053(9)	12.3528(17), 20.663(3), 15.486(2)
β (°)	103.354(9)	115.058(1)	103.924(2)
V (Å^3)	3594(5)	3975.3(5)	3836.7(9)
Z	4	4	4
Radiation type	Mo $K\alpha$	Mo $K\alpha$	Mo $K\alpha$
μ (mm^{-1})	3.85	3.48	3.61
Crystal size (mm)	0.31 × 0.28 × 0.04	0.31 × 0.23 × 0.06	0.24 × 0.14 × 0.02
**Data collection**			
Diffractometer	Bruker *APEX*-II CCD	Bruker *APEX*-II CCD	Bruker *APEX*-II CCD
Absorption correction	Multi-scan *SADABS* (Bruker, 2016/2) was used for absorption correction. wR2(int) was 0.0888 before and 0.0542 after correction. The ratio of minimum to maximum transmission is 0.6980. The λ/2 correction factor is not present.	Multi-scan *SADABS* (Bruker, 2016/2) was used for absorption correction. wR2(int) was 0.1005 before and 0.0303 after correction. The ratio of minimum to maximum transmission is 0.7531. The λ/2 correction factor is not present.	Multi-scan *SADABS* (Bruker, 2016/2) was used for absorption correction. wR2(int) was 0.0716 before and 0.0321 after correction. The ratio of minimum to maximum transmission is 0.7690. The λ/2 correction factor is not present.
T_{min}, T_{max}	0.520, 0.746	0.562, 0.746	0.573, 0.746
No. of measured, independent and observed [$I > 2\sigma(I)$] reflections	25038, 8229, 6653	28460, 8982, 7874	21356, 8700, 6801
R_{int}	0.055	0.028	0.037
(sin θ/λ)$_{max}$ (Å^{-1})	0.649	0.649	0.647
**Refinement**			
$R[F^2 > 2\sigma(F^2)]$, $wR(F^2)$, S	0.032, 0.075, 1.00	0.026, 0.053, 1.06	0.029, 0.058, 0.97
No. of reflections	8229	8982	8700
No. of parameters	488	524	504
No. of restraints	2		1
H-atom treatment	H atoms treated by a mixture of independent and constrained refinement	H atoms treated by a mixture of independent and constrained refinement	H atoms treated by a mixture of independent and constrained refinement
$\Delta\rho_{max}$, $\Delta\rho_{min}$ (e Å^{-3})	1.53, −0.83	1.20, −1.18	0.82, −0.60

Table S3. Summary of X-ray crystallographic data for $\mathbf{F_2ppy^{dmp/Ph}}$, $\mathbf{ppz^{dmp/Me}}$, and $\mathbf{nF_2ppy^{PhOMe/Me}}$.

	$\mathbf{F_2ppy^{dmp/Ph}}\cdot CH_2Cl_2$	$\mathbf{ppz^{dmp/Me}}\cdot CH_2Cl_2$	$\mathbf{nF_2ppy^{PhOMe/Me}}$
CCDC	2518475	2518476	2518477
**Crystal data**			
Chemical formula	$C_{41}H_{35}F_4IrN_5\cdot PF_6\cdot CH_2Cl_2$	$C_{32}H_{32}IrN_7\cdot Cl\cdot CH_2Cl_2$	$C_{35}H_{30}F_4IrN_5O$
M_r	1095.83	827.22	804.84
Crystal system, space group	Triclinic, $P\bar{1}$	Orthorhombic, *Pnna*	Monoclinic, $P2_1/n$
Temperature (K)	150	150	200
a, *b*, *c* (Å)	9.8521(4), 11.6556(5), 18.7639 (8)	18.1935 (12), 30.004 (2), 12.5245 (8)	9.4811 (5), 14.0699 (7), 25.9038 (14)
α, β, γ (°)	83.419 (1), 77.668 (1) 88.607 (1)	90, 90, 90	90, 95.901 (1) 90
V (Å^3)	2091.11 (15)	6836.9 (8)	3437.2 (3)
Z	2	8	4
Radiation type	Mo *K*α	Mo *K*α	Mo *K*α
μ (mm^{-1})	3.44	4.17	3.94
Crystal size (mm)	0.22 × 0.19 × 0.07	0.32 × 0.11 × 0.08	0.19 × 0.09 × 0.06
**Data collection**			
Diffractometer	Bruker *APEX*-II CCD	Bruker *APEX*-II CCD	Bruker *APEX*-II CCD
Absorption correction	Multi-scan *SADABS* (Bruker, 2016/2) was used for absorption correction. wR2(int) was 0.0589 before and 0.0364 after correction. The ratio of minimum to maximum transmission is 0.8302. The λ/2 correction factor is not present.	Multi-scan *SADABS* (Bruker, 2016/2) was used for absorption correction. wR2(int) was 0.0670 before and 0.0399 after correction. The ratio of minimum to maximum transmission is 0.7371. The λ/2 correction factor is not present.	Multi-scan *SADABS*
T_{min}, T_{max}	0.619, 0.746	0.550, 0.746	0.627, 0.746
No. of measured, independent and observed [$I > 2\sigma(I)$] reflections	20901, 9036, 8356	47306, 7882, 5022	24327, 7913, 6170
R_{int}	0.027	0.083	0.035
$(\sin \theta/\lambda)_{max}$ (Å^{-1})	0.641	0.651	0.651
**Refinement**			
$R[F^2 > 2\sigma(F^2)]$, $wR(F^2)$, S	0.024, 0.058, 1.05	0.045, 0.121, 1.04	0.026, 0.060, 0.99
No. of reflections	9036	7882	7913
No. of parameters	559	422	432
No. of restraints	2	408	3
H-atom treatment	H atoms treated by a mixture of independent and constrained refinement	H-atom parameters constrained $w = 1/[\sigma^2(F_o^2) + (0.0442P)^2 + 41.0075P]$ where $P = (F_o^2 + 2F_c^2)/3$	H atoms treated by a mixture of independent and constrained refinement
$\Delta\rho_{max}$, $\Delta\rho_{min}$ (e Å^{-3})	1.62, −0.72	3.15, −1.36	1.11, −0.95

Table S4. Summary of X-ray crystallographic data for $\mathbf{F_2ppy}^{t\mathbf{Bu/NH2Pr}}$.

	$\mathbf{F_2ppy}^{t\mathbf{Bu/NH2Pr}}\cdot 2(CH_2Cl_2)$
CCDC	2518662
**Crystal data**	
Chemical formula	$C_{30}H_{30}F_4IrN_4\cdot PF_6\cdot 2(CH_2Cl_2)$
M_r	1029.60
Crystal system, space group	Monoclinic, $P2_1/c$
Temperature (K)	150
a, b, c (Å)	13.2853(5), 22.0638(7), 13.3648(4)
β (°)	98.195(1)
V (Å^3)	3877.5(2)
Z	4
Radiation type	Mo $K\alpha$
μ (mm^{-1})	3.84
Crystal size (mm)	0.35 × 0.21 × 0.08
**Data collection**	
Diffractometer	Bruker *APEX*-II CCD
Absorption correction	Multi-scan<br>*SADABS* (Bruker, 2016/2) was used for absorption correction. wR2(int) was 0.0650 before and 0.0331 after correction. The ratio of minimum to maximum transmission is 0.6726. The λ/2 correction factor is not present.
T_{min}, T_{max}	0.501, 0.746
No. of measured, independent and observed [$I > 2\sigma(I)$] reflections	25401, 8852, 7614
R_{int}	0.066
$(\sin \theta/\lambda)_{max}$ (Å^{-1})	0.649
**Refinement**	
$R[F^2 > 2\sigma(F^2)]$, $wR(F^2)$, S	0.051, 0.143, 1.07
No. of reflections	8852
No. of parameters	510
No. of restraints	551
H-atom treatment	H-atom parameters constrained<br><br>$w = 1/[\sigma^2(F_o^2) + (0.0809P)^2 + 14.6749P]$<br>where $P = (F_o^2 + 2F_c^2)/3$
$\Delta\rho_{max}$, $\Delta\rho_{min}$ (e Å^{-3})	4.00, −2.21

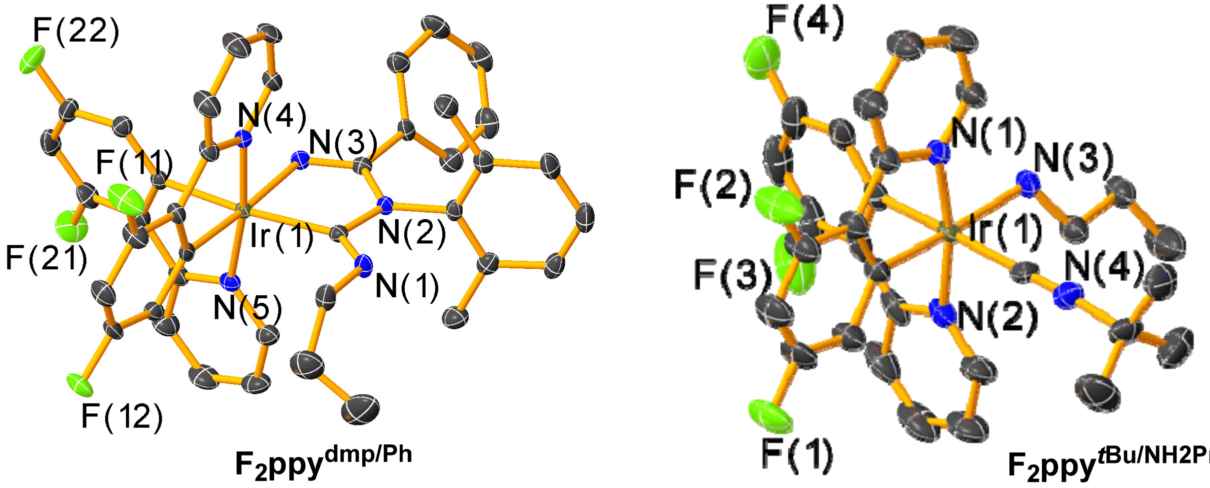

Fig. S1. Molecular structures of $\mathbf{F_2ppy^{dmp/Ph}}$ and $\mathbf{F_2ppy^{\mathit{t}Bu/NH2Pr}}$, determined by single-crystal X-ray diffraction. Thermal ellipsoids are drawn at the 50% probability level with the solvent molecules, counterion, and hydrogen atoms eliminated.

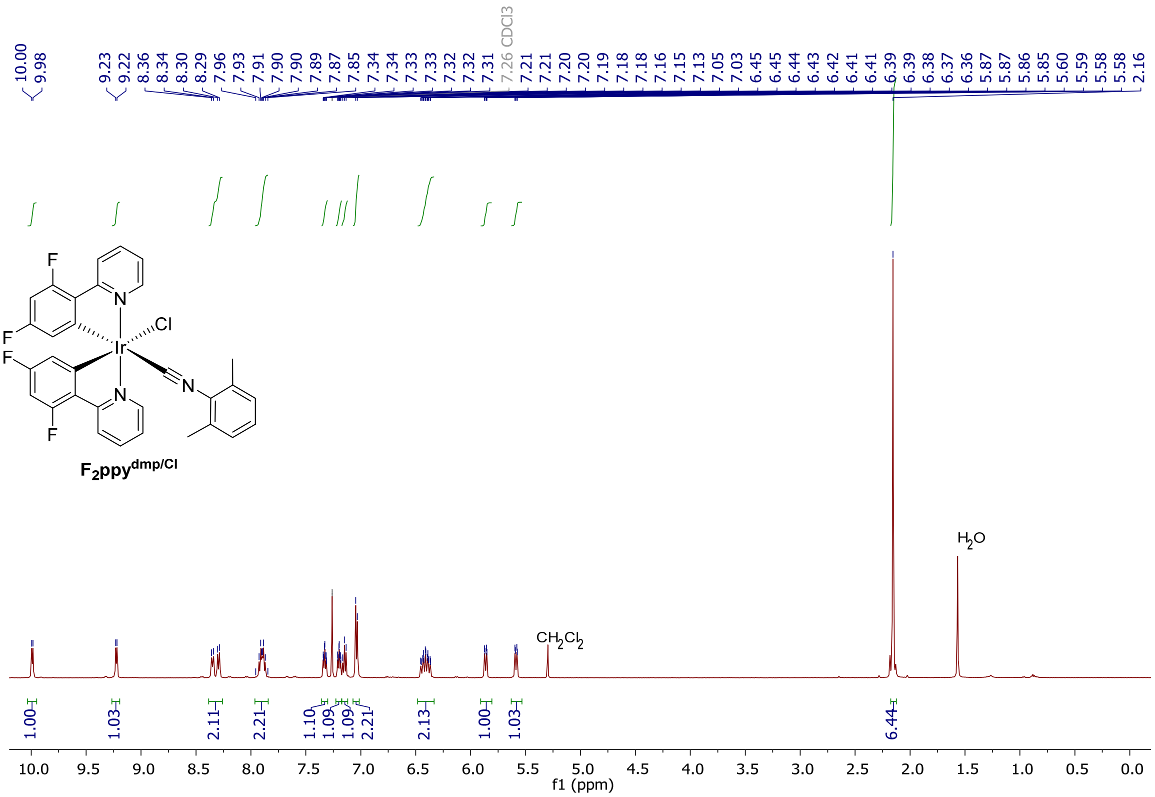

Fig. S2. ^{1}H NMR spectrum of complex **$F_2ppy^{dmp/Cl}$**, recorded in chloroform-*d* at 500 MHz.

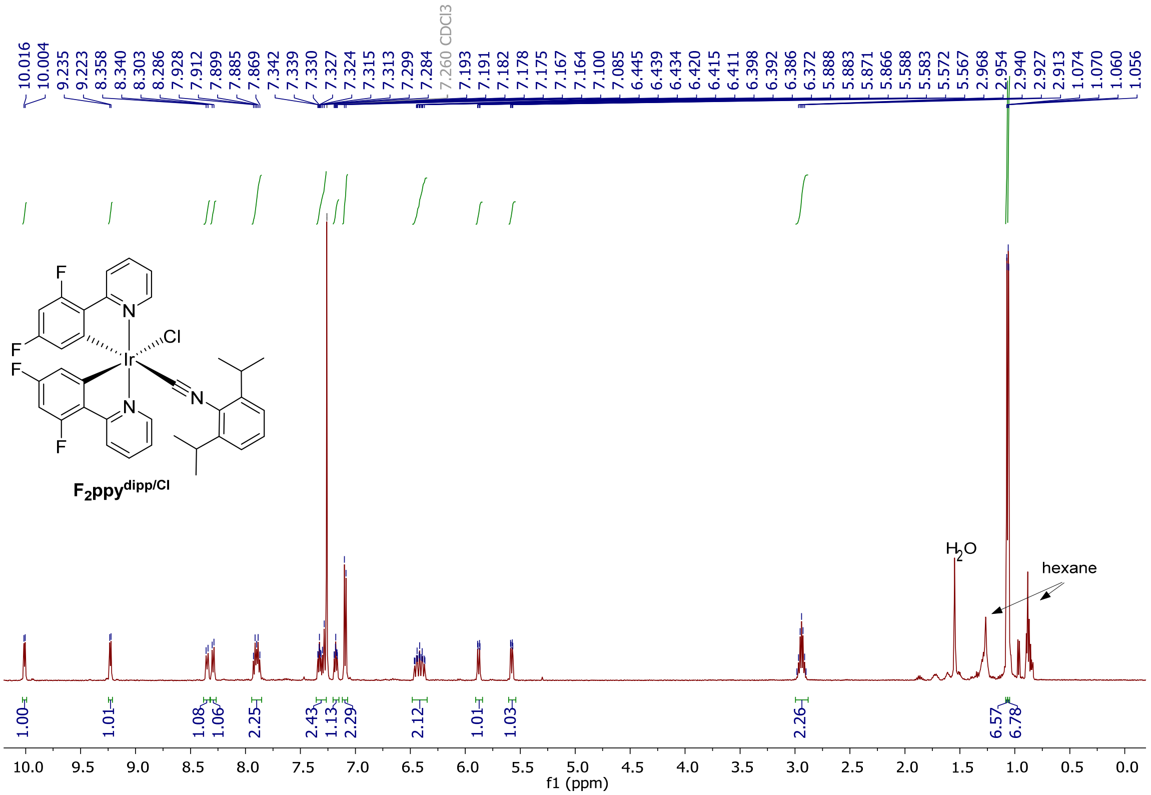

Fig. S3. ^{1}H NMR spectrum of complex **$F_2ppy^{dipp/Cl}$**, recorded in chloroform-*d* at 500 MHz.

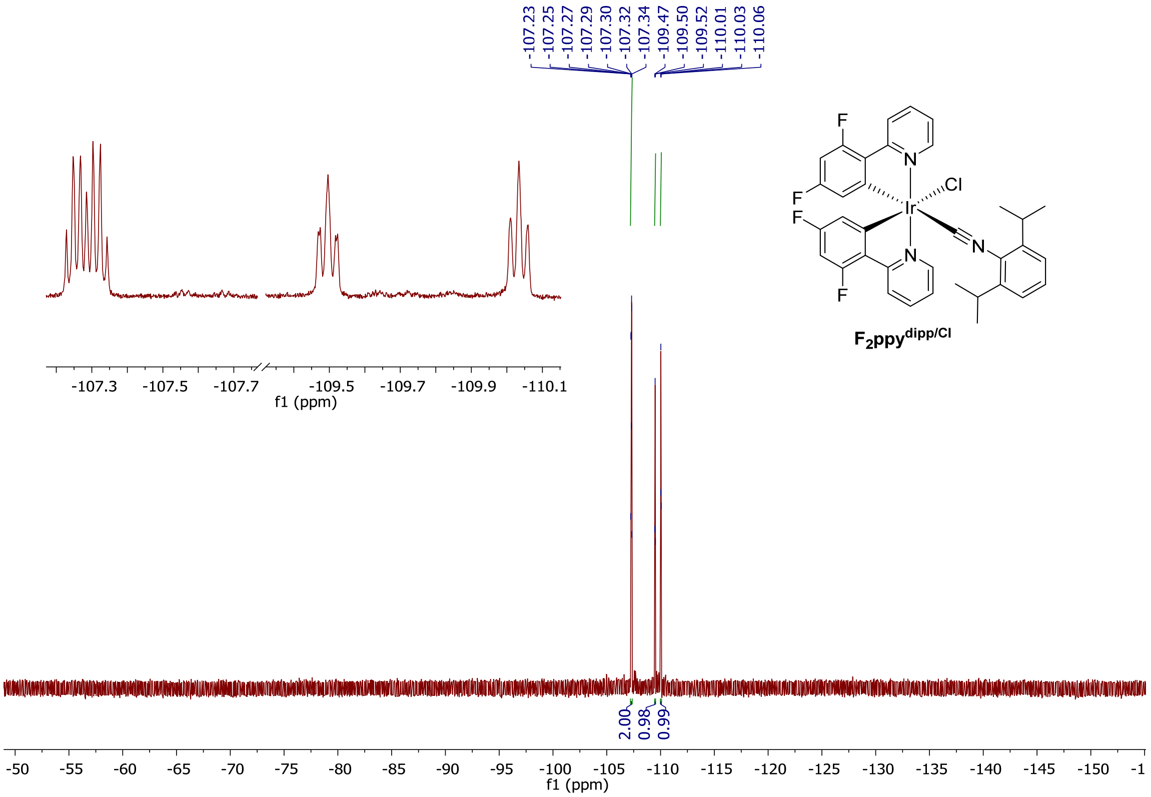

Fig. S4. ^{19}F NMR spectrum of complex **F₂ppy$^{\mathbf{dipp/Cl}}$**, recorded in chloroform-*d* at 470 MHz.

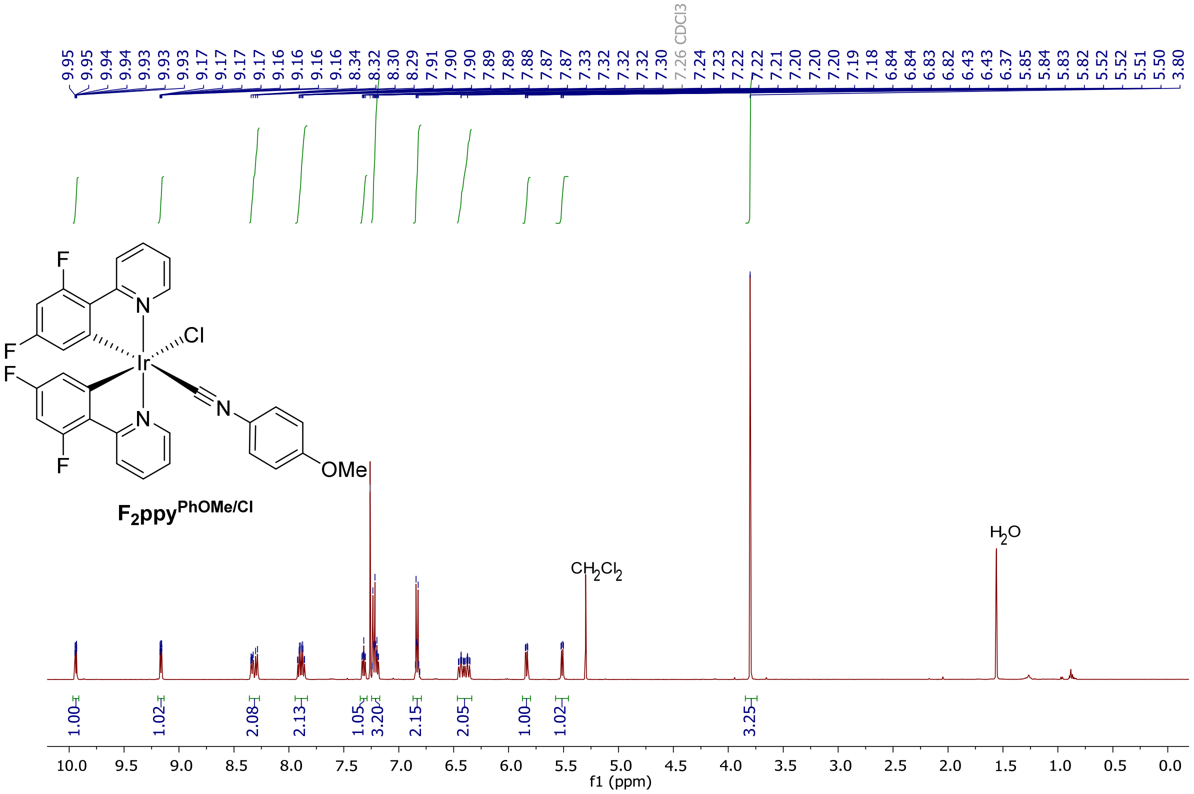

Fig. S5. ^{1}H NMR spectrum of complex **F₂ppy$^{\mathbf{PhOMe/Cl}}$**, recorded in chloroform-*d* at 500 MHz.

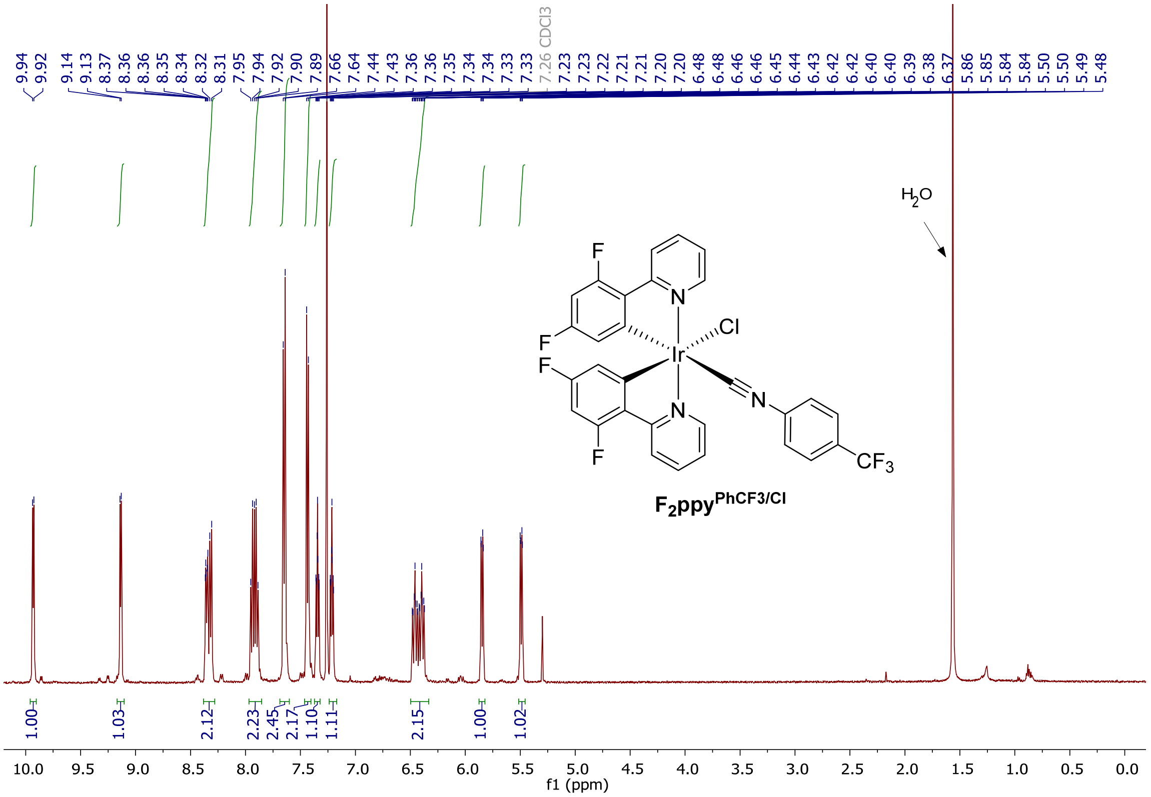

Fig. S6. ^{1}H NMR spectrum of complex **$F_2ppy^{PhCF3/Cl}$**, recorded in chloroform-*d* at 500 MHz.

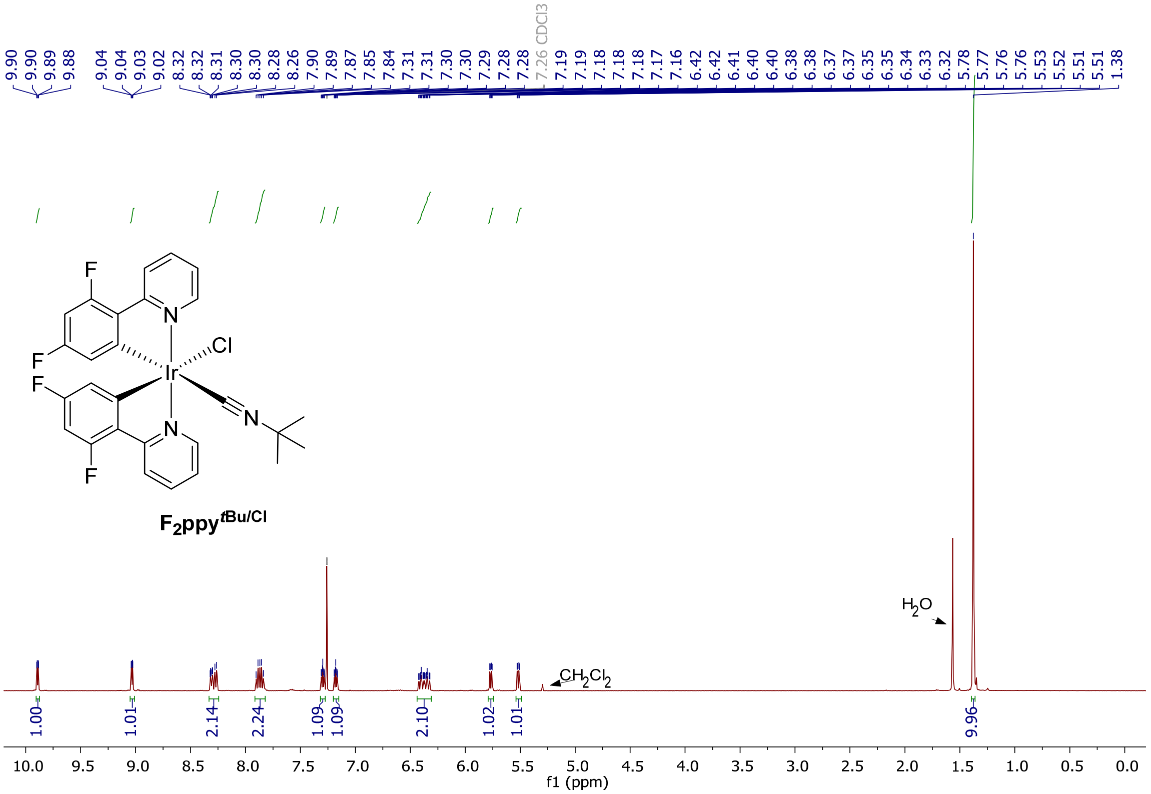

Fig. S7. ^{1}H NMR spectrum of complex **$F_2ppy^{tBu/Cl}$**, recorded in chloroform-*d* at 500 MHz.

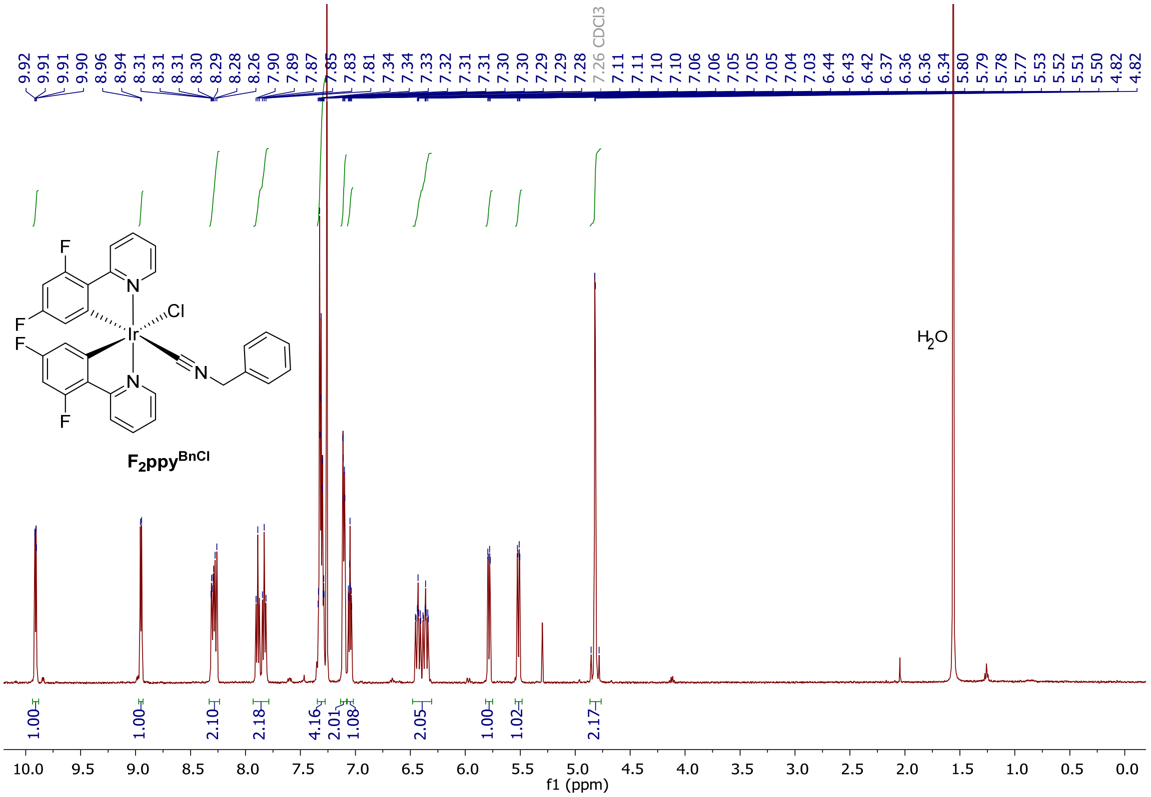

Fig. S8. ^{1}H NMR spectrum of complex **F$_2$ppy$^{Bn/Cl}$**, recorded in chloroform-*d* at 500 MHz.

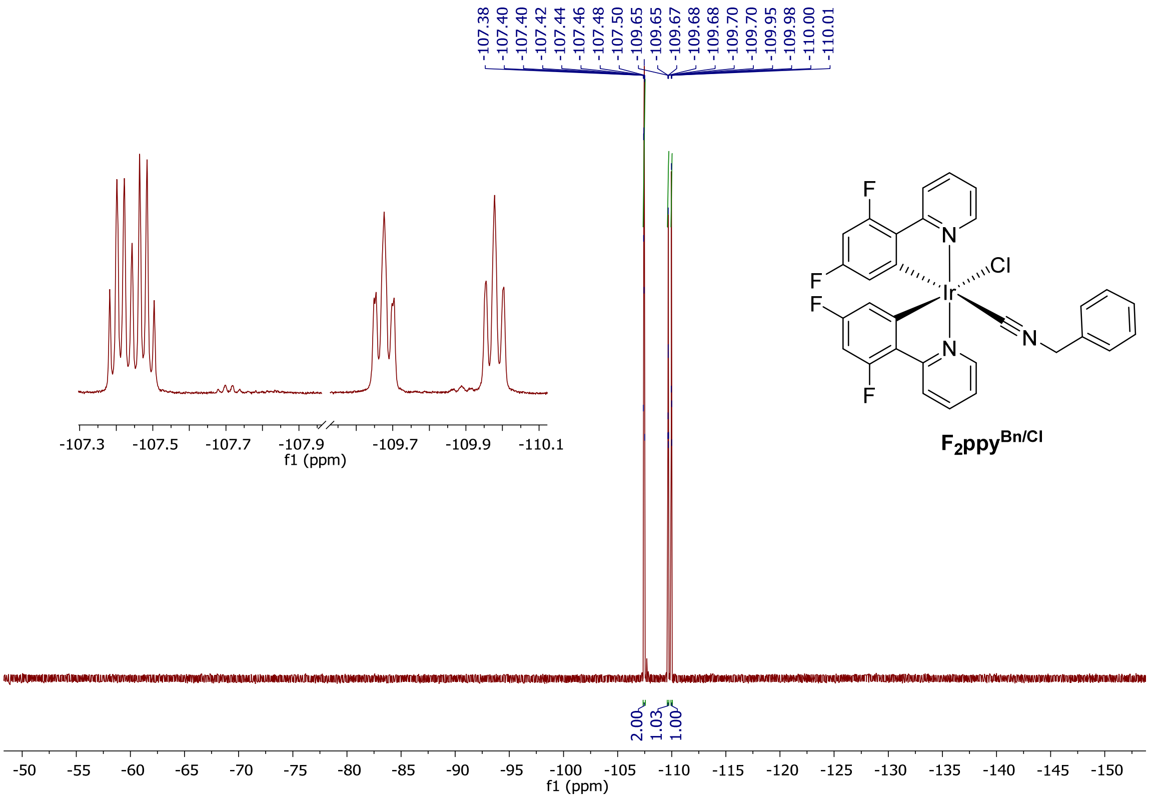

Fig. S9. ^{19}F NMR spectrum of complex **F$_2$ppy$^{Bn/Cl}$**, recorded in chloroform-*d* at 470 MHz.

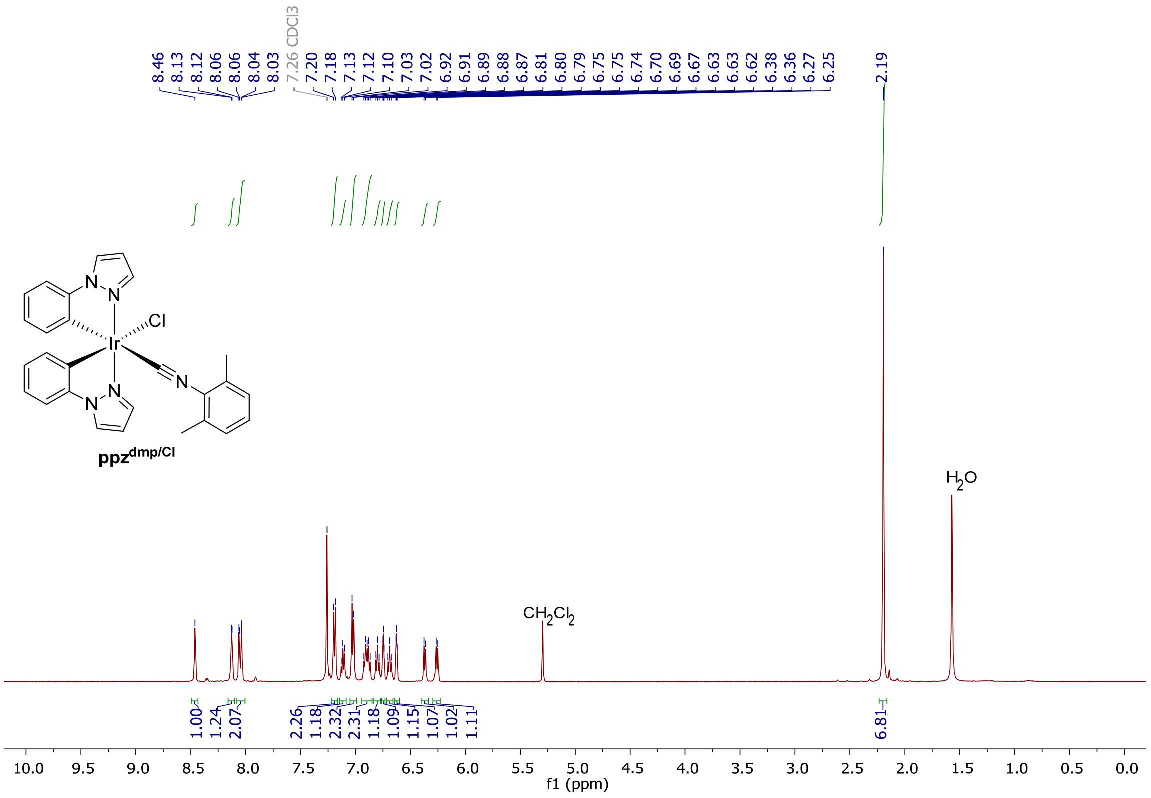

Fig. S10. ^{1}H NMR spectrum of complex **ppz$^{dmp/Cl}$**, recorded in chloroform-*d* at 500 MHz.

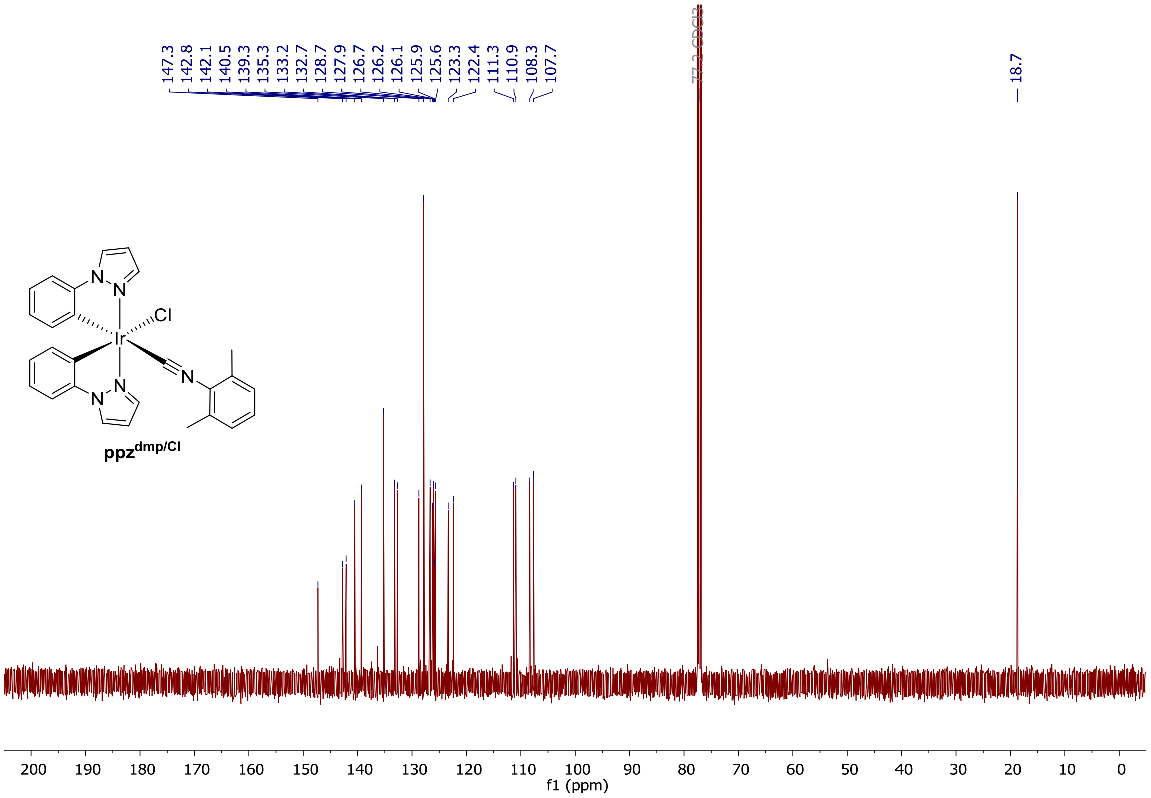

Fig. S11. ^{13}C{^{1}H} NMR spectrum of complex **ppz$^{dmp/Cl}$**, recorded in chloroform-*d* at 126 MHz.

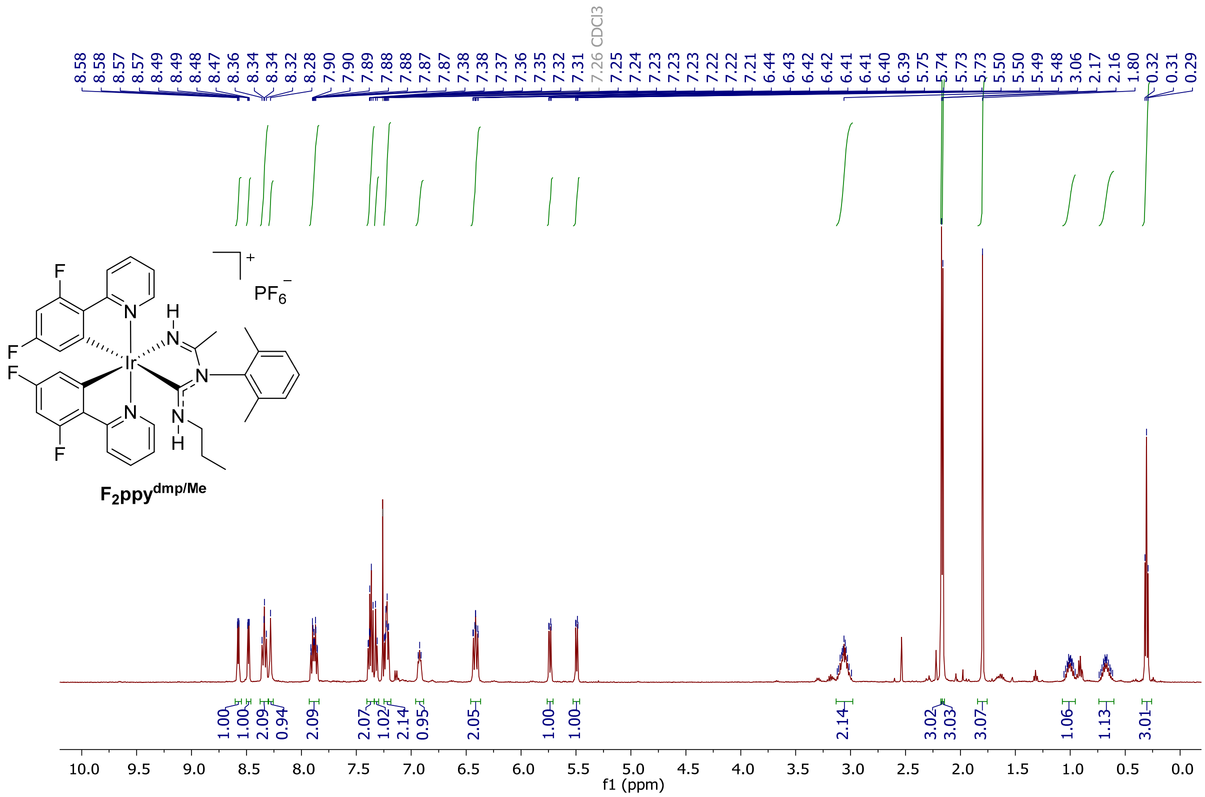

Fig. S12. ^{1}H NMR spectrum of complex **F$_2$ppy$^{dmp/Me}$**, recorded in chloroform-*d* at 500 MHz.

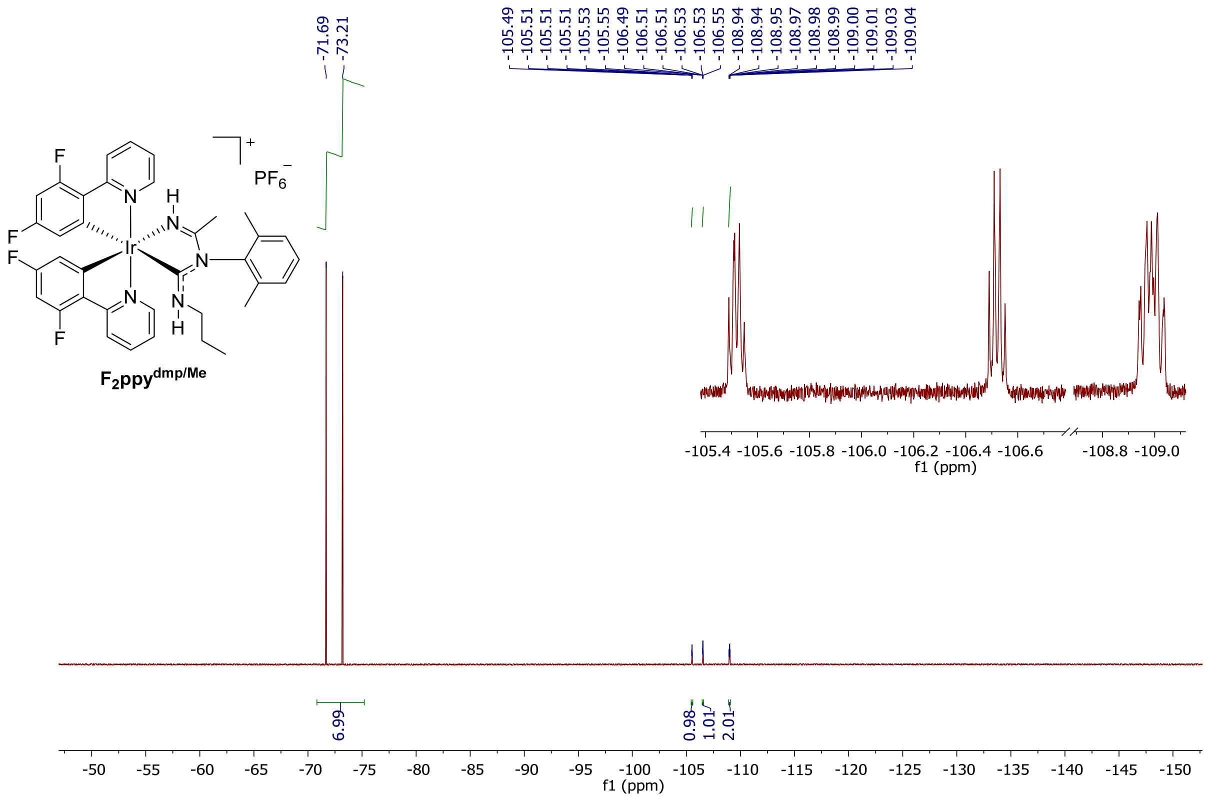

Fig. S13. ^{19}F NMR spectrum of complex **F$_2$ppy$^{dmp/Me}$**, recorded in chloroform-*d* at 470 MHz.

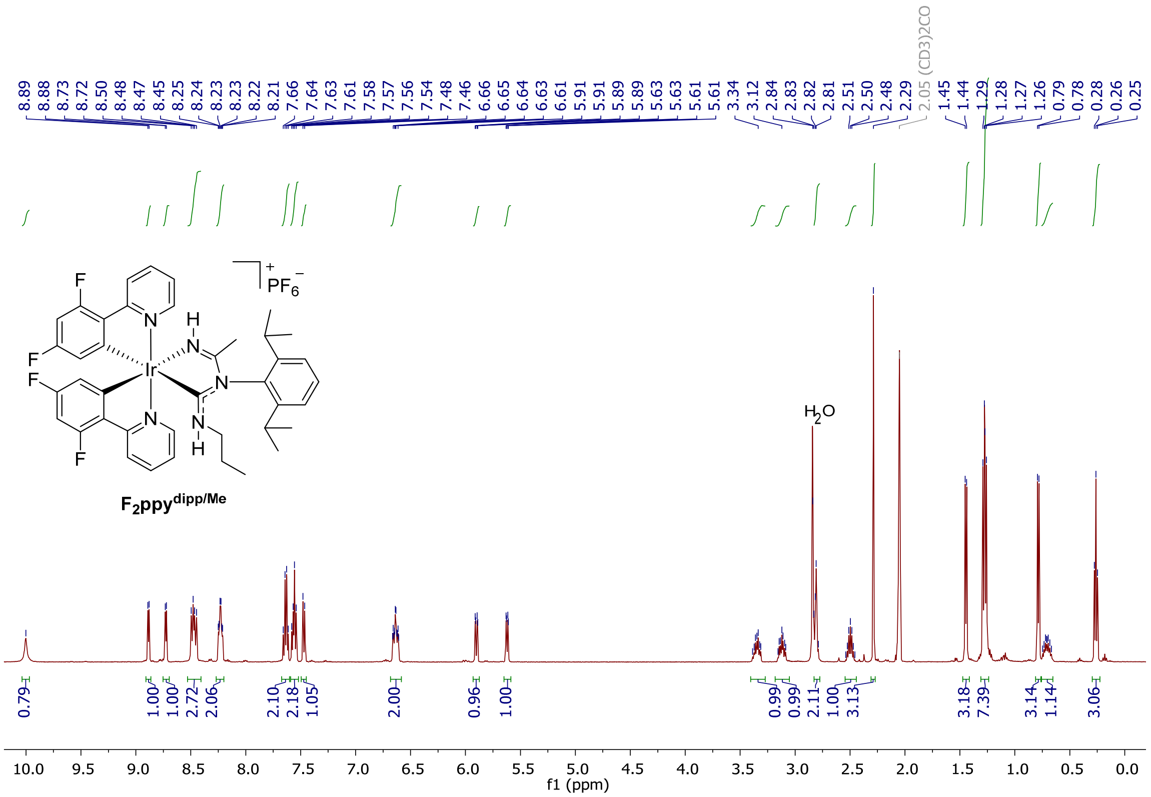

Fig. S14. ^{1}H NMR spectrum of complex **F$_2$ppy$^{dipp/Me}$**, recorded in acetone-d_6 at 500 MHz.

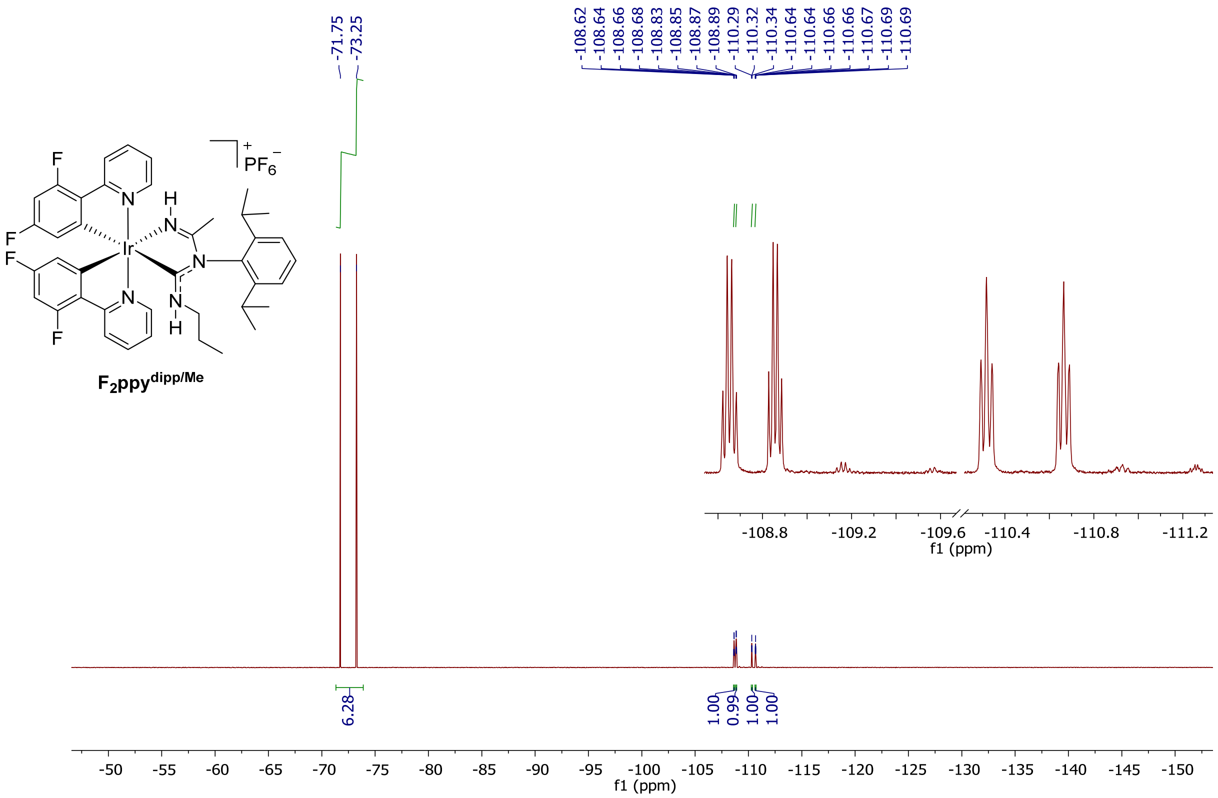

Fig. S15. ^{19}F NMR spectrum of complex **F$_2$ppy$^{dipp/Me}$**, recorded in acetone-d_6 at 470 MHz.

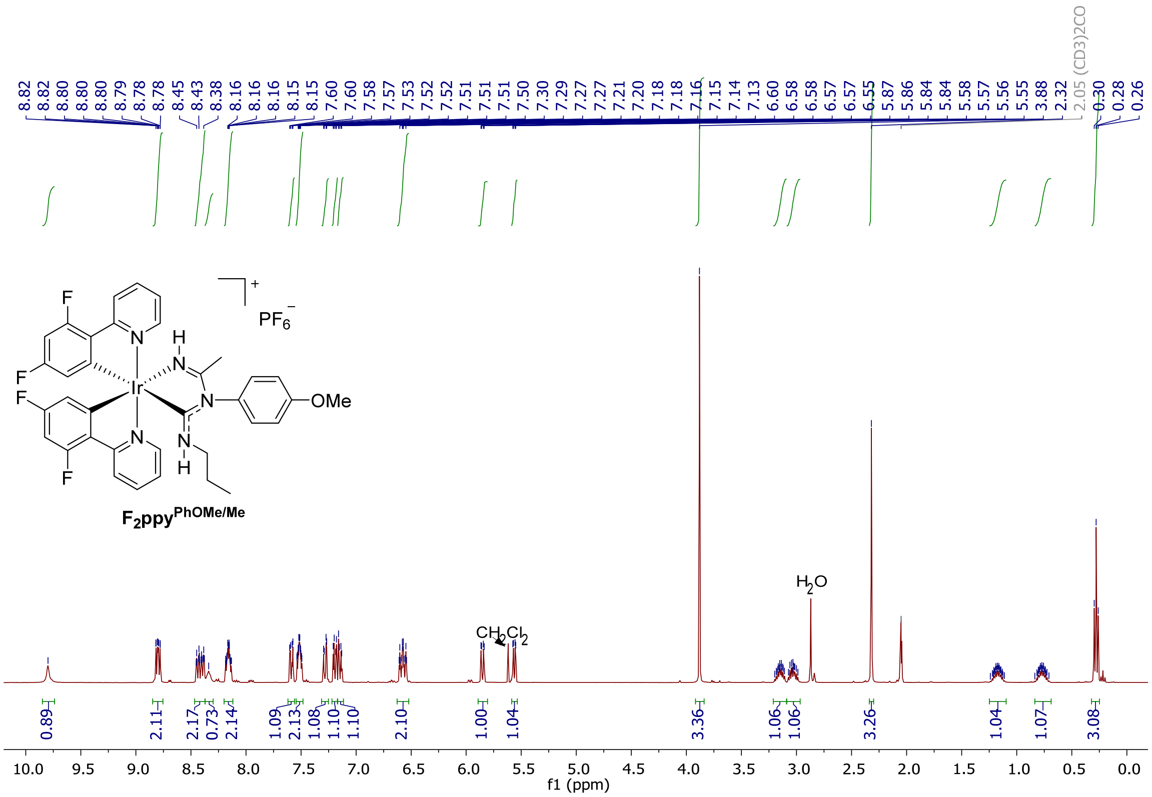

Fig. S16. ^{1}H NMR spectrum of complex **F$_2$ppy$^{PhOMe/Me}$**, recorded in acetone-d_6 at 400 MHz.

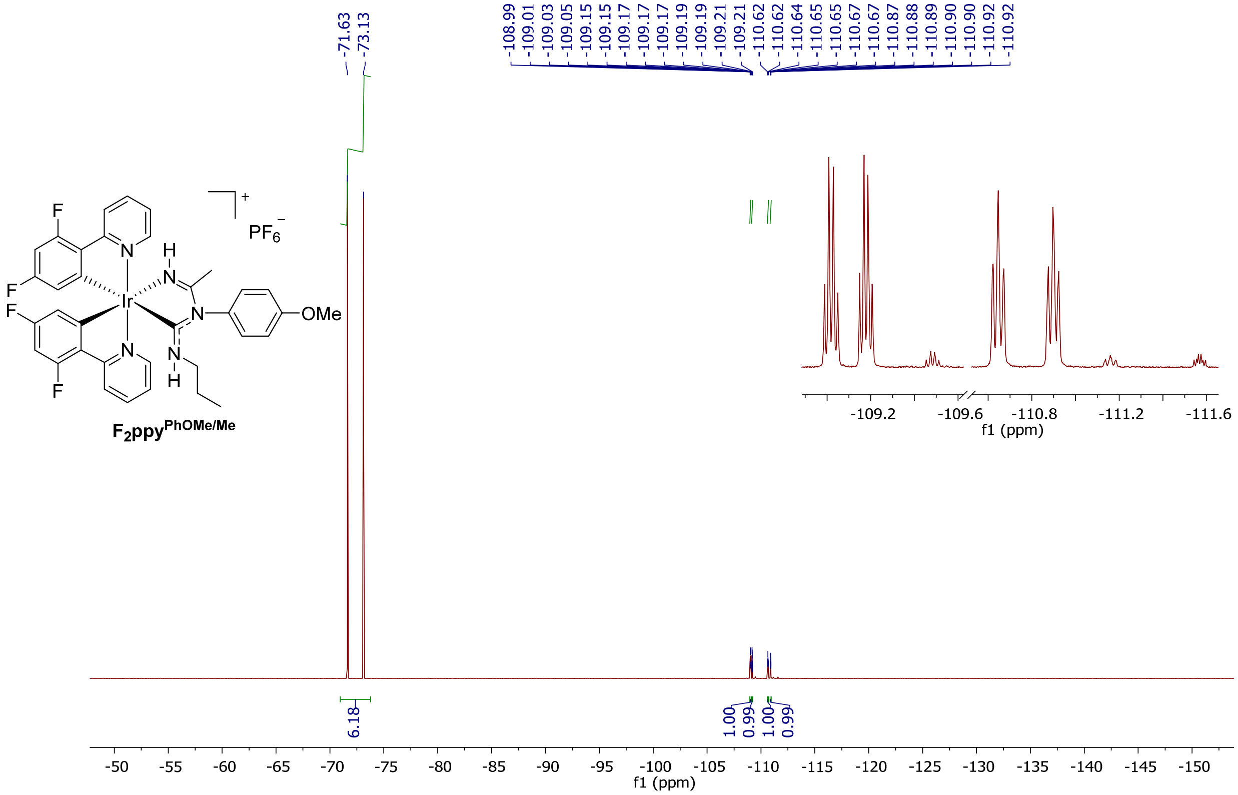

Fig. S17. ^{19}F NMR spectrum of complex **F$_2$ppy$^{PhOMe/Me}$**, recorded in acetone-d_6 at 376 MHz.

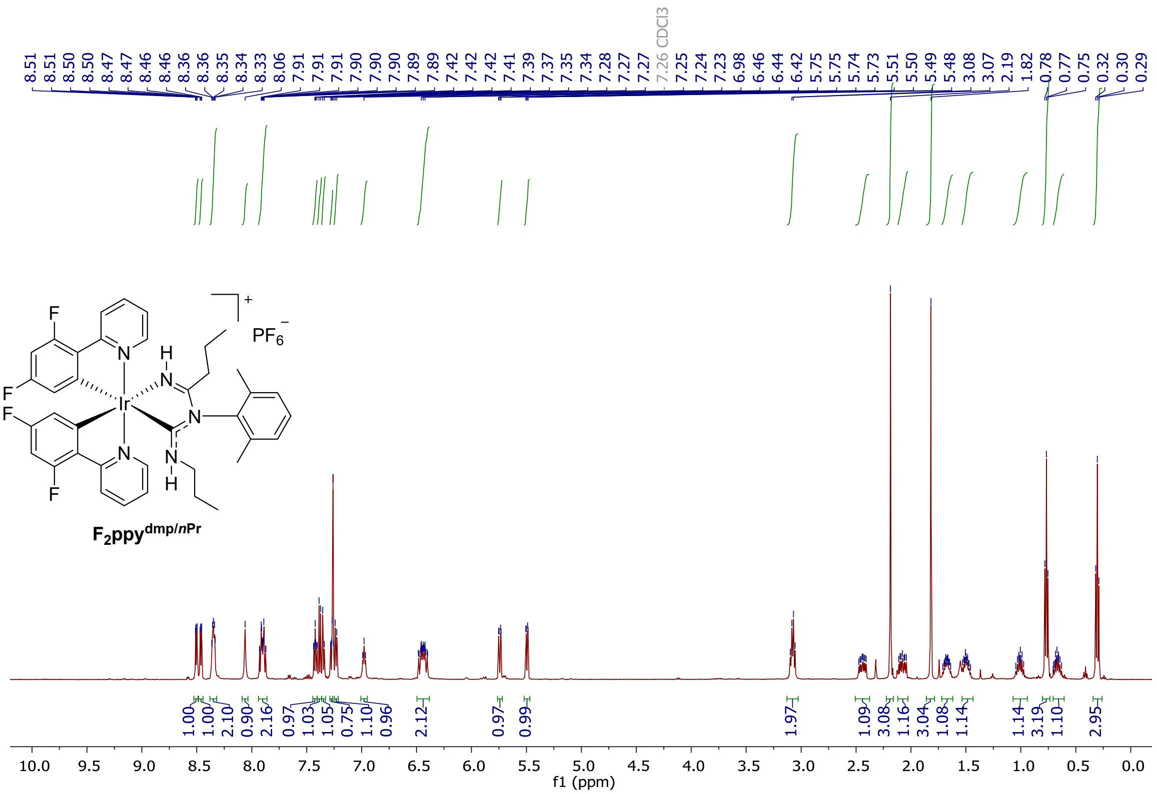

Fig. S18. ^{1}H NMR spectrum of complex **F₂ppy$^{dmp/nPr}$**, recorded in chloroform-*d* at 500 MHz.

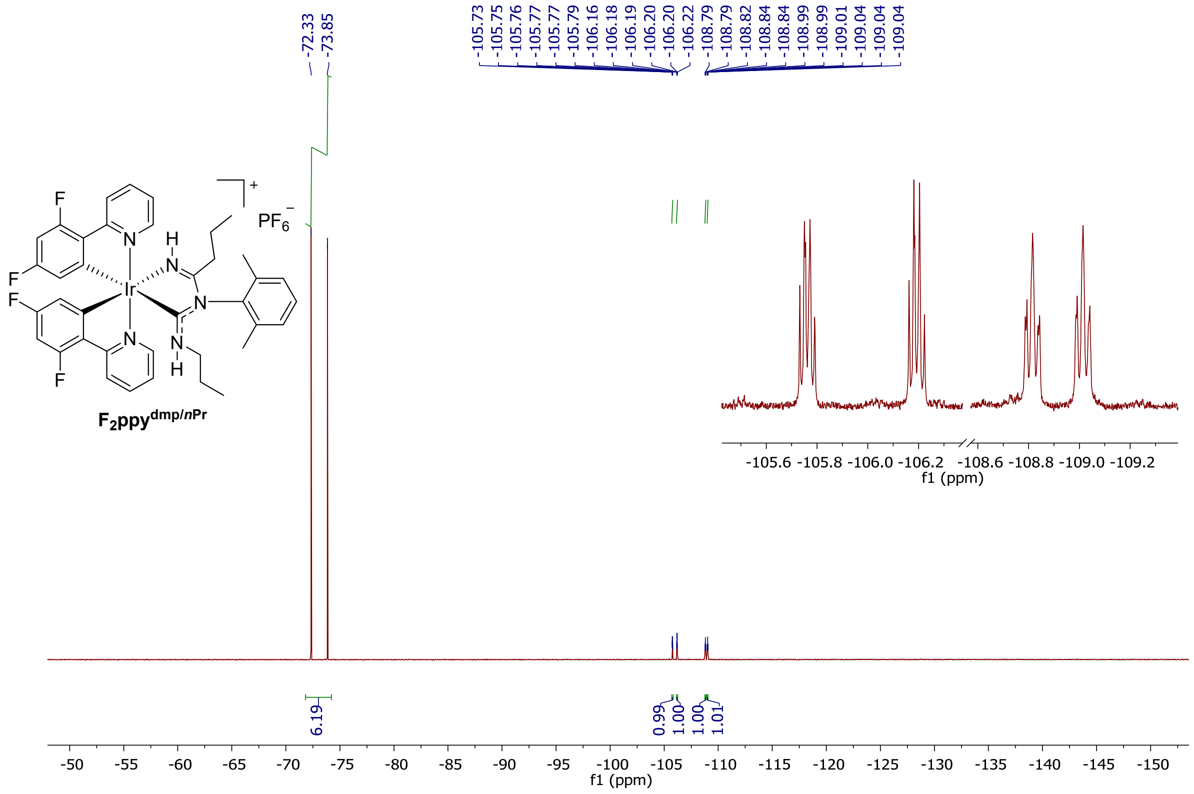

Fig. S19. ^{19}F NMR spectrum of complex **F₂ppy$^{dmp/nPr}$**, recorded in chloroform-*d* at 470 MHz.

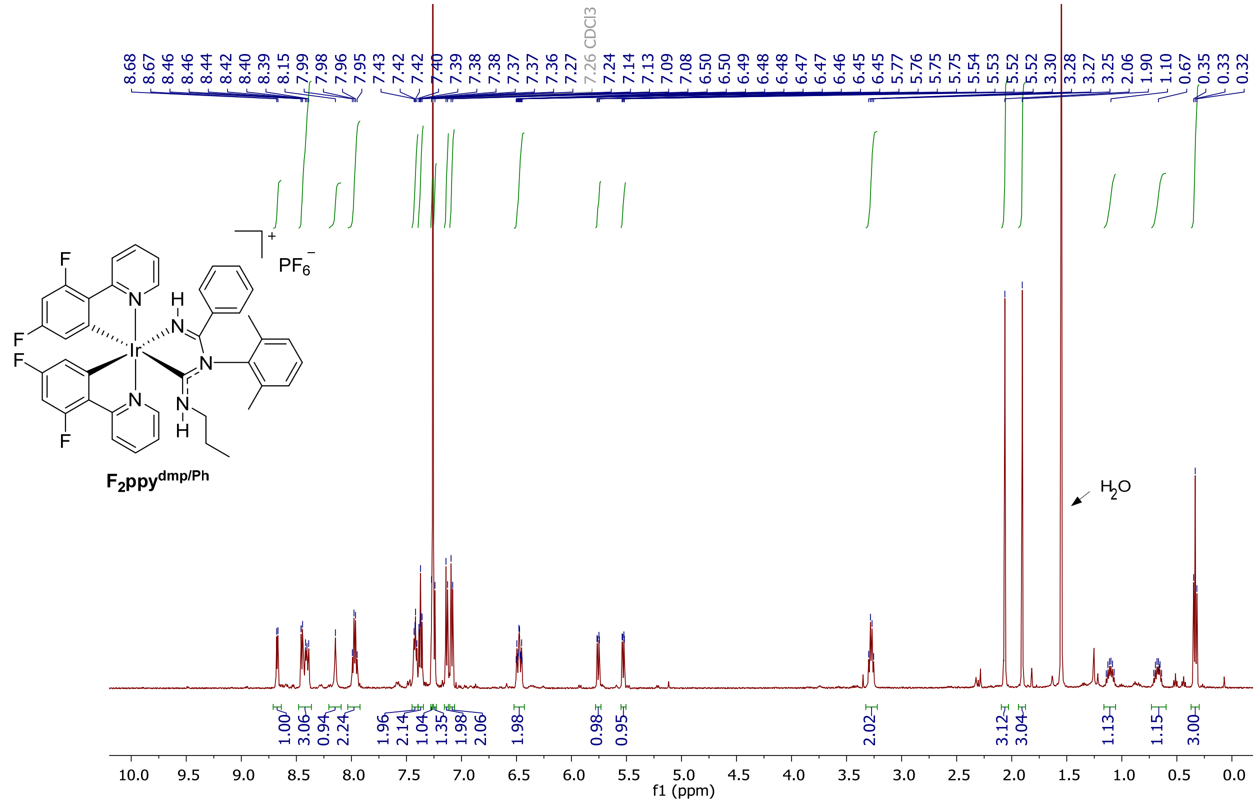

Fig. S20. ^{1}H NMR spectrum of complex **F₂ppy^dmp/Ph**, recorded in chloroform-*d* at 500 MHz.

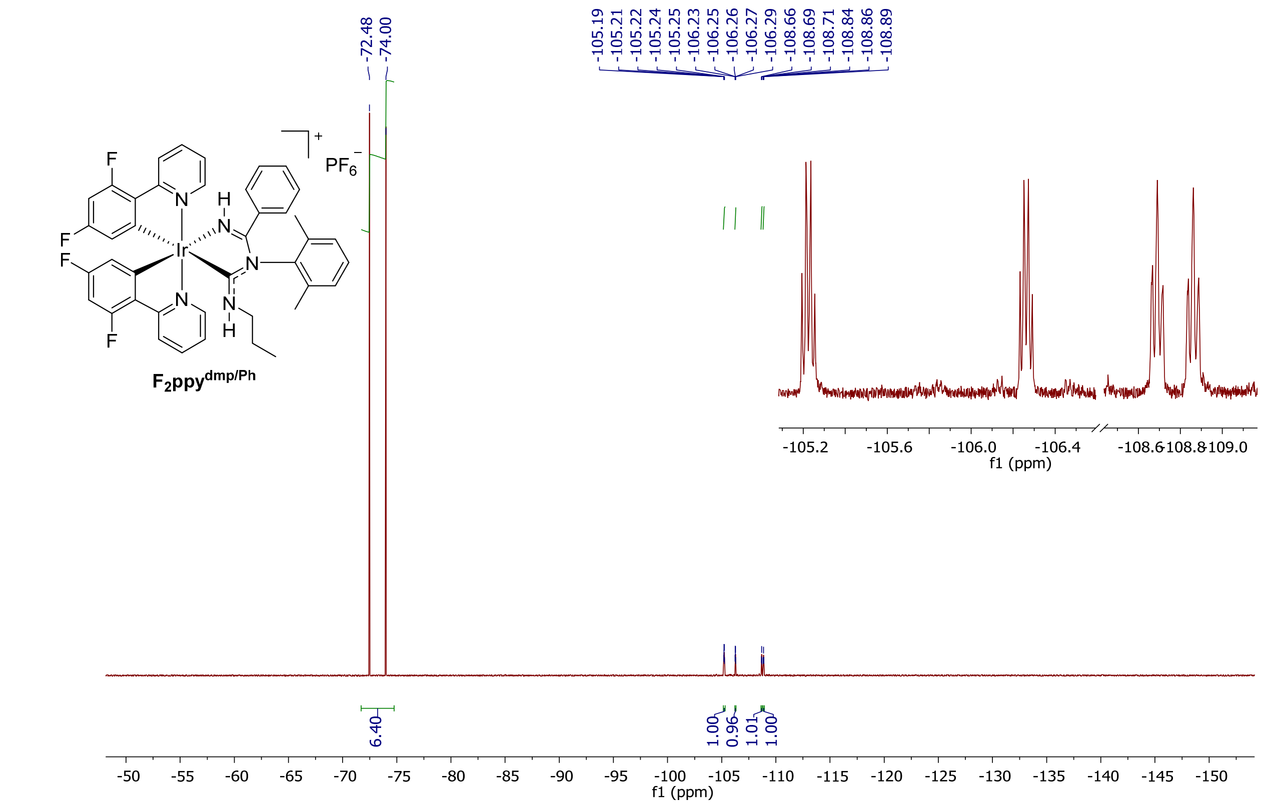

Fig. S21. ^{19}F NMR spectrum of complex **F$_2$ppy$^{dmp/Ph}$**, recorded in chloroform-*d* at 470 MHz.

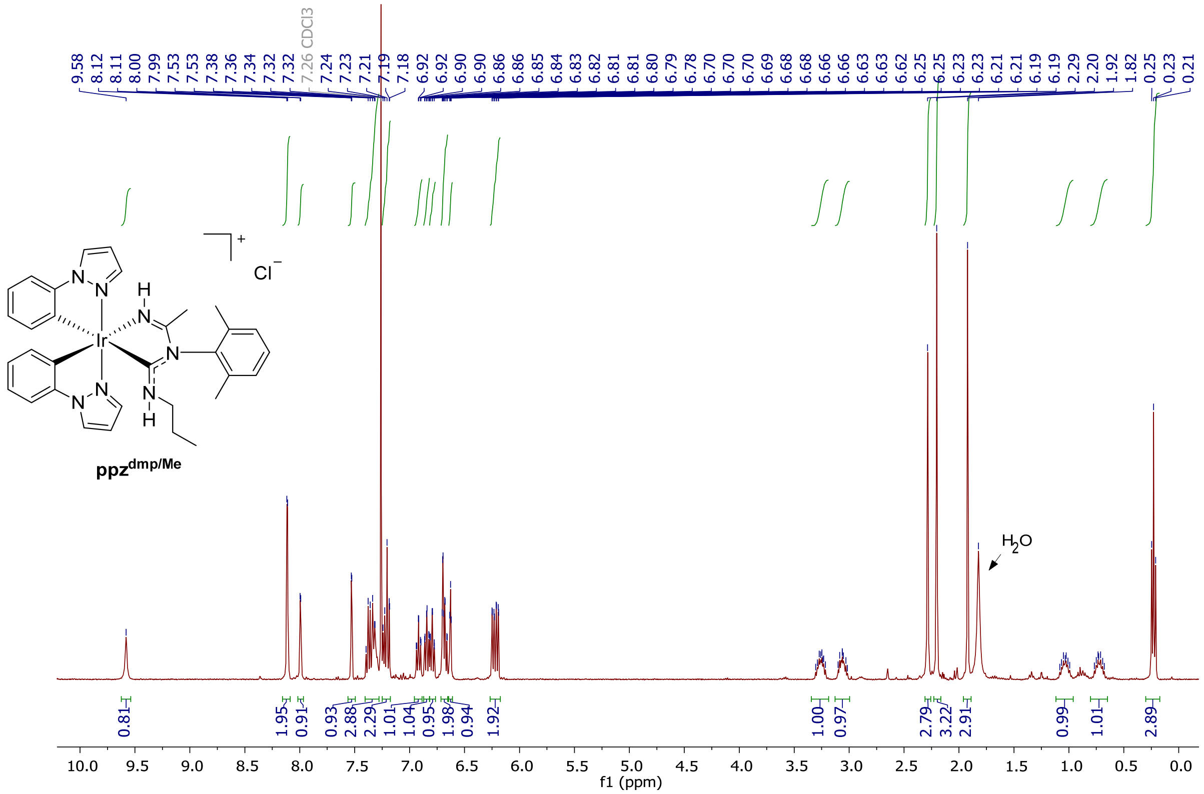

Fig. S22. ^{1}H NMR spectrum of complex **ppz$^{dmp/Me}$**, recorded in chloroform-*d* at 400 MHz.

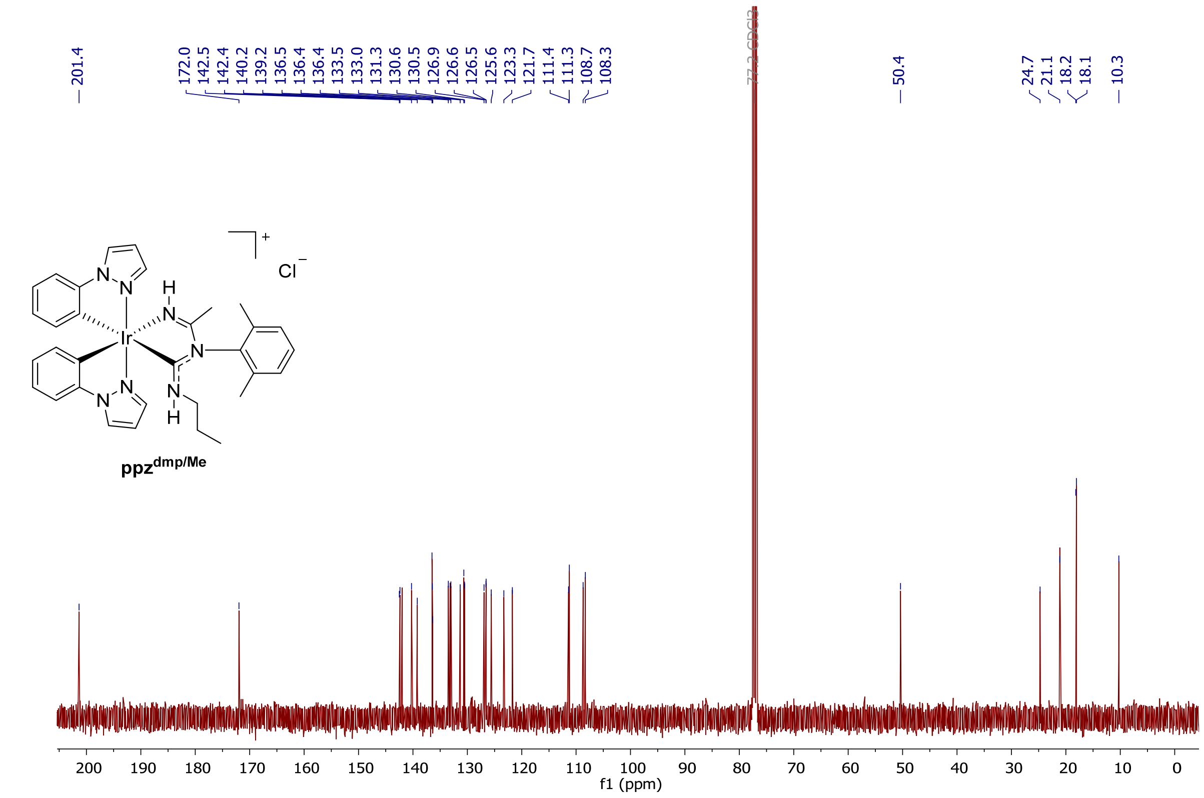

Fig. S23. $^{13}C\{^{1}H\}$ NMR spectrum of complex **ppz**$^{\mathbf{dmp/Me}}$, recorded in chloroform-*d* at 100 MHz.

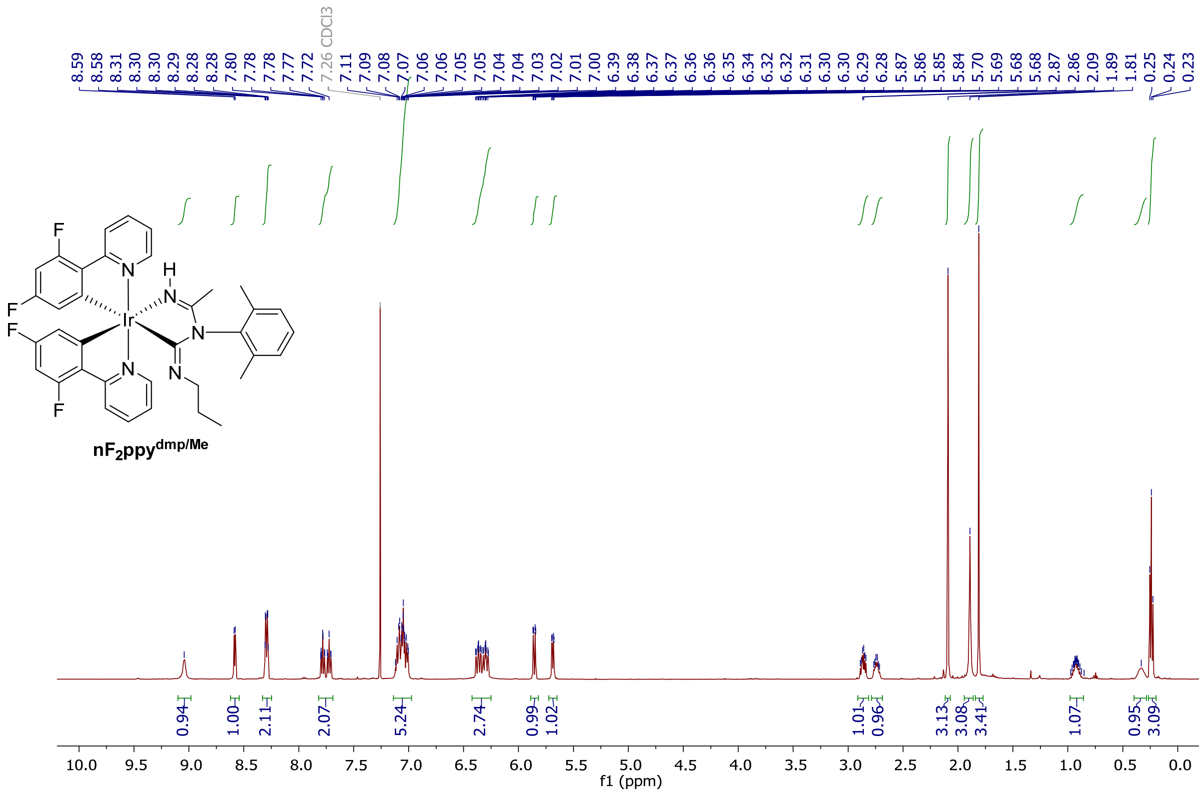

Fig. S24. ^{1}H NMR spectrum of complex **nF**$_{\mathbf{2}}$**ppy**$^{\mathbf{dmp/Me}}$, recorded in chloroform-*d* at 500 MHz.

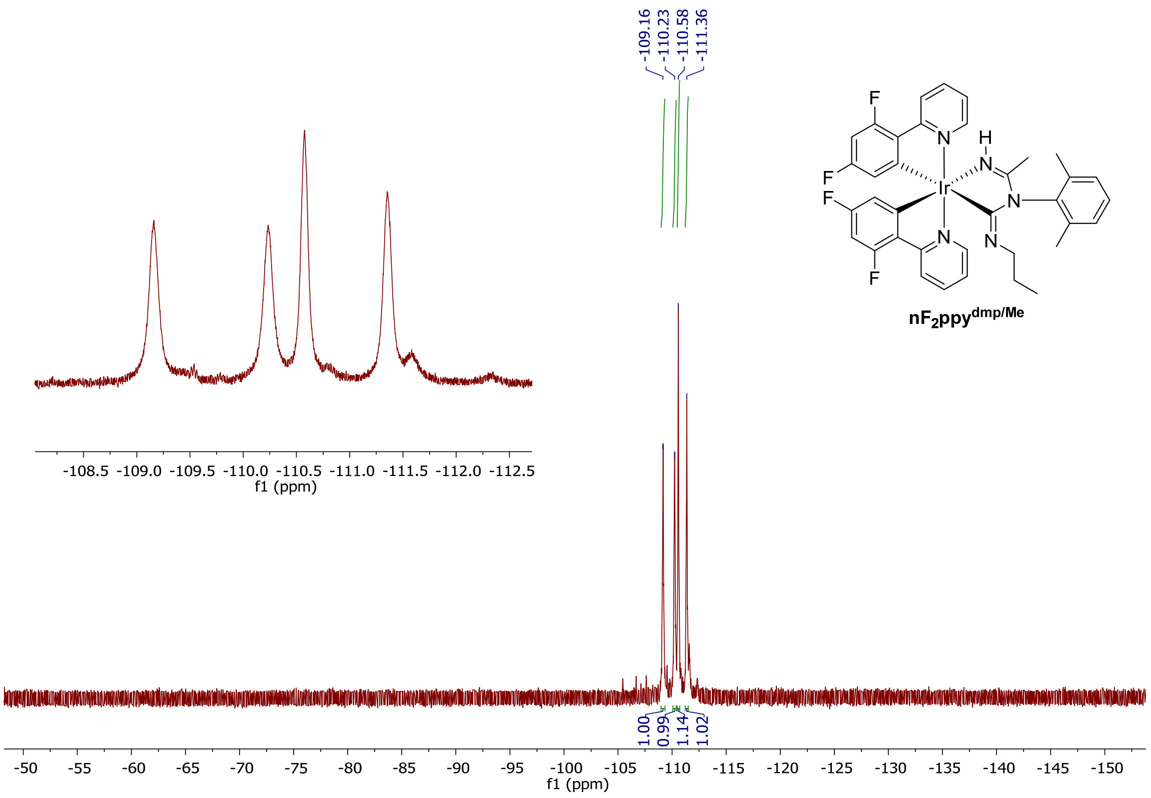

Fig. S25. ^{19}F NMR spectrum of complex **nF$_2$ppy$^{\mathbf{dmp/Me}}$**, recorded in chloroform-*d* at 470 MHz.

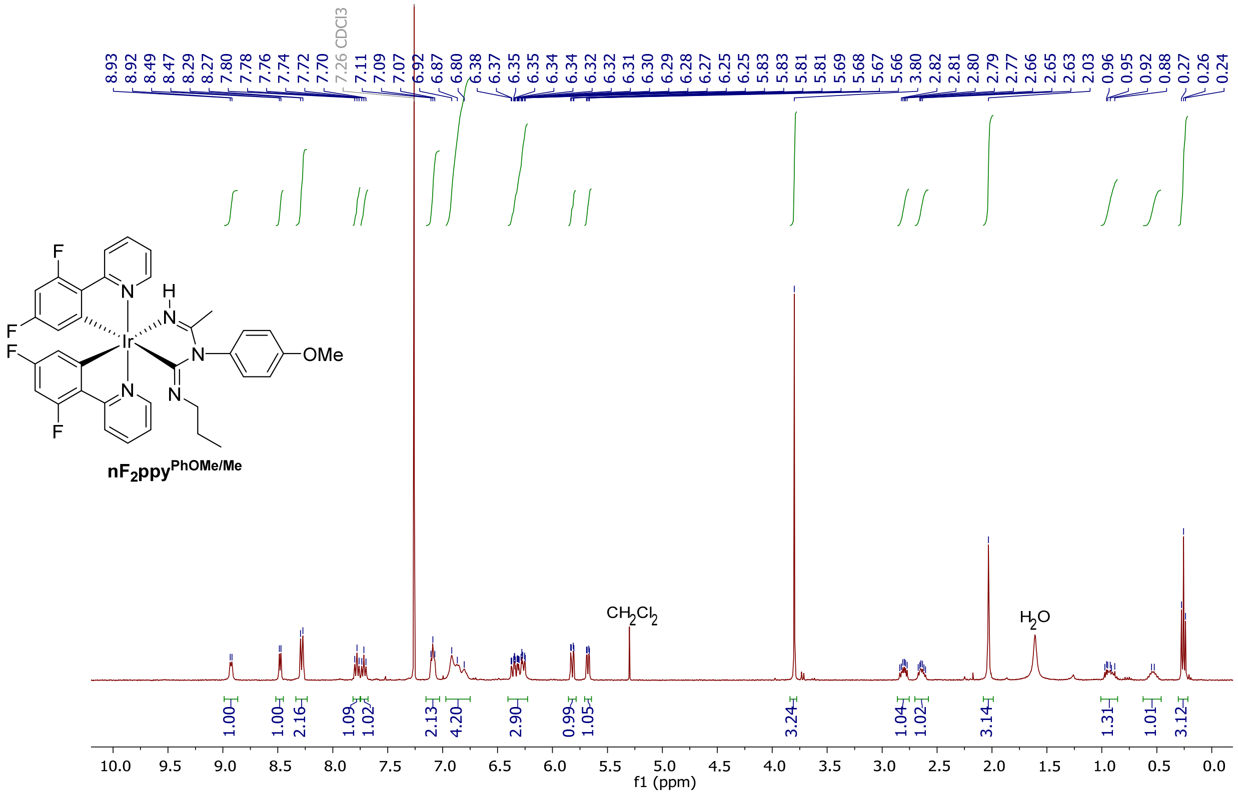

Fig. S26. ^{1}H NMR spectrum of complex **nF$_2$ppy$^{\mathbf{PhOMe/Me}}$**, recorded in chloroform-*d* at 400 MHz.

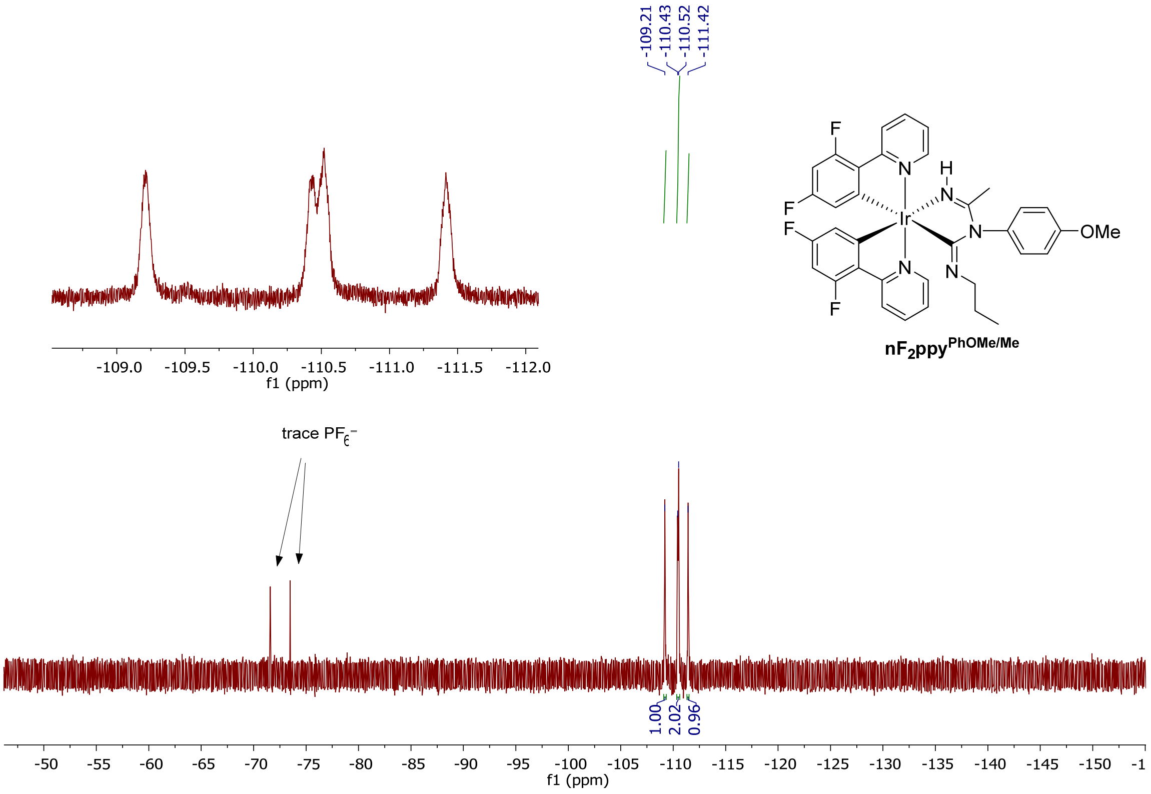

Fig. S27. ^{19}F NMR spectrum of complex **nF$_2$ppy$^{PhOMe/Me}$**, recorded in chloroform-*d* at 376 MHz.

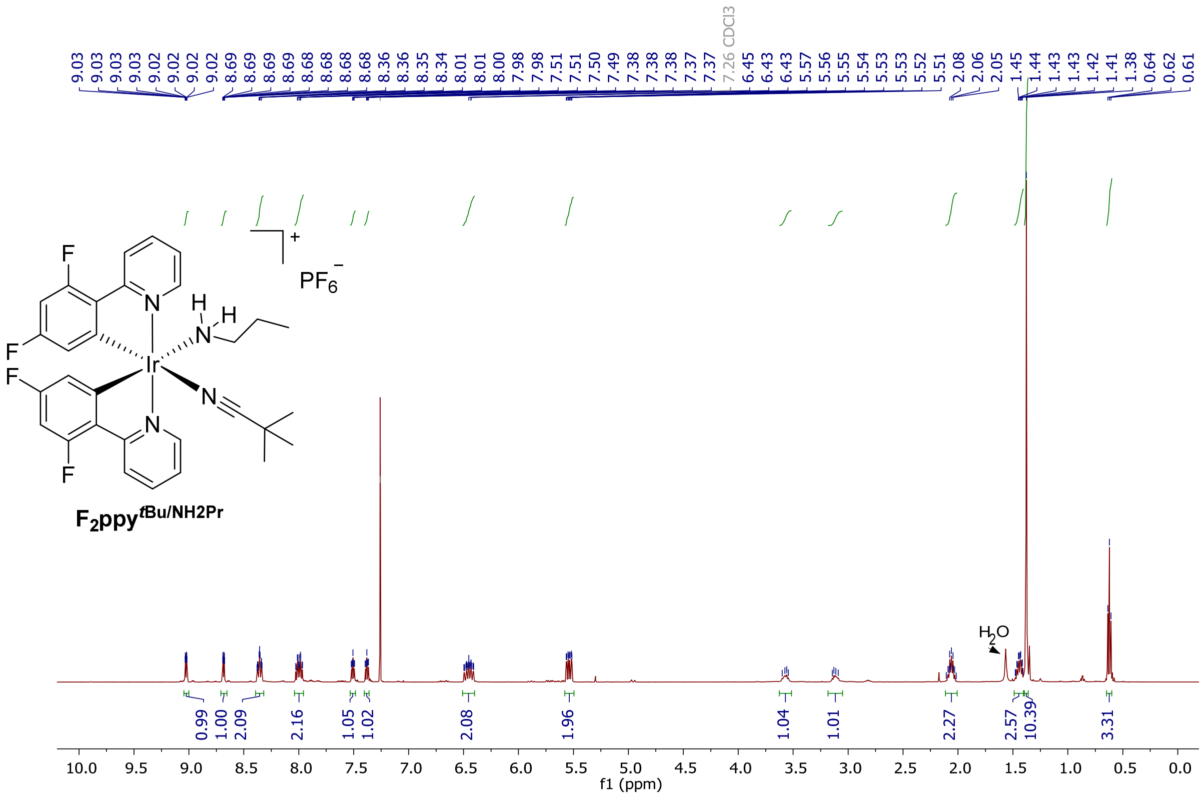

Fig. S28. ^{1}H NMR spectrum of complex **F$_2$ppy$^{tBu/NH2Pr}$**, recorded in chloroform-*d* at 500 MHz.

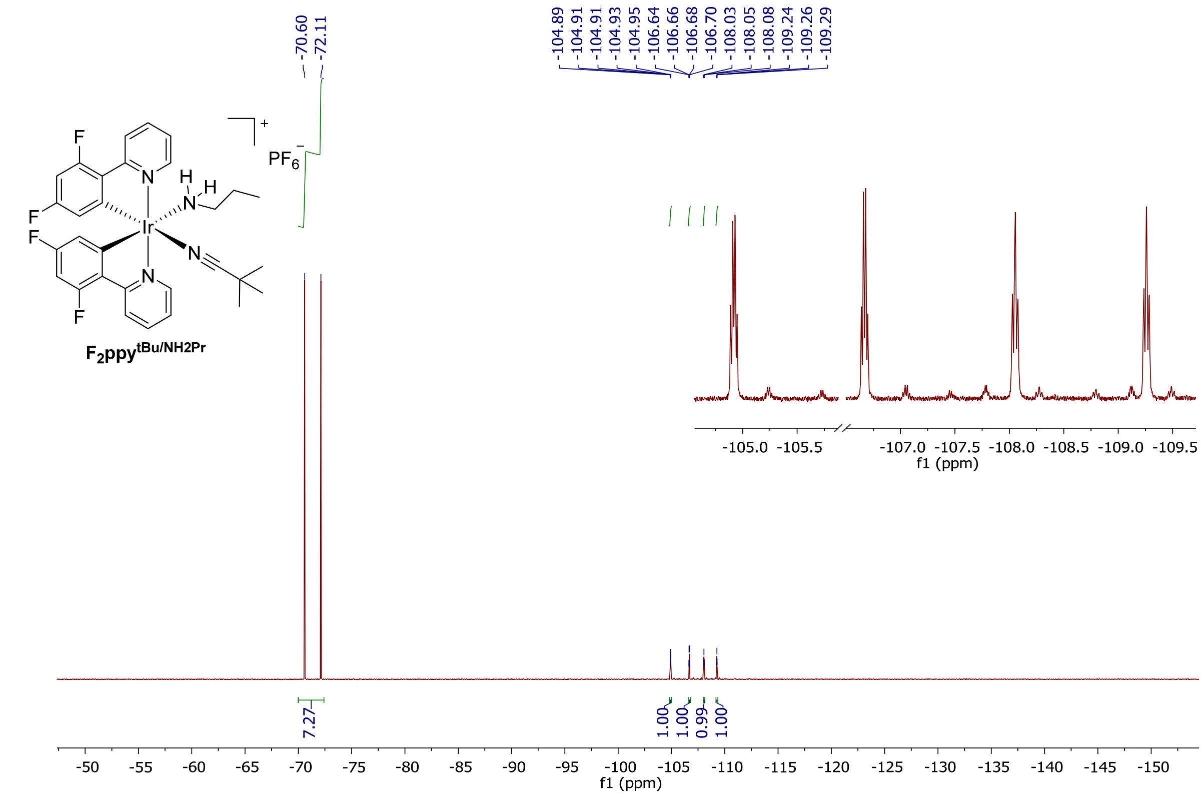

Fig. S29. ^{19}F NMR spectrum of complex **$F_2ppy^{tBu/NH2Pr}$**, recorded in chloroform-*d* at 470 MHz.

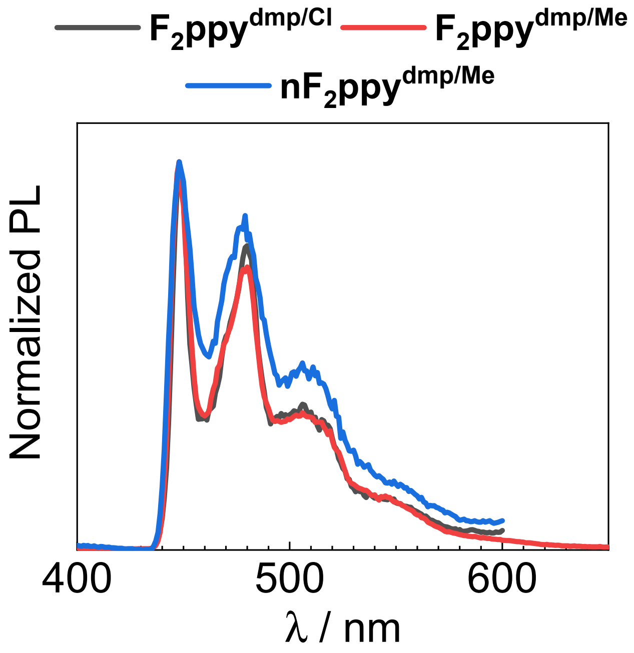

Fig. S30. Overlaid photoluminescence emission spectra of **$F_2ppy^{dmp/Cl}$**, **$F_2ppy^{dmp/Me}$**, and **$nF_2ppy^{dmp/Me}$**, recorded in CH_2Cl_2 at 77 K.

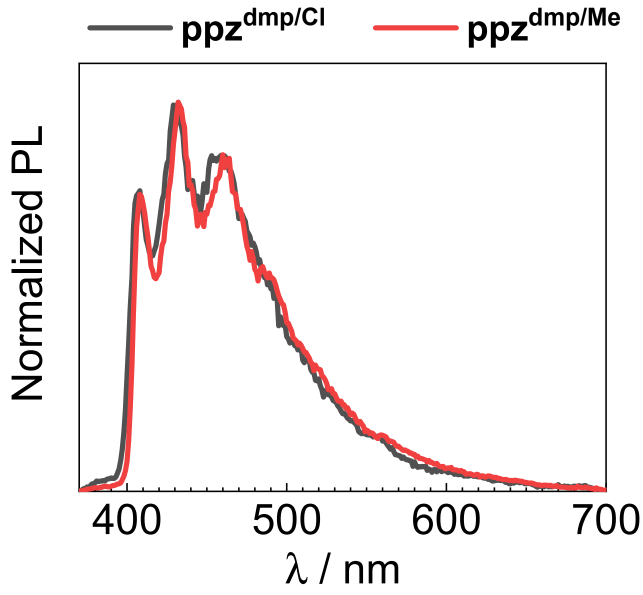

Fig. S31. Overlaid photoluminescence emission spectra of **$ppz^{dmp/Cl}$** and **$ppz^{dmp/Me}$**, recorded in CH_2Cl_2 at 77 K.

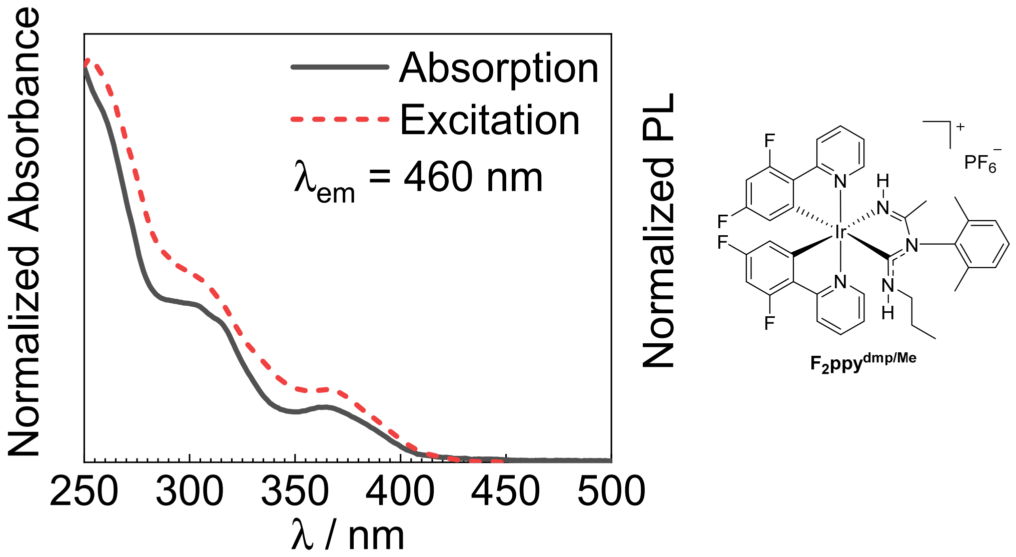

Fig. S32. Overlaid and normalized UV–vis absorption (black solid line) and excitation (red dashed line) spectra of complex **$F_2ppy^{dmp/Me}$**. The UV–vis absorption spectrum was recorded in CH_2Cl_2 and the excitation spectrum in PMMA film at 2 wt%, both at room temperature.

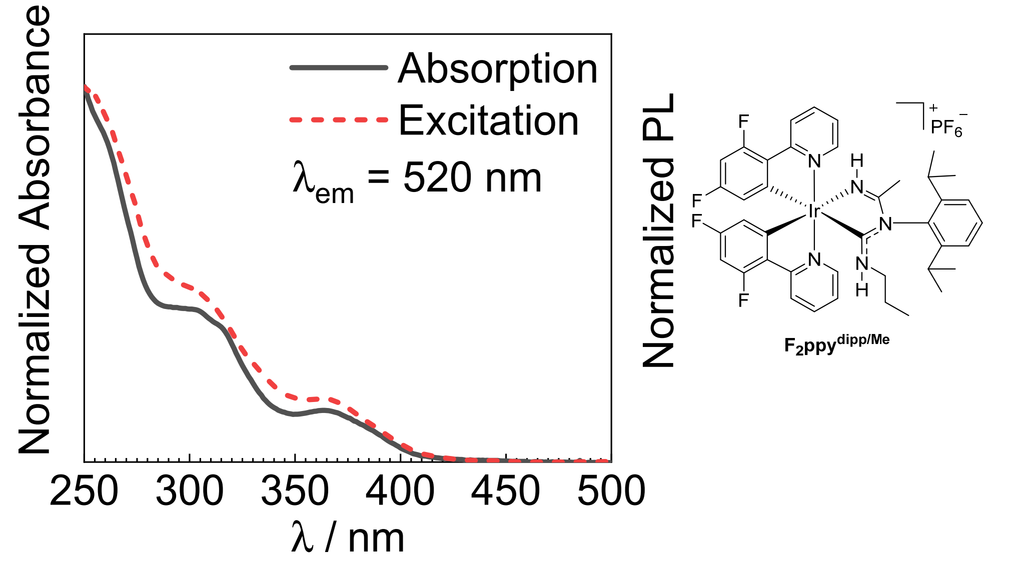

Fig. S33. Overlaid and normalized UV–vis absorption (black solid line) and excitation (red dashed line) spectra of complex **$F_2ppy^{dipp/Me}$**. The UV–vis absorption spectrum was recorded in CH_2Cl_2 and the excitation spectrum in PMMA film at 2 wt%, both at room temperature.

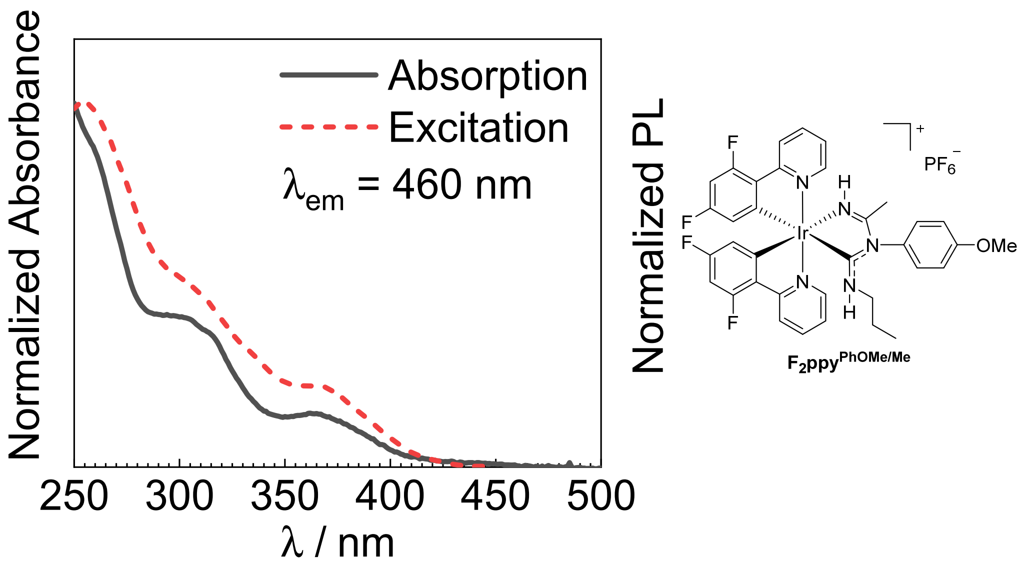

Fig. S34. Overlaid and normalized UV–vis absorption (black solid line) and excitation (red dashed line) spectra of complex $\mathbf{F_2ppy^{PhOMe/Me}}$. The UV–vis absorption spectrum was recorded in CH_2Cl_2 and the excitation spectrum in PMMA film at 2 wt%, both at room temperature.

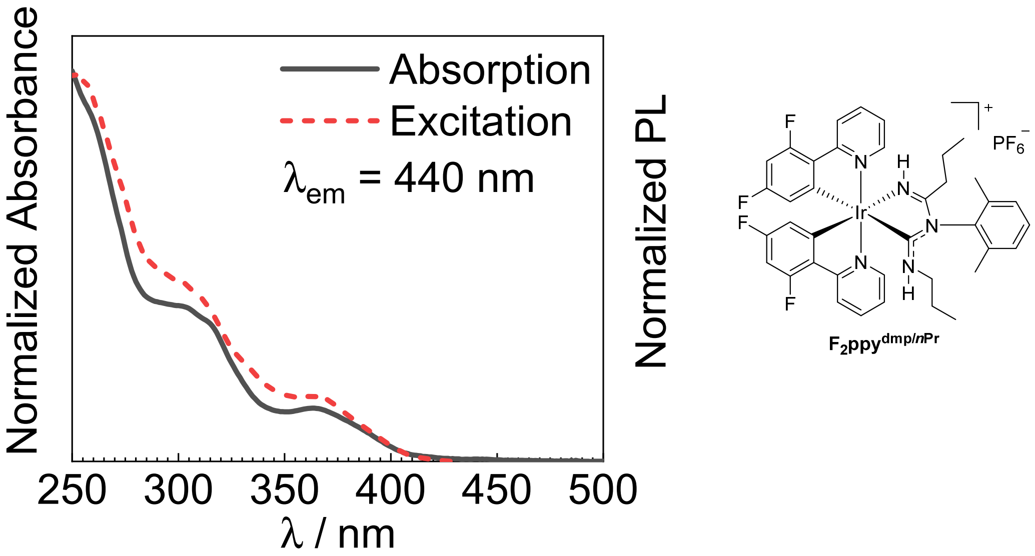

Fig. S35. Overlaid and normalized UV–vis absorption (black solid line) and excitation (red dashed line) spectra of complex $\mathbf{F_2ppy^{dmp/\mathit{n}Pr}}$. The UV–vis absorption spectrum was recorded in CH_2Cl_2 and the excitation spectrum in PMMA film at 2 wt%, both at room temperature.

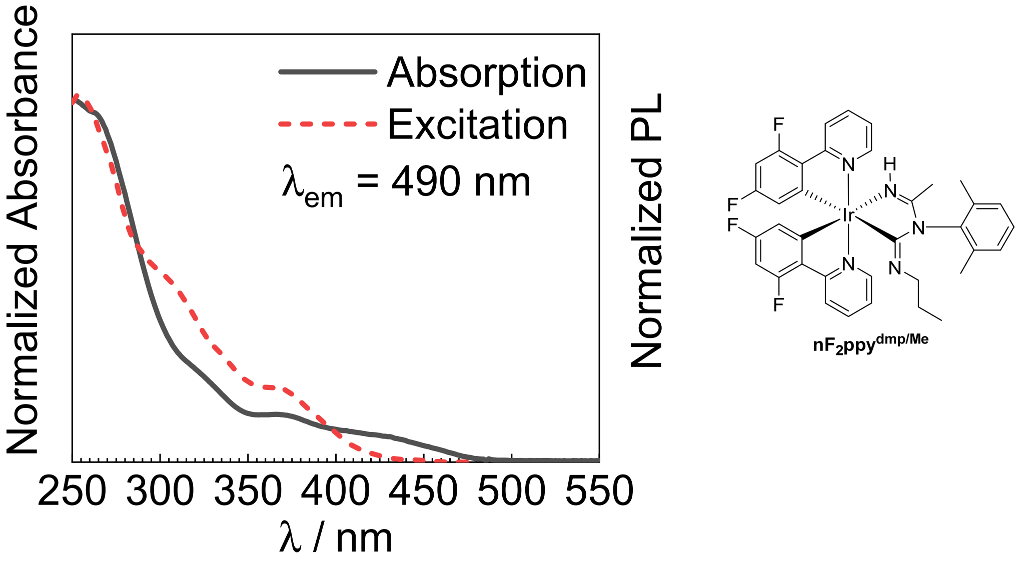

Fig. S36. Overlaid and normalized UV–vis absorption (black solid line) and excitation (red dashed line) spectra of complex **nF2ppy**$^{\mathbf{dmp/Me}}$. The UV–vis absorption spectrum was recorded in CH_2Cl_2 and the excitation spectrum in PMMA film at 2 wt%, both at room temperature.

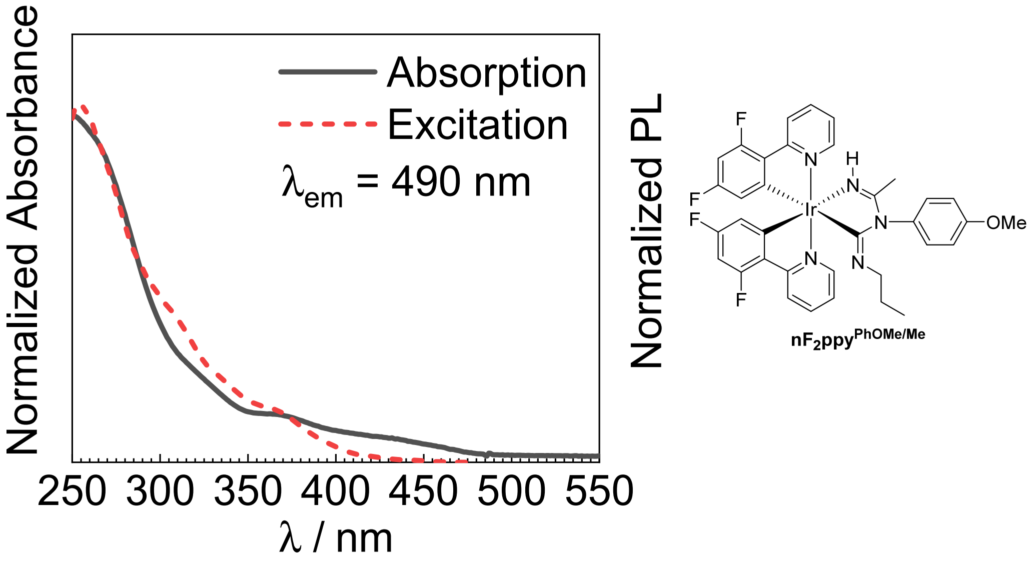

Fig. S37. Overlaid and normalized UV–vis absorption (black solid line) and excitation (red dashed line) spectra of complex **nF2ppy**$^{\mathbf{PhOMe/Me}}$. The UV–vis absorption spectrum was recorded in CH_2Cl_2 and the excitation spectrum in PMMA film at 2 wt%, both at room temperature.

Analysis Report

Sample Information

Name	TS296	Data File	TS296_1.d
Comment		Acq. Time (Lab)	2/27/2026 3:29:35 PM (UTC-06:00)
Instrument	LAKENVELDER	Method (Acq)	Default_directInfusion_Positive.m
Position	P9-A3	Method (DA)	SmallMolecule_2025.m

Sample Chromatograms

Compound Details

Name	Formula	Mass	Species	m/z	Diff (Tgt, ppm)
TS296	C35 H29 F4 Ir N3	758.1892	M+	758.1885	-1.55

Compound Spectra (overlaid)

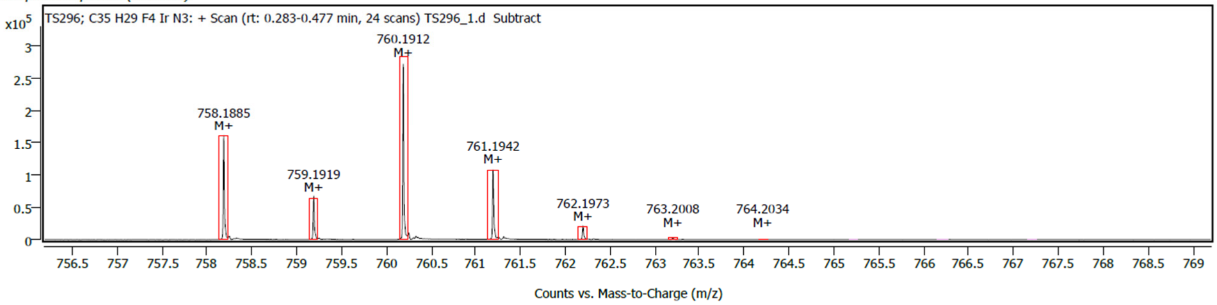

(End of Report)

Fig. S38. ESI-MS accurate mass report of **$F_2ppy^{dipp/Cl}$**.

Analysis Report

Sample Information

Name	TS272	Data File	TS272_1.d
Comment		Acq. Time (Lab)	2/27/2026 3:39:43 PM (UTC-06:00)
Instrument	LAKENVELDER	Method (Acq)	Default_directInfusion_Positive.m
Position	P9-A7	Method (DA)	SmallMolecule_2025.m

Sample Chromatograms

Compound Details

Name	Formula	Mass	Species	m/z	Diff (Tgt, ppm)
TS272	C30 H19 F4 Ir N3	688.1107	M+	688.1101	-2.05

Compound Spectra (overlaid)

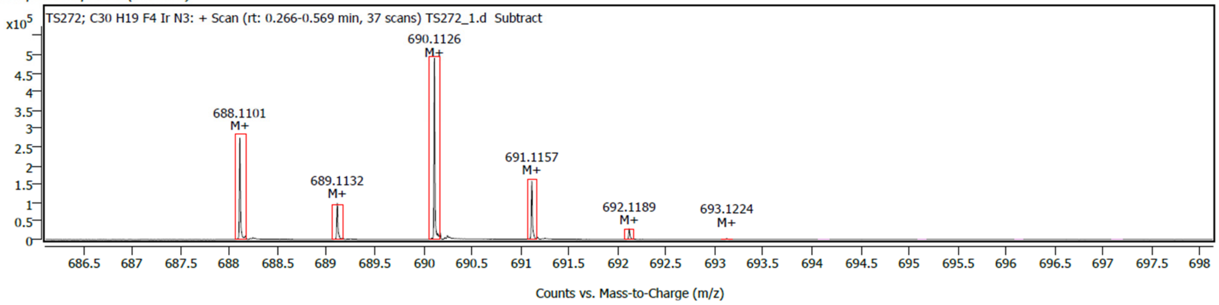

(End of Report)

Fig. S39. ESI-MS accurate mass report of **$F_2ppy^{Bn/Cl}$**.

Analysis Report

Sample Information

**Name**	TS180	**Data File**	TS180_1.d
**Comment**		**Acq. Time (Lab)**	2/27/2026 3:52:23 PM (UTC-06:00)
**Instrument**	LAKENVELDER	**Method (Acq)**	Default_directInfusion_Positive.m
**Position**	P9-B3	**Method (DA)**	SmallMolecule_2025.m

Sample Chromatograms

Compound Details

Name	Formula	Mass	Species	m/z	Diff (Tgt, ppm)
TS180	C27 H23 Ir N5	608.1547	M+	608.1540	-2.09

Compound Spectra (overlaid)

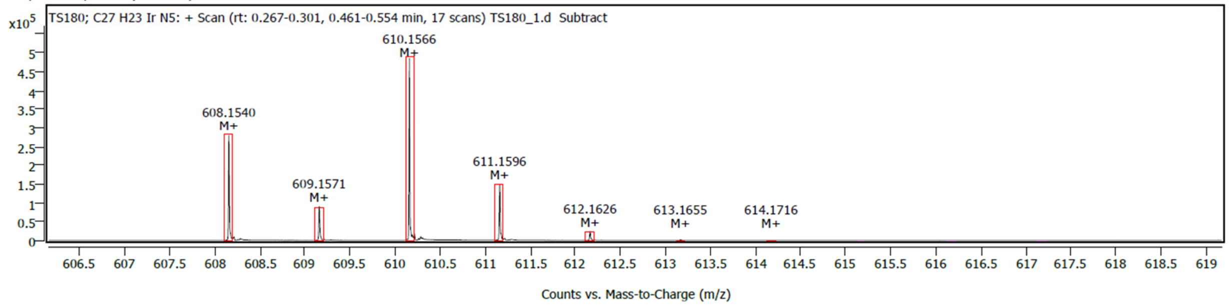

(End of Report)

Fig. S40. ESI-MS accurate mass report of **ppz$^{\mathbf{dmp/Cl}}$**.

Analysis Report

Sample Information

**Name**	TS243	**Data File**	TS243_1.d
**Comment**		**Acq. Time (Lab)**	2/27/2026 3:47:19 PM (UTC-06:00)
**Instrument**	LAKENVELDER	**Method (Acq)**	Default_directInfusion_Positive.m
**Position**	P9-B1	**Method (DA)**	SmallMolecule_2025.m

Sample Chromatograms

Compound Details

Name	Formula	Mass	Species	m/z	Diff (Tgt, ppm)
TS243	C36 H33 F4 Ir N5	802.2263	M+	802.2257	-1.87

Compound Spectra (overlaid)

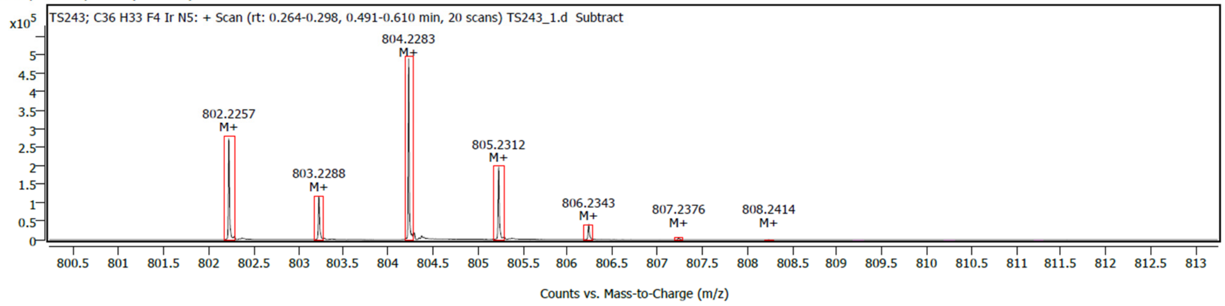

(End of Report)

Fig. S41. ESI-MS accurate mass report of **$\mathbf{F_2ppy^{dmp/Me}}$**.

Analysis Report

Sample Information

**Name**	TS300	**Data File**	TS300_1.d
**Comment**		**Acq. Time (Lab)**	2/27/2026 3:27:03 PM (UTC-06:00)
**Instrument**	LAKENVELDER	**Method (Acq)**	Default_directInfusion_Positive.m
**Position**	P9-A2	**Method (DA)**	SmallMolecule_2025.m

Sample Chromatograms

Compound Details

Name	Formula	Mass	Species	m/z	Diff (Tgt, ppm)
TS300	C40 H41 F4 Ir N5	858.2891	M+	858.2886	-1.48

Compound Spectra (overlaid)

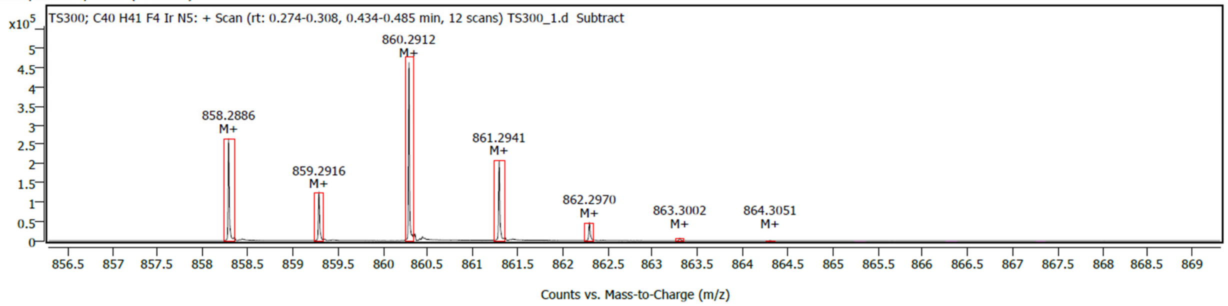

(End of Report)

Fig. S42. ESI-MS accurate mass report of $\mathbf{F_2ppy^{dipp/Me}}$.

Analysis Report

Sample Information

**Name**	TS284	**Data File**	TS284_1.d
**Comment**		**Acq. Time (Lab)**	2/27/2026 3:34:38 PM (UTC-06:00)
**Instrument**	LAKENVELDER	**Method (Acq)**	Default_directInfusion_Positive.m
**Position**	P9-A5	**Method (DA)**	SmallMolecule_2025.m

Sample Chromatograms

Compound Details

Name	Formula	Mass	Species	m/z	Diff (Tgt, ppm)
TS284	C35 H31 F4 Ir N5 O	804.2055	M+	804.2050	-1.91

Compound Spectra (overlaid)

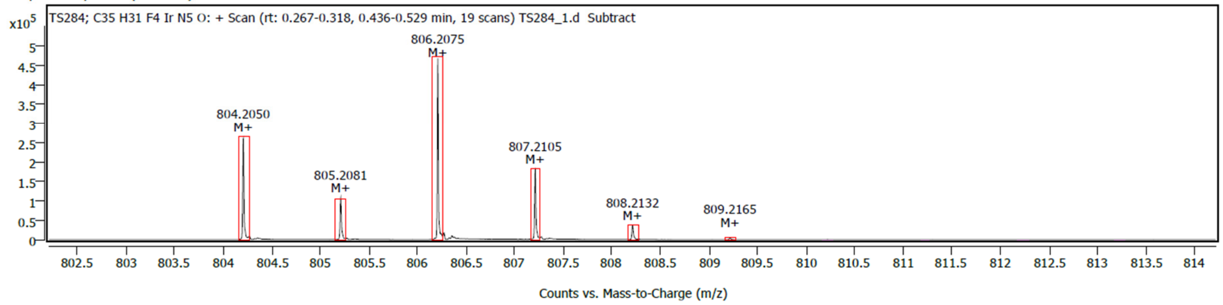

(End of Report)

Fig. S43. ESI-MS accurate mass report of $\mathbf{F_2ppy^{PhOMe/Me}}$.

Analysis Report

Sample Information

**Name**	TS317	**Data File**	TS317_1.d
**Comment**		**Acq. Time (Lab)**	2/27/2026 3:24:31 PM (UTC-06:00)
**Instrument**	LAKENVELDER	**Method (Acq)**	Default_directInfusion_Positive.m
**Position**	P9-A1	**Method (DA)**	SmallMolecule_2025.m

Sample Chromatograms

Compound Details

Name	Formula	Mass	Species	m/z	Diff (Tgt, ppm)
TS317	C38 H37 F4 Ir N5	830.2584	M+	830.2578	-0.78

Compound Spectra (overlaid)

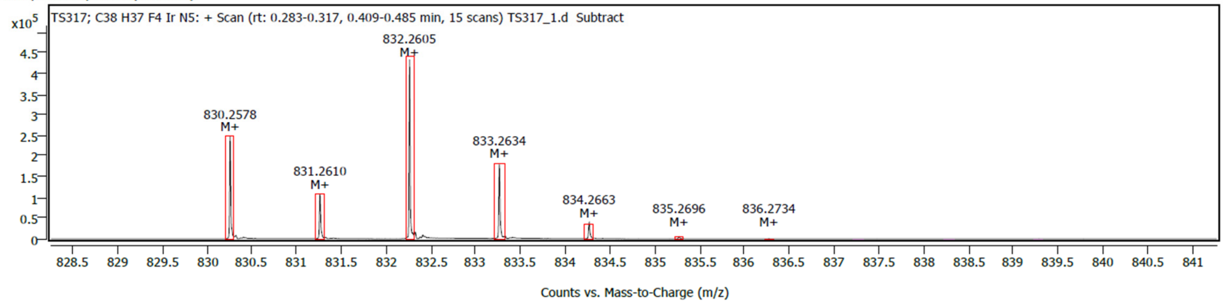

(End of Report)

Fig. S44. ESI-MS accurate mass report of $\mathbf{F_2ppy^{dmp/\mathit{n}Pr}}$.

Analysis Report

ample Information

**ame**	TS266	**Data File**	TS266_1.d
**omment**		**Acq. Time (Lab)**	2/27/2026 3:44:48 PM (UTC-06:00)
**strument**	LAKENVELDER	**Method (Acq)**	Default_directInfusion_Positive.m
**sition**	P9-A9	**Method (DA)**	SmallMolecule_2025.m

ample Chromatograms

ompound Details

ame	Formula	Mass	Species	m/z	Diff (Tgt, ppm)
266	C41 H35 F4 Ir N5	864.2419	M+	864.2413	-1.83

ompound Spectra (overlaid)

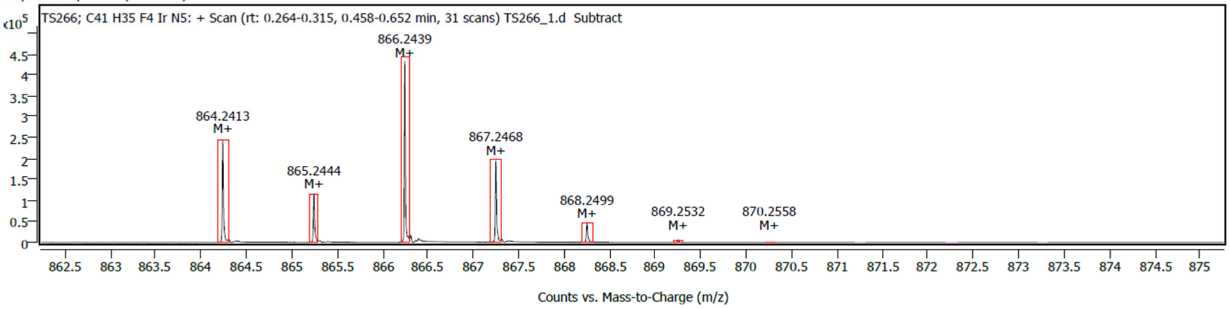

(End of Report)

Fig. S45. ESI-MS accurate mass report of $\mathbf{F_2ppy^{dmp/Ph}}$.

Analysis Report

Sample Information

**Name**	TS219	**Data File**	TS219_1.d
**Comment**		**Acq. Time (Lab)**	2/27/2026 3:49:52 PM (UTC-06:00)
**Instrument**	LAKENVELDER	**Method (Acq)**	Default_directInfusion_Positive.m
**Position**	P9-B2	**Method (DA)**	SmallMolecule_2025.m

Sample Chromatograms

Compound Details

Name	Formula	Mass	Species	m/z	Diff (Tgt, ppm)
TS219	C32 H35 Ir N7	708.2544	M+	708.2538	-2.26

Compound Spectra (overlaid)

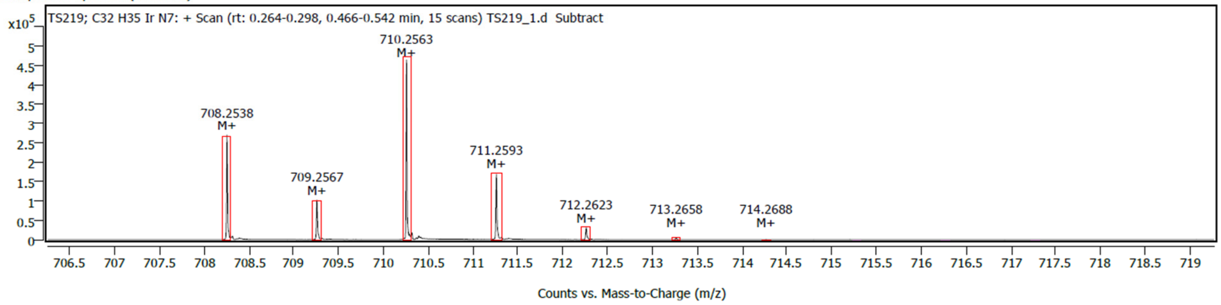

(End of Report)

Fig. S46. ESI-MS accurate mass report of **ppz$^{dmp/Me}$**.

Analysis Report

Sample Information

**Name**	TS280	**Data File**	TS280_1.d
**Comment**		**Acq. Time (Lab)**	2/27/2026 3:37:11 PM (UTC-06:00)
**Instrument**	LAKENVELDER	**Method (Acq)**	Default_directInfusion_Positive.m
**Position**	P9-A6	**Method (DA)**	SmallMolecule_2025.m

Sample Chromatograms

Compound Details

Name	Formula	Mass	Species	m/z	Diff (Tgt, ppm)
TS280	C36 H32 F4 Ir N5	801.2186	(M+H)+	802.2258	-1.76

Compound Spectra (overlaid)

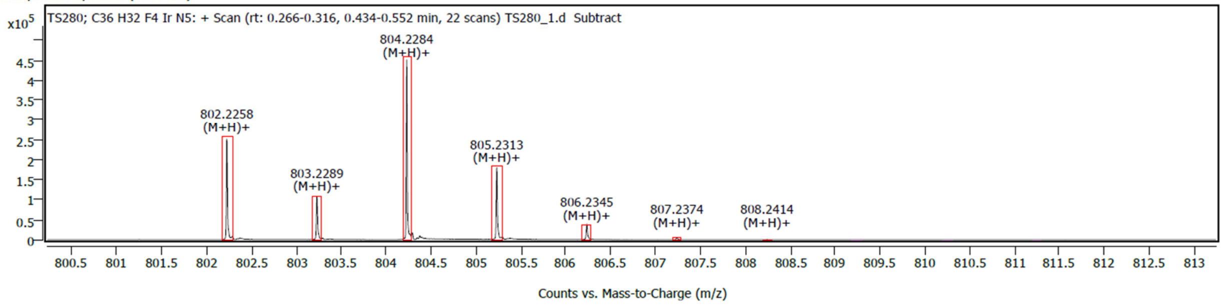

(End of Report)

Fig. S47. ESI-MS accurate mass report of **nF$_2$ppy$^{dmp/Me}$**.

Analysis Report

Sample Information

**Name**	TS288	**Data File**	TS288_1.d
**Comment**		**Acq. Time (Lab)**	2/27/2026 3:32:06 PM (UTC-06:00)
**Instrument**	LAKENVELDER	**Method (Acq)**	Default_directInfusion_Positive.m
**Position**	P9-A4	**Method (DA)**	SmallMolecule_2025.m

Sample Chromatograms

Compound Details

Name	Formula	Mass	Species	m/z	Diff (Tgt, ppm)
TS288	C35 H30 F4 Ir N5 O	803.1982	(M+H)+	804.2054	-1.24

Compound Spectra (overlaid)

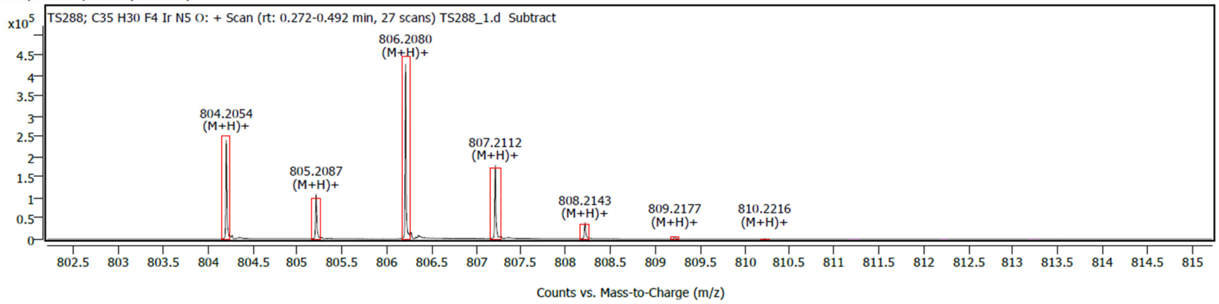

(End of Report)

Fig. S48. ESI-MS accurate mass report of **nF$_2$ppy$^{PhOMe/Me}$**.

Analysis Report

Sample Information

**Name**	TS267	**Data File**	TS267_1.d
**Comment**		**Acq. Time (Lab)**	2/27/2026 3:42:15 PM (UTC-06:00)
**Instrument**	LAKENVELDER	**Method (Acq)**	Default_directInfusion_Positive.m
**Position**	P9-A8	**Method (DA)**	SmallMolecule_2025.m

Sample Chromatograms

Compound Details

Name	Formula	Mass	Species	m/z	Diff (Tgt, ppm)
TS267	C30 H30 F4 Ir N4	713.1999	M+	713.1993	-1.88

Compound Spectra (overlaid)

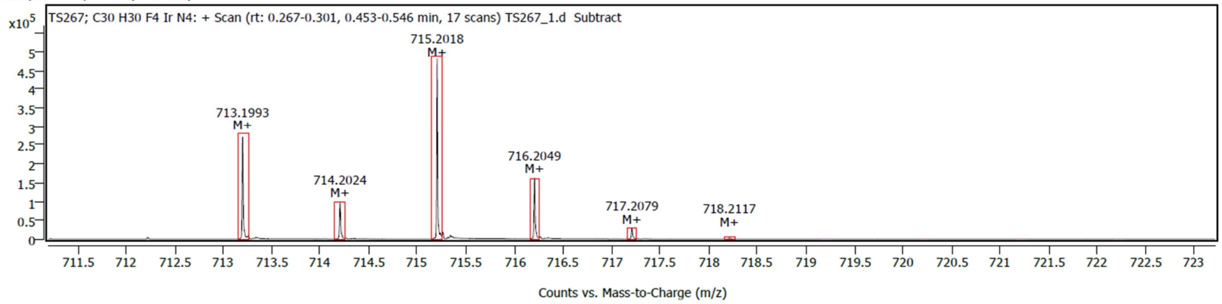

(End of Report)

Fig. S49. ESI-MS accurate mass report of **F$_2$ppy$^{tBu/NH2Pr}$**.

Supplementary Information References

1 H. Na and T. S. Teets, *J. Am. Chem. Soc.*, 2018, **140**, 6353–6360.
2 D. Kim, M. Ahn, K.-R. Wee and D. W. Cho, *Phys. Chem. Chem. Phys.*, 2022, **24**, 13074–13082.
3 G. R. Fulmer, A. J. M. Miller, N. H. Sherden, H. E. Gottlieb, A. Nudelman, B. M. Stoltz, J. E. Bercaw and K. I. Goldberg, *Organometallics*, 2010, **29**, 2176–2179.
4 G. M. Sheldrick, *Acta Crystallogr. Sect. C*, 2015, **71**, 3–8.
5 A. L. Spek, *Acta Crystallogr. D Biol. Crystallogr.*, 2009, **65**, 148–155.
6 K. Dedeian, J. Shi, E. Forsythe, D. C. Morton and P. Y. Zavalij, *Inorg. Chem.*, 2007, **46**, 1603–1611.
7 W. Sattler, M. E. Ener, J. D. Blakemore, A. A. Rachford, P. J. LaBeaume, J. W. Thackeray, J. F. Cameron, J. R. Winkler and H. B. Gray, *J. Am. Chem. Soc.*, 2013, **135**, 10614–10617.
8 A. Maity, J. C. Kölsch, H. Na and T. S. Teets, *Dalton Trans.*, 2017, **46**, 11757–11767.
9 W. P. Weber and G. W. Gokel, *Tetrahedron Lett.*, 1972, **13**, 1637–1640.
